# Half sandwich-type osmium, ruthenium, iridium and rhodium complexes with bidentate glycosyl heterocyclic ligands induce cytostasis in platinum-resistant ovarian cancer cells and bacteriostasis in Gram-positive multiresistant bacteria

**DOI:** 10.3389/fchem.2023.1086267

**Published:** 2023-01-30

**Authors:** István Kacsir, Adrienn Sipos, Tímea Kiss, Evelin Major, Nikolett Bajusz, Emese Tóth, Péter Buglyó, László Somsák, Gábor Kardos, Péter Bai, Éva Bokor

**Affiliations:** ^1^ Department of Organic Chemistry, University of Debrecen, Debrecen, Hungary; ^2^ Doctoral School of Chemistry, University of Debrecen, Debrecen, Hungary; ^3^ Department of Medical Chemistry, Faculty of Medicine, University of Debrecen, Debrecen, Hungary; ^4^ Department of Metagenomics, University of Debrecen, Debrecen, Hungary; ^5^ Department of Inorganic and Analytical Chemistry, Faculty of Sciences and Technology, University of Debrecen, Debrecen, Hungary; ^6^ NKFIH-DE Lendület Laboratory of Cellular Metabolism, Debrecen, Hungary; ^7^ Research Center for Molecular Medicine, Faculty of Medicine, University of Debrecen, Debrecen, Hungary; ^8^ MTA-DE Cell Biology and Signaling Research Group ELKH, Debrecen, Hungary

**Keywords:** half-sandwich complex, glycosyl triazole, quinoline, reactive oxygen species production, ovarian cancer, sarcoma, MRSA, VRE

## Abstract

The toxicity of and resistance to platinum complexes as cisplatin, oxaliplatin or carboplatin calls for the replacement of these therapeutic agents in clinical settings. We have previously identified a set of half sandwich-type osmium, ruthenium and iridium complexes with bidentate glycosyl heterocyclic ligands exerting specific cytostatic activity on cancer cells but not on non-transformed primary cells. The apolar nature of the complexes, conferred by large, apolar benzoyl protective groups on the hydroxyl groups of the carbohydrate moiety, was the main molecular feature to induce cytostasis. We exchanged the benzoyl protective groups to straight chain alkanoyl groups with varying length (3 to 7 carbon units) that increased the IC_50_ value as compared to the benzoyl-protected complexes and rendered the complexes toxic. These results suggest a need for aromatic groups in the molecule. The pyridine moiety of the bidentate ligand was exchanged for a quinoline group to enlarge the apolar surface of the molecule. This modification decreased the IC_50_ value of the complexes. The complexes containing [(η^6^-*p*-cymene)Ru(II)], [(η^6^-*p*-cymene)Os(II)] or [(η^5^-Cp*)Ir(III)] were biologically active unlike the complex containing [(η^5^-Cp*)Rh(III)]. The complexes with cytostatic activity were active on ovarian cancer (A2780, ID8), pancreatic adenocarcinoma (Capan2), sarcoma (Saos) and lymphoma cell lines (L428), but not on primary dermal fibroblasts and their activity was dependent on reactive oxygen species production. Importantly, these complexes were cytostatic on cisplatin-resistant A2780 ovarian cancer cells with similar IC_50_ values as on cisplatin-sensitive A2780 cells. In addition, the quinoline-containing Ru and Os complexes and the short chain alkanoyl-modified complexes (C3 and C4) proved to be bacteriostatic in multiresistant Gram-positive *Enterococcus* and *Staphylococcus aureus* isolates. Hereby, we identified a set of complexes with submicromolar to low micromolar inhibitory constants against a wide range of cancer cells, including platinum resistant cells and against multiresistant Gram-positive bacteria.

## 1 Introduction

Platinum-based compounds play a key role in oncotherapy; cisplatin, oxaliplatin, carboplatin are EMA/FDA registered drugs (Kenny and Marmion, 2019). While the importance of these compounds in not questionable, their applicability is limited by resistance to these drugs and toxicity (Fetoni et al., 2015; Lund et al., 2017; McMullen et al., 2020; Mukherjea et al., 2020; Yu et al., 2020) that calls for the development of novel drugs. A perspective drug class for the replacement of platinum-based drugs is the organometallic complexes of other platinum-group metals such as complexes of ruthenium (MelChart and Sadler, 2005; Hartinger et al., 2011; Leijen et al., 2015; Burris et al., 2016; Zeng et al., 2017; Gichumbi and Friedrich, 2018; Meier-Menches et al., 2018; Štarha and Trávníček, 2019; Kacsir et al., 2021; Kulkarni et al., 2022) osmium (Hartinger et al., 2011; Hanif et al., 2014; Gichumbi and Friedrich, 2018; Konkankit et al., 2018; Meier-Menches et al., 2018; Štarha and Trávníček, 2019; Nabiyeva et al., 2020; Li et al., 2021b; Balázs et al., 2022; Kacsir et al., 2022), iridium (Leung et al., 2013; Liu and Sadler, 2014; Gichumbi and Friedrich, 2018; Konkankit et al., 2018; Štarha and Trávníček, 2019; Kacsir et al., 2022) or rhodium (Leung et al., 2013; Gichumbi and Friedrich, 2018; Máliková et al., 2021). Compounds can be coupled to bait molecules [e.g., biotin, transferrin, hormones or carbohydrates (Hartinger et al., 2008; Fernandes, 2019; Kenny and Marmion, 2019; Bononi et al., 2020)] that improves specific targeting and can limit toxicity. Furthermore, better toxicity profile was reported for these compounds as compared to platinum-based drugs (Mello-Andrade et al., 2018; Gano et al., 2019; Liu et al., 2019; Mihajlovic et al., 2020) in good agreement with the fact that NAMI-A (Leijen et al., 2015), KP1019/1339 (IT-139, BOLD100) (Burris et al., 2016) or TLD-1433 (Kulkarni et al., 2022) compounds are already in different phases of clinical trials against neoplastic diseases as bladder or lung cancer. Of note, NAMI-A and KP-1019 contain monodentate ligands (similar to cisplatin), while in TLD-1433 the central metal ion is coordinated by a bidentate chelating ligand.

**FIGURE 1:**
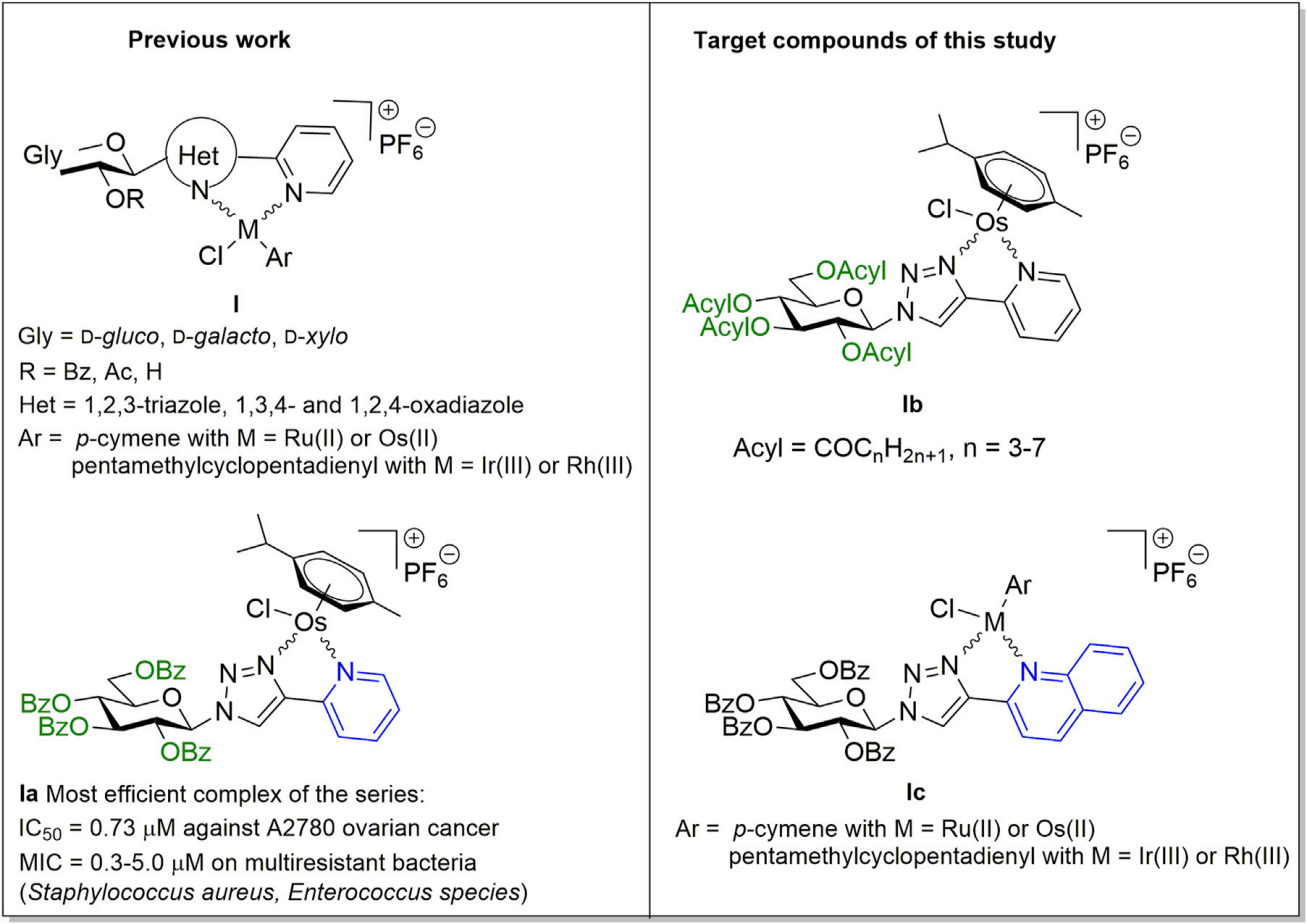
Preliminaries based on our previous studies (Kacsir et al., 2021; Balázs et al., 2022; Kacsir et al., 2022), and target compounds of this work.

Recently, we have identified a series of monosaccharide-heterocycle conjugates as ligands that yield bioactive complexes with Ru(II), Os(II) and Ir(III) (Kacsir et al., 2021; Balázs et al., 2022; Kacsir et al., 2022) (Figure 1, **I**). The complexes had cytostatic properties with specificity for transformed cancer cell lines including a large set of carcinomas (ovarian and breast cancer, pancreatic adenocarcinoma and glioblastoma), lymphoma and sarcoma cell lines (Kacsir et al., 2021; Kacsir et al., 2022). Furthermore, the compounds were bacteriostatic against isolates of Gram-positive vancomycin-resistant *Enterococcus* (VRE) and methicillin-resistant *Staphylococcus aureus* (MRSA) (Balázs et al., 2022). *C*-Glycosyl 1,3,4-oxadiazole ligands exhibit low micromolar IC_50_ or MIC values against cancer cells or bacteria, respectively. Replacing the oxadiazole moiety to an *N*-glycosidically linked 1,2,3-triazole yielded more efficient compounds (e.g., **Ia**) with submicromolar IC_50_ values and submicromolar to low micromolar MIC values on the multiresistant clinical VRE/MRSA isolates (Kacsir et al., 2021; Balázs et al., 2022; Kacsir et al., 2022).

The activity of the complexes was dependent on the generation of reactive oxygen species (ROS) (Kacsir et al., 2021; Kacsir et al., 2022) similar to other ruthenium complexes (Xu et al., 2018; Fernandes, 2019; Bakewell et al., 2020; Mihajlovic et al., 2020) and partially on PARP activation, again, a feature observed for ruthenium complexes (Xu et al., 2018; de Camargo et al., 2019; Bakewell et al., 2020; Yusoh et al., 2020). Another feature that played key role in the biological activity of the complexes was cooperative binding that was deduced from the Hill-coefficient of the inhibitory curves (Kacsir et al., 2021; Kacsir et al., 2022). Hill coefficient above 1 suggests cooperative binding (Gesztelyi et al., 2012), the compounds we identified earlier had Hill coefficients >1.5 (Kacsir et al., 2021; Kacsir et al., 2022).

In addition, the active compounds had a lipophilic character. The *O*-perbenzoylated forms of the glycosyl moiety were active in contrast to *O*-peracetylated and *O*-unprotected forms (Kacsir et al., 2021; Kacsir et al., 2022). The exchange of the *O*-perbenzoylated glycosyl moiety for a phenyl group disrupted the biological activity of the compounds (Kacsir et al., 2021). These results point out the importance of the carbohydrate moiety, its protective groups and the overall lipophilic character of the complexes.

In this study we have carried out structure-activity relationship investigations to assess whether the structure of the protective groups or modification of the heterocycle distal to the carbohydrate moiety affected the biological activity of the complexes. Namely, the replacement of the *O*-benzoyl protecting groups of the most effective complex **Ia**, identified in our previous studies (Balázs et al., 2022; Kacsir et al., 2022), by aliphatic acyl protecting groups with homologously increasing chain-length (**Ib**) has been accomplished (Figure 1). In addition, an increase of the size of the sugar aglycon part in **Ia** and its Ru(II), Ir(III) and Rh(III) analogs (Balázs et al., 2022; Kacsir et al., 2022) by changing the pyridine ring to quinoline (**Ic**) has also been performed (Figure 1).

## 2 Results

### 2.1 Chemistry

First, a series of *O*-peracylated 1-(β-D-glucopyranosyl)-4-(pyridin-2-yl)-1,2,3,-triazoles was prepared. Gentle heating of the corresponding unprotected glucosyl heterocycle **1** (Kacsir et al., 2021) with aliphatic carboxylic acid chlorides in pyridine furnished the desired triazoles **L-2‒L-6** in good yields (Table 1). These compounds were then incorporated as *N*,*N*-bidentate ligands into [(η^6^-*p*-cym)Os^II^(N-N)Cl]PF_6_ type half-sandwich complexes by their reactions with dichloro(η^6^-*p-*cymene)osmium(II) dimer ([(η^6^-*p*-cym)OsCl_2_]_2_, **Os-dimer**) and TlPF_6_. Similar to our previous studies on the synthesis of analogous half-sandwich complexes (Kacsir et al., 2021; Kacsir et al., 2022), the new test compounds **Os-2‒Os-6** were obtained in good to excellent yields as mixtures of two diastereoisomers (Table 1).

**TABLE 1:** Synthesis of *O*-peracylated 1-(β-D-glucopyranosyl)-4-(pyridin-2-yl)-1,2,3-triazoles and their [(η^6^-*p*-cym)Os^II^(N-N)Cl]PF_6_ compexes.

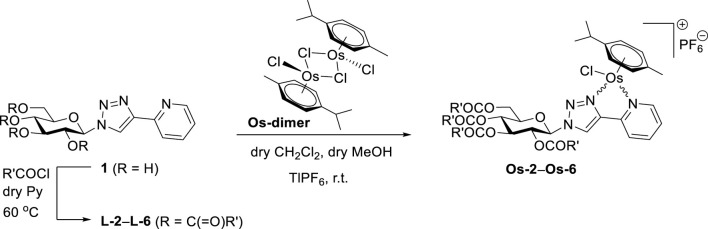					
R’	Ligand	Yield (%)	Complex	Yield (%)	Diastereomeric ratio
-CH_2_CH_2_CH_3_	**L-2**	87	**Os-2**	96	1 : 1
-CH_2_CH_2_CH_2_CH_3_	**L-3**	55	**Os-3**	88	4 : 3
-CH_2_CH_2_CH_2_CH_2_CH_3_	**L-4**	71	**Os-4**	80	5 : 4
-CH_2_CH_2_CH_2_CH_2_CH_2_CH_3_	**L-5**	80	**Os-5**	53	9 : 7
-CH_2_CH_2_CH_2_CH_2_CH_2_CH_2_CH_3_	**L-6**	81	**Os-6**	72	5 : 4

**FIGURE 2:**
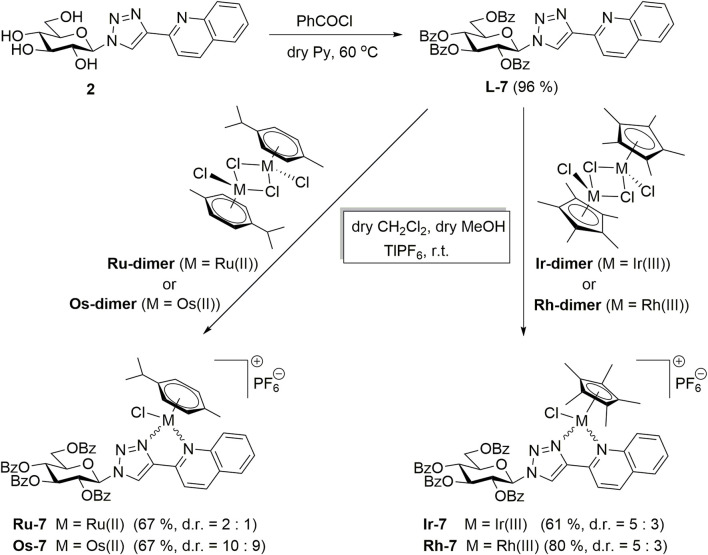
Synthesis of 1-(2’,3’,4’,6’-tetra-*O*-benzoyl-β-D-glucopyranosyl)-4-(quinolin-2-yl)-1,2,3-triazole and its [(η^6^-*p*-cym)M^II^(N-N)Cl]PF_6_ (M = Ru, Os) and [(η^5^-Cp*)M^III^(N-N)Cl]PF_6_ (M = Ir, Rh) compexes.

Next, the synthesis of 1-(2’,3’,4’,6’-tetra-*O*-benzoyl-β-D-glucopyranosyl)-4-(quinolin-2-yl)-1,2,3,-triazole (**L-7**) was carried out starting from the *O*-unprotected derivative **2** (Kacsir et al., 2021) by using standard *O*-perbenzoylation conditions (Figure 2). *p*-Cymene-containing Ru(II) and Os(II) and pentamethylcyclopentadienyl containing Ir(III) and Rh(III) cationic half-sandwich complexes with PF_6_^−^ counter ion were then prepared by the treatment of **L-7** with the appropriate dimeric platinum-group metal chloride precursors (**Ru/Os-dimer** and **Ir/Rh-dimer**, respectively) in the presence of TlPF_6_ (Figure 2).

### 2.2 Biological characterization of the complexes

#### 2.2.1 Assessment of the complexes with alkanoyl protective groups

First, we assessed a set of osmium complexes (similar to **Ia** [Kacsir et al., 2022) in Figure 1] and their sugar-derived ligands, in which the benzoyl protective groups on the carbohydrate moiety were replaced by aliphatic acyl groups of different chain length (straight chain C_3_-C_7_–CO). Osmium was selected as a central ion, since osmium complexes had the best biological activity in our previous studies (Balázs et al., 2022; Kacsir et al., 2022). These compounds were tested on A2780 ovarian cancer cells for toxicity and cytostasis. The therapy of ovarian cancer is centered around platinum compounds (Brown et al., 2019; Sipos et al., 2021), therefore, it was logical to use a cellular model of ovarian cancer for the initial testing of the compounds. Toxicity was assessed using the MTT assay that measures the activity of mitochondrial complex I. We chose 4 h long treatment as in an MTT assay this time point is suitable to detect rapid, direct toxicity. Cytostasis was assessed after 48 h of treatment using the SRB assay that assesses protein content that corresponds to cell count. The characteristics of the compounds are detailed in Table 2.

**TABLE 2:** Numeric values characterizing the complexes assessed in this study. The IC_50_ value of cisplatin was obtained from (Kacsir et al., 2021). Max—Maximal inhibition in %, ND—not detected.

	A2780						ID8						cisplatin resistant A2780						Capan2			SAOS			L428			Fibroblast						LogD
	MTT			SRB			MTT			SRB			MTT			SRB			SRB			SRB			SRB			MTT			SRB			
	Max.	IC_50_ (µM)	Hill	Max.	IC_50_ (µM)	Hill	Max.	IC_50_ (µM)	Hill	Max.	IC_50_ (µM)	Hill	Max.	IC_50_ (µM)	Hill	Max.	IC_50_ (µM)	Hill	Max.	IC_50_ (µM)	Hill	Max.	IC_50_ (µM)	Hill	Max.	IC_50_ (µM)	Hill	Max.	IC_50_	Hill	Max.	IC_50_ (µM)	Hill	
**L-2**	ND	ND	ND	21.2	ND	ND																												ND
**Os-2**	88	ND	ND	>90	6.23	2.25																												2.5
**L-3**	ND	ND	ND	32.0	ND	ND																												ND
**Os-3**	84.4	ND	ND	>90	2.10	2.50																												2.6
**L-4**	ND	ND	ND	26.9	ND	ND																												ND
**Os-4**	84.4	ND	ND	>90	3.87	2.41																												2.7
**L-5**	ND	ND	ND	28.5	ND	ND																												ND
**Os-5**	83.4	ND	ND	>90	15.9	2.33																												2.7
**L-6**	insoluble																																	ND
**Os-6**	76.9	16.5	1.89	88.4	ND	ND																												2.8
**L-7**	ND	ND	ND	16.9	ND	ND	ND	ND	ND	ND	ND	ND																ND	ND	ND	ND	ND	ND	ND
**Ru-7**	23.4	ND	ND	>90	0.847	1.84	32.56	ND	ND	>90	1.14	3.78	ND	ND	ND	>90	1.18	1.82	39.80	1.39	2.08	33.64	1.18	5.16	52.73			23.93	ND	ND	50.04	ND	ND	2.21
**Os-7**	33.0	ND	ND	>90	0.578	1.80	74.90	ND	ND	>90	0.359	4.48	ND	ND	ND	>90	0.426	1.17	59.11	1.35	1.26	48.04	1.29	1.22	>90			30.97	ND	ND	48.94	ND	ND	2.33
**Ir-7**	23.2	ND	ND	>90	0.891	1.84	19.23	ND	ND	>90	0.799	2.98	ND	ND	ND	>90	1.535	2.40	48.83	1.93	1.78	33	1.58	2.98	>90			12.81	ND	ND	42.83	ND	ND	2.17
**Rh-7**	18.3	ND	ND	47.9	ND	ND	ND	ND	ND	ND	ND	ND																15.09	ND	ND	ND	ND	ND	1.6
Cisplatin	ND	ND	ND	>90	1.21	1.20							ND	ND	ND	>90	16.47	ND																

**FIGURE 3:**
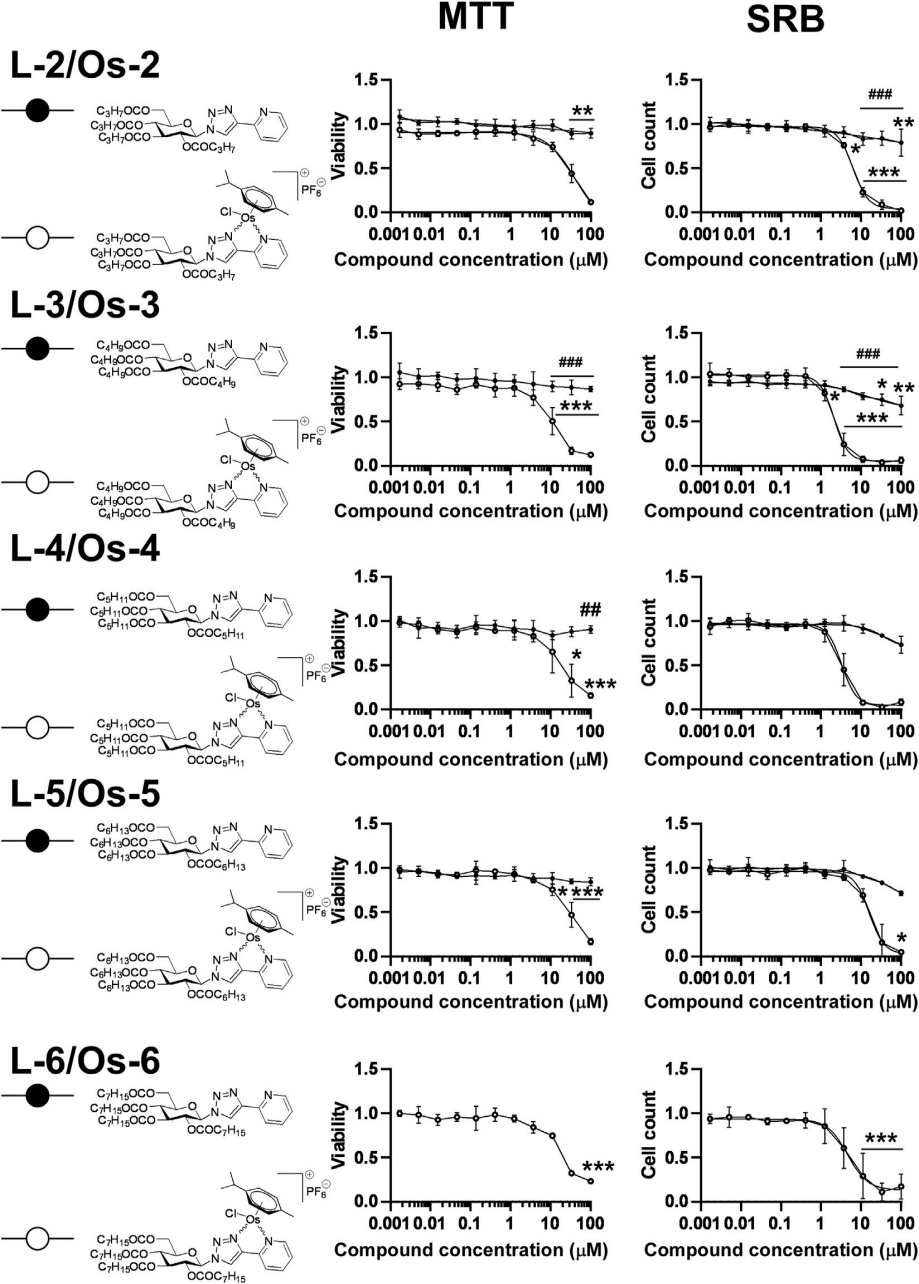
Assessment of free ligands **L-2**—**L-6** and osmium complexes **Os-2**—**Os-6** for cytotoxic and cytostatic activity. 3 × 10^3^ A2780 cells, were plated to 96 well plates. Cells were treated with the compounds in the concentrations indicated for either 4 h for an MTT assay or for 48 h for an SRB assay. Data is represented as average ± SD, from three biological replicates; individual assays were performed in duplicates. Values were normalized for vehicle treated cells, absorbance for vehicle treated cells equals to 1. The MTT dataset for **Os-3+L-3** and the SRB datasets for **Os-2+L-2, Os-3+L-3** showed normal distribution, the MTT datasets for **Os-2+L-2, Os-4+L-4, Os-5+L-5, Os-6** and the SRB dataset for **Os-4+L-4, Os-5+L-5, Os-6** was normalized using the Box-Cox method. Except for the **Os-6** complex, Two-way ANOVA test was performed and all values were compared with each other (Tukey’s *post hoc* test). For **Os-6** One-way ANOVA was performed on Box-Cox normalized values followed by Dunnett’s *post hoc* comparing all values to the smallest treatment concentration. *, ** and *** indicate statistically significant differences between vehicle-treated (control) and the cells treated with a compound at *p* <0.05, *p* <0.01 and *p* <0.001, respectively. ## and ### indicate statistically significant differences between the free ligand and the corresponding complex at *p* <0.01 and *p* <0.001, respectively. Non-linear regression was performed on the data.

**FIGURE 4:**
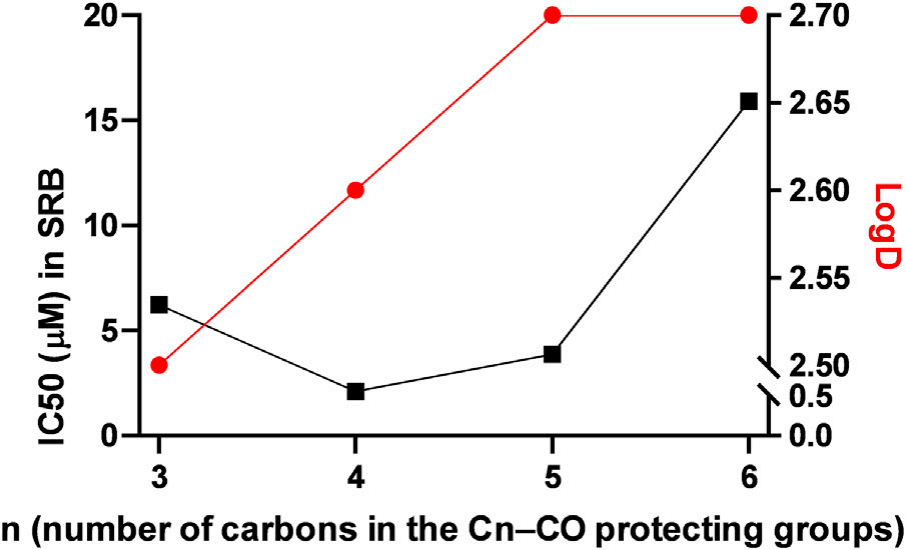
Correlation between the logD and the IC_50_ values in alkanoyl-protected complexes. The black line represents the IC_50_ values, while the red line represents the logD values.

The compounds had high logD value indicating a strong apolar character up to the point that the free ligand **L-6** with the longest C_7_H_15_–CO alkanoyl protective groups proved to be insoluble, hence, it was not suitable for testing. All complexes **Os-2‒Os-6**, but not the free ligands **L-2‒L-5**, exerted rapid toxicity on A2780 cells in MTT assays (Figure 3; Table 2) with cytotoxicity over 80%. Long term treatment of A2780 cells with the *O*-alkanoylated compounds resulted in lower cell proliferation with IC_50_ values in the micromolar range (Figure 3; Table 2). The Hill coefficients, highlighting binding mode of the compounds (Gesztelyi et al., 2012), were above two suggesting cooperative binding of the test substances (Table 2). The free ligands had no more than 30% inhibition at the peak concentration (Figure 3; Table 2). When the IC_50_ value of the complexes were plotted against the logD values we found that the optimum alkyl chain length to provide the best IC_50_ value was 4 carbons long (Figure 4).

Using alkanoyl protective groups instead of benzoyl groups we published earlier (Kacsir et al., 2021; Kacsir et al., 2022) showed that the aliphatic acyl protection significantly deteriorated the biological properties of the complexes. Namely, the new compounds exerted rapid toxicity and their IC_50_ values were higher than that of **Ia** in (Kacsir et al., 2022) (0.73 µM for **Ia** vs. 2.104 µM for **Os-3** in this study). Therefore, these complexes were omitted from further testing as anticancer agents.

#### 2.2.2 Complexes with quinoline heterocycle are cytostatic but not cytotoxic

**FIGURE 5:**
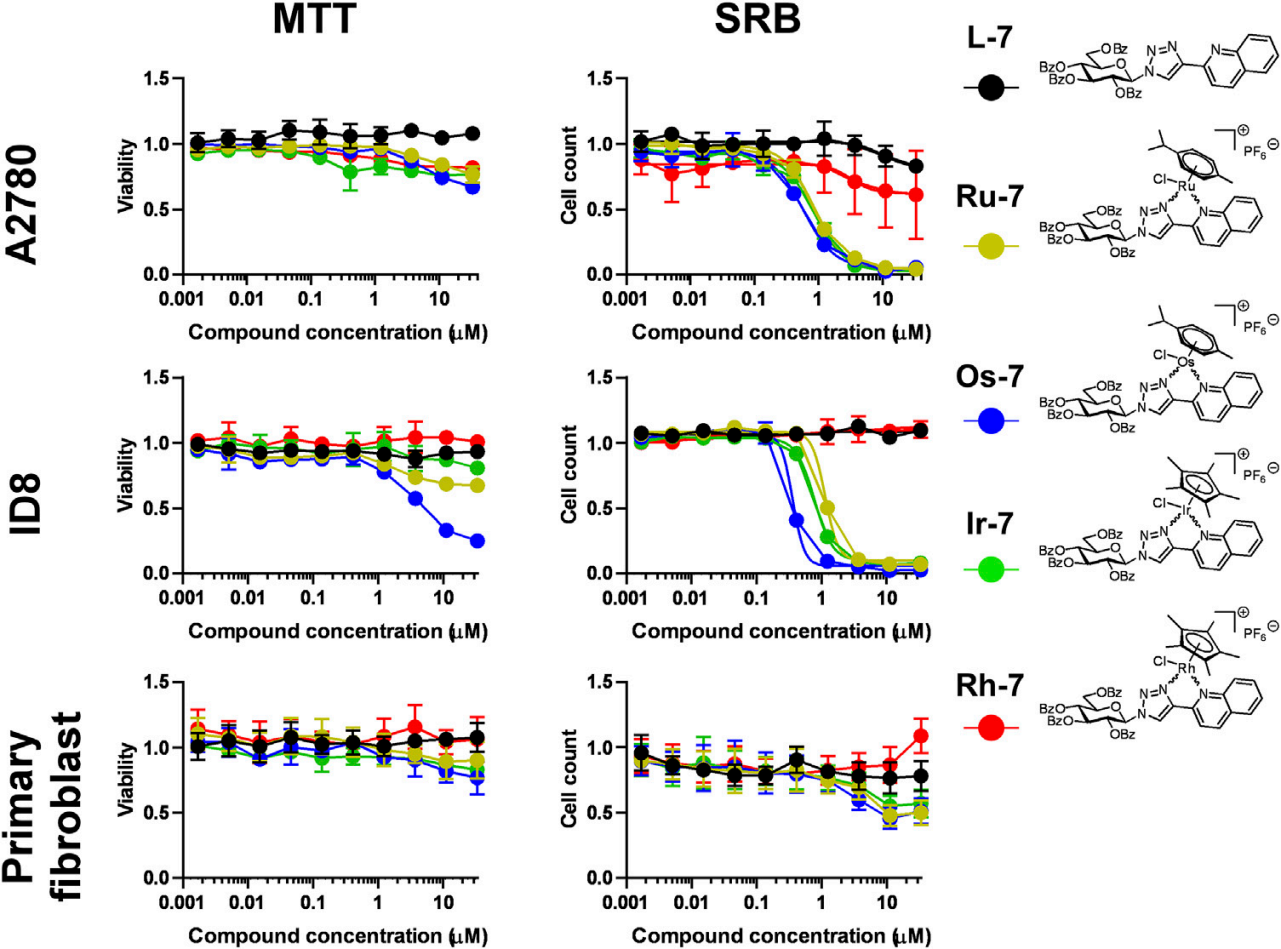
Assessment of quinoline-containing complexes **Ru-7, Os-7, Ir-7, Rh-7** and the corresponding free ligand **L-7** for cytotoxic and cytostatic activity. 3 × 10^3^ A2780 cells, 2 × 10^3^ ID8 cells and 4 × 10^3^ primary fibroblasts were plated to 96 well plates. Cells were treated with the compounds in the concentrations indicated for either 4 h for an MTT assay or for 48 h for an SRB assay. Data is represented as average ± SD, from three biological replicates; individual assays were performed in duplicates. Values were normalized for vehicle treated cells, absorbance for vehicle treated cells equals to 1. Statistical significance was assessed using two-way ANOVA test comparing the free ligand and each complex as a complex-ligand pair. Before the test normality was assessed using the D’Agostino-Pearson test. The ID8 SRB **Ir-7+L-7** dataset had lognormal distribution. The A2780 MTT **Ru-7+L-7**, A2780 SRB **Os-7+L-7**, Fibroblast SRB **Ru-7+L-7** were transformed using the Box-Cox method to achive normal distribution. All other datasets had normal distribution. For better visibility the *p* values are presented in an excel sheet at https://figshare.com/s/2c942a812caea8869a67. Non-linear regression was performed on the A2780 SRB and the ID8 SRB datasets.

Next, the quinoline-containing complexes **Ru-7, Os-7, Ir-7, Rh-7** were tested on A2780 and ID8 ovarian cancer cells, and on non-transformed primary human fibroblasts, used as controls, in a concentration range up to 33.3 µM (Figure 5). In concentrations exceeding 33.3 µM the complexes frequently precipitated. All of the complexes and the free ligand exerted little rapid cytotoxicity in MTT assays, not more than 35% in any of the cell lines assessed in the peak concentration, except for the **Os-7** complex with 75% toxicity on ID8 cells (Figure 5; Table 2). In SRB assays that detects cytostasis, the **Ru-7**, **Os-7** and **Ir-7** complexes completely blocked cell proliferation with submicromolar or low micromolar IC_50_ values both in A2780 and ID8 cells (Figure 5; Table 2). The complexes were more efficient in ID8 cells as compared to A2780 cells (Figure 5; Table 2). The free ligand **L-7** and the **Rh-7** complex had only little cytostatic activity both in A2780 and ID8 cells (Figure 5; Table 2). On primary non-transformed fibroblasts, used as controls, the complexes exerted negligible toxicity and low cytostatic activity (Figure 5; Table 2). The cytostatic activity was not more than 50% in the peak concentration. Similar to our previous results (Kacsir et al., 2021; Kacsir et al., 2022) the complexes had Hill coefficients >1 (Table 2) suggesting cooperative binding to cellular target molecule(s).

**FIGURE 6:**
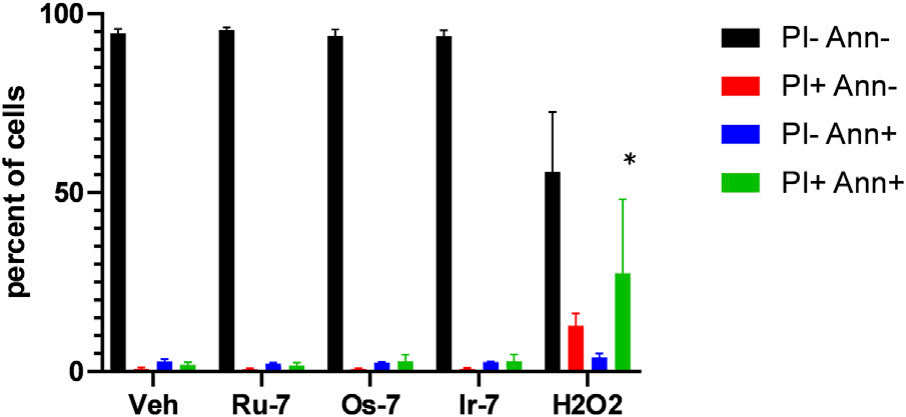
Quinoline complexes do not induce cell death. 2 × 10^6^ A2780 cells were plated in 12 well plates and were treated with the complexes (**Ru-7** at 0.846 μM, **Os-7** at 0.578 µM and **Ir-7** at 0.891 µM) and 300 µM hydrogen peroxide for 2 h. Cells were then stained with Annexin V and propidium iodide (PI) and cells were subjected to flow cytometry as described in Materials and Methods. The percent of cells in the quadrants are plotted. Data is represented as average ± SD, from three biological replicates, individual assays were performed in duplicates. Normal distribution was achieved by Box-Cox normalization of the data. Statistical significance was determined using a two-way ANOVA test, all measurement points were compared with each other. * indicate statistically significant differences between vehicle-treated (control) and treated cells (complexes or 300 µM H_2_O_2_) corresponding to the same quadrant (e.g., vehicle-treated double negative cells vs. H_2_O_2_-treated double negative cells) at *p* <0.05.

We verified the toxic effects of the active Ru, Os and Ir complexes by Annexin V-FITC propidium iodide (PI) double staining. The complexes were applied in concentrations corresponding to their respective IC_50_ values and we have not detected increases in the apoptotic (Annexin V positive population) or the necrotic (PI positive and Annexin V—PI double positive populations) as opposed to the hydrogen peroxide-treated cells used as positive control (Figure 6).

Based on these results, we omitted **Rh-7** from further experiments.

#### 2.2.3 Quinoline-containing complexes are active in cisplatin-resistant cells

**FIGURE 7:**
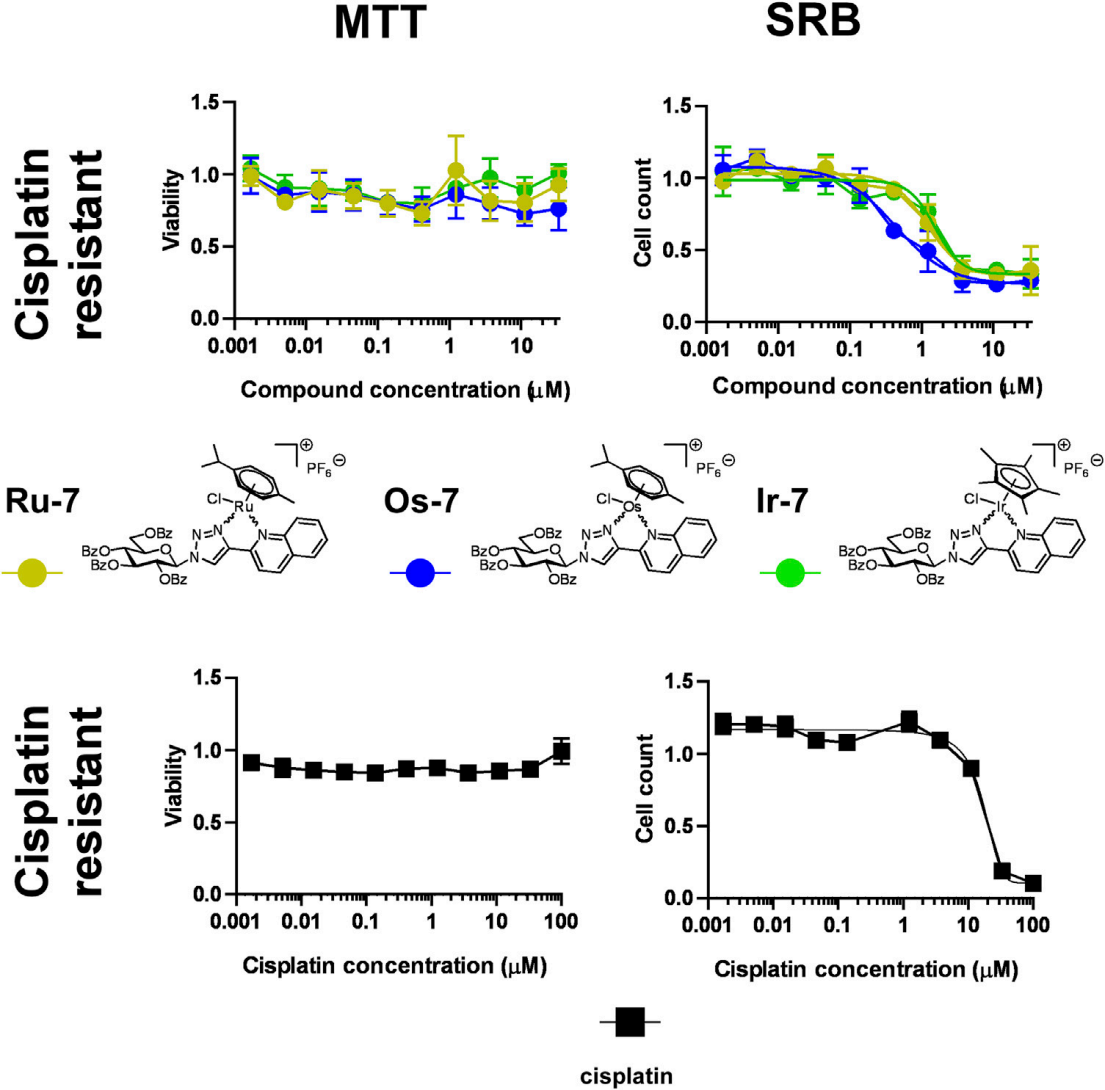
Assessment of quinoline-containing complexes **Ru-7**, **Os-7**, **Ir-7** for cytotoxic and cytostatic activity in cisplatin-resistant A2780 cells. 6 × 10^3^ cisplatin-resistant A2780 cells were plated to 96 well plates. Cells were treated with the compounds in the concentrations indicated for either 4 h for an MTT assay or for 48 h for an SRB assay. Data is represented as average ± SD, from three biological replicates; individual assays were performed in duplicates. Values were normalized for vehicle treated cells, absorbance for vehicle treated cells equals to 1. Each compound was assessed individually. Normality was assessed using the Shapiro-Wilk test. Normality was achieved using the Box-Cox transformation in the case of **Os-7** and **Ir-7** datasets, while **Ru-7** dataset had normal distribution. Statistical significance was assessed using One-way ANOVA test comparing all points to the smallest concentration. For better visibility the *p* values are presented in an excel sheet at https://figshare.com/s/2c942a812caea8869a67. Non-linear regression was performed on the results of the SRB assay datasets.

As noted earlier, one of the major drawbacks of platinum-based drugs is due to cisplatin resistance (Lund et al., 2017; McMullen et al., 2020) that can be likely circumvented when complexes with different central ions and different ligands are used. We tested the three complexes with efficient cytostatic properties (**Ru-7, Os-7** and **Ir-7**) on a cisplatin-resistant A2780 cell line. The complexes did not exert direct toxicity in MTT assays on the cisplatin-resistant cells (Figure 7; Table 2).

Nevertheless, we have observed important differences in cell proliferation. The IC_50_ value of cisplatin was 1.21 µM in our previous study on cisplatin-sensitive A2780 cells (Kacsir et al., 2021). The IC_50_ value increased to 16.47 µM in the cisplatin resistant cell line (13.6 fold increase) in SRB assays. In contrast to that, the IC_50_ value of **Ru-7** (0.8466 µM vs. 1.183 µM, 1.40 fold change) and **Ir-7** (0.891 µM vs. 1.535 µM, 1.72 fold change) increased, although not to the same extent as for cisplatin. Furthermore, the IC_50_ value of **Os-7** (0.5777 µM vs. 0.476 µM) was left technically unchanged when comparing the cisplatin sensitive and cisplatin resistant cell lines (Figure 7; Table 2). Apparently, these complexes are not detoxified strongly in cisplatin resistant cells suggesting that these compounds can be used to overcome cisplatin resistance.

#### 2.2.4 Quinoline-containing complexes are cytostatic in other carcinoma, sarcoma and lymphoma cell lines

**FIGURE 8:**
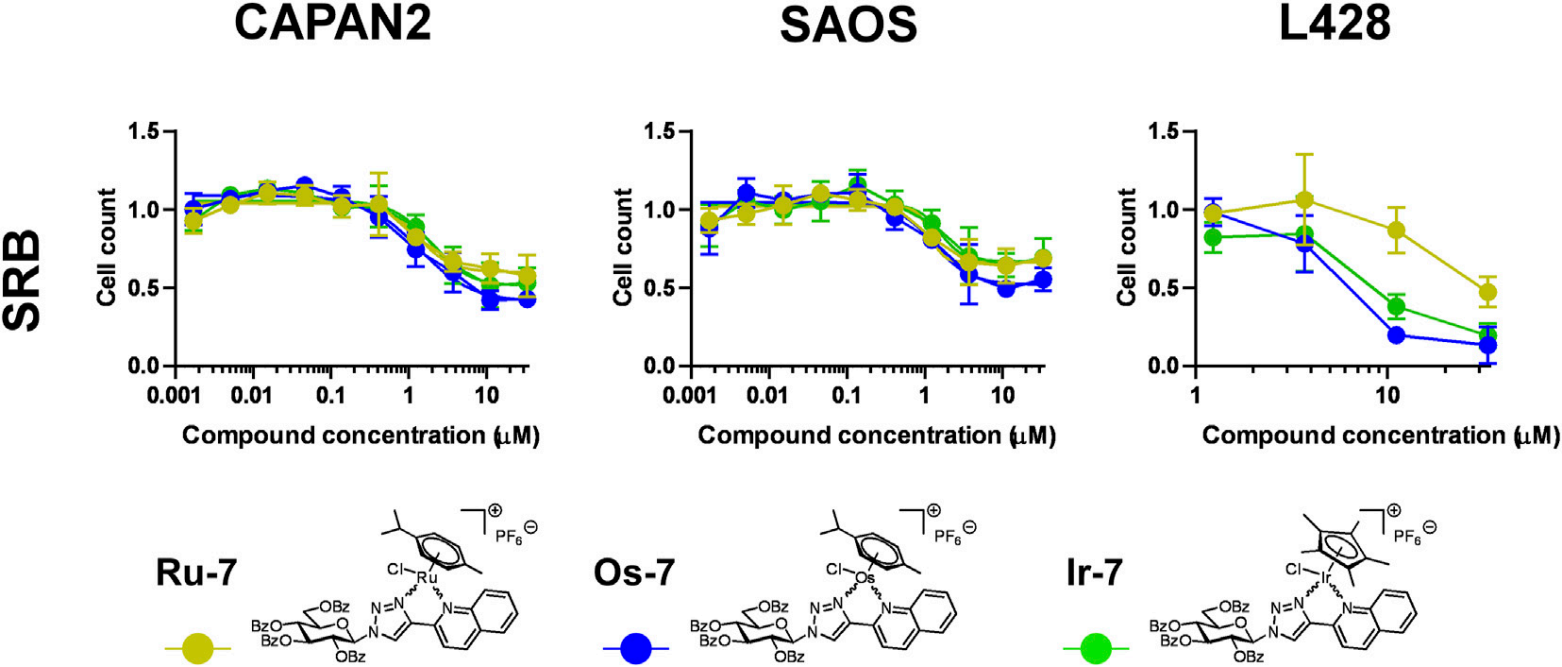
Assessment of quinoline-containing complexes **Ru-7**, **Os-7**, **Ir-7** for cytostatic activity on other cancer cell lines. 3 × 10^3^ Capan2 cells, 2 × 10^3^ Saos cells and 8 × 10^3^ L428 were plated to 96 well plates. Cells were treated with the compounds in the concentrations for 48 h. Then for Capan2 and Saos SRB assay was performed, L428 cells were counted using a Burker chamber. Data is represented as average ± SD, from three biological replicates; individual assays were performed in duplicates. Values were normalized for vehicle treated cells, absorbance for vehicle treated cells equals to 1. Normality was checked using the Shapiro-Wilk test. The Saos **Os-7** dataset was normalized using the Box-Cox method, other datasets had normal distribution. Each complex was individually assessed for statistical significance using One-way ANOVA test followed by Dunnett’s *post hoc* test; all values were compared to the values of the lowest concentration. For better visibility the *p* values are presented in an excel sheet at https://figshare.com/s/2c942a812caea8869a67. Non-linear regression was performed on the Capan2 and Saos datasets.

Next we assessed whether **Ru-7, Os-7, Ir-7** complexes were active on other cancer cell lines. We tested other carcinoma cell lines, Capan2, a pancreatic adenocarcinoma cell line, Saos, an osteosarcoma cell line and L428, a Hodgkin lymphoma cell line to assess a wide array of neoplasias of different origin. All complexes were cytostatic on all the three cell lines (Figure 8; Table 2) with IC_50_ values in the low micromolar range (IC_50_ < 2 µM).

#### 2.2.5 Quinoline-containing complexes induce cytostasis through inducing oxidative stress

**FIGURE 9:**
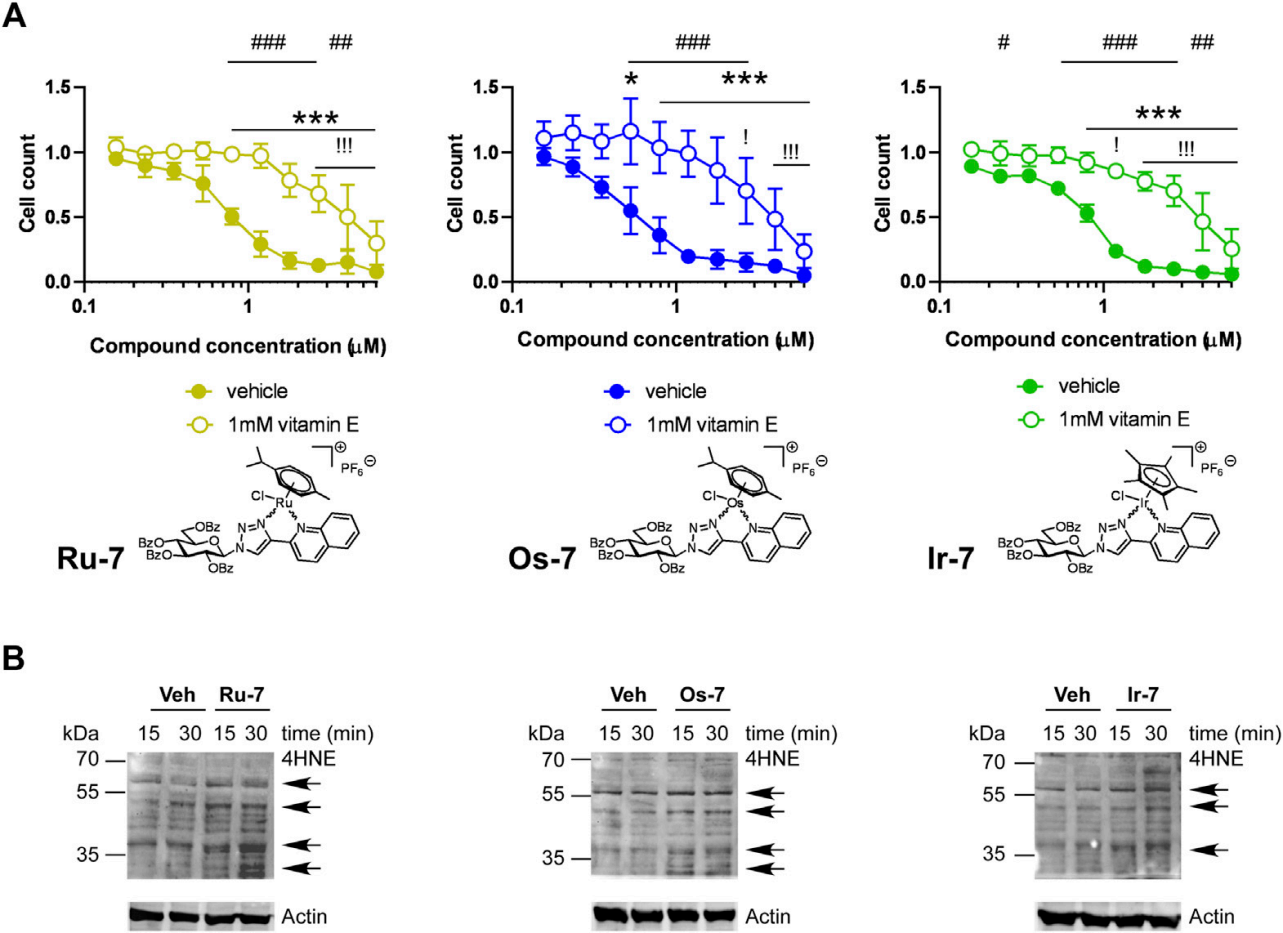
**(A)** Vitamin E, a lipid soluble antioxidant blocks the cytostatic activity of the bioactive complexes. 1.5 × 10^3^ A2780 cells were plated to 96 well plates. Cells were treated with the compounds in the concentrations indicated for 48 h followed by an SRB assay. Data is represented as average ± SD, from four biological replicates; individual assays were performed in duplicates. Values were normalized for vehicle treated cells, absorbance for vehicle treated cells equals to 1. Normality was checked using the D’Agostino-Pearson normality test. The **Ru-7** and the **Os-7** dataset showed normal distribution, while the **Ir-7** dataset was normalized using the Box-Cox normalization method. Statistical significance was assessed using two-way ANOVA test comparing all measurement points. * and *** represent statistical significance at *p* <0.05 and 0.001, respectively, between the lowest concentration and the higher concentrations among non-vitamin E-treated cells. ! and !!! represent statistical significance at *p* <0.05 and 0.001, respectively, between the lowest concentration and the higher concentrations among vitamin E-treated cells. #, ## and ### represent statistical significance at *p* <0.05, *p* <0.01 and 0.001, respectively, between the non-vitamin E-treated vs. vitamin E-treated cells. **(B)** A2780 cells were treated with the indicated complexes (**Ru-7** at 0.846 μM, **Os-7** at 0.578 µM and **Ir-7** at 0.891 µM) for the time indicated. Cells were harvested and lysed. Cellular proteins were separated on a 10% SDS-PAGE gel and were blotted. Blots were probed with the antibodies indicated (top blot with 4HNE antibody, lower blot with actin antibody). Arrows point at band of interest. Experiments were repeated three times and a representative blot is shown. On the blots brightness and contrast were adjusted.

Our previous studies (Kacsir et al., 2021; Kacsir et al., 2022) and studies from other laboratories (Xu et al., 2018; Fernandes, 2019; Bakewell et al., 2020; Mihajlovic et al., 2020) evidenced oxidative stress as a mechanism for cytostasis upon treatment with complexes of ruthenium, osmium or iridium. We assessed whether vitamin E, a lipid soluble antioxidant can block cytostasis induced by the bioactive complexes. Vitamin E treatment increased the IC_50_ values of the complexes in all cases (Figure 9A). This suggests ROS production, similar to the aforementioned prior art (Xu et al., 2018; Fernandes, 2019; Bakewell et al., 2020; Mihajlovic et al., 2020; Kacsir et al., 2021; Kacsir et al., 2022).

To complement the vitamin E experiments we assessed oxidative stress by detecting the formation of 4-hydroxynonenal (4HNE)-modified proteins, a hallmark of oxidative stress (Ayala et al., 2014; Zhang and Forman, 2017). We applied the active **Os-7**, **Ru-7** and **Ir-7** complexes on A2780 cells and sampled cells at early time points, 15 min and 30 min post treatment, to assess 4HNE expression. Treatments were carried out at the IC_50_ concentrations of the respective complexes. We observed a gross induction of 4HNE formation by **Ru-7**, **Os-7** and **Ir-7** with marked increases at molecular weight of ∼70 kDa and ∼60 kDa 15 or 30 min post-treatment (Figure 9B). These bands are similar in size to our previous observation (Kacsir et al., 2021).

#### 2.2.6 Quinoline-containing complexes are bacteriostatic against multiresistant Gram-positive bacteria

In our previous study (Balázs et al., 2022) we showed that ruthenium, osmium, iridium and rhodium complexes, with similar structure to the quinoline-based compounds identified in this study, show bacteriostatic activity against vancomycin-resistant *Enterococcus* (VRE) and methicillin-resistant *Staphylococcus aureus* (MRSA) clinical isolates in low micromolar or submicromolar concentrations, therefore, we tested the bioactive **Ru-7, Os-7** and **Ir-7** complexes for bacteriostatic activity.

**FIGURE 10:**
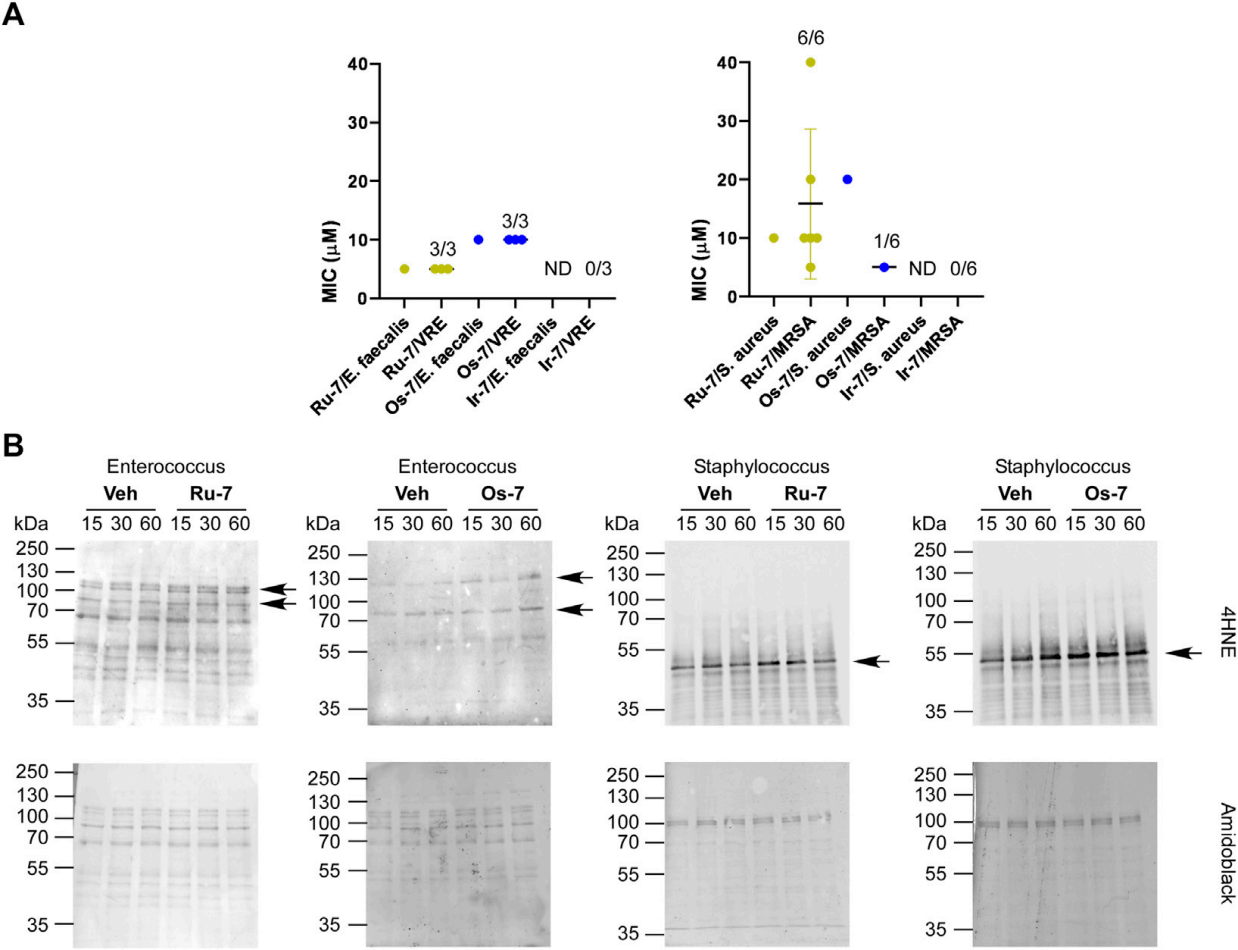
**(A) Ru-7**, **Os-7** and **Ir-7** complexes exert bacteriostatic activity against reference strains and clinical VRE and MRSA isolates. The MIC values of the complexes were determined against the reference strains of *S. aureus* (ATCC29213) and *E. faecalis*. (ATCC29212) and against clinical VRE and MRSA isolates by microdilution assays (repeated at least twice in duplicates) as described in Materials and Methods. The numbers indicate how many isolates were susceptible to the compound out of those tested; i.e., 1/6 stands for 1 isolate was susceptible out of 6 tested. **(B)** The indicated reference strains were treated with the indicated complexes (**Ru-7** at 5 μM, **Os-7** at 10 µM on *Enterococcus faecalis*, **Ru-7** at 10 μM, **Os-7** at 20 µM on *Staphylococcus aureus*) for the time indicated. Cells were harvested and lysed. Cellular proteins was separated on a 10% SDS-PAGE gel and were blotted. Blots were probed with 4HNE antibody (top blot), the lower blot was stained with amido black. Arrows point at band of interest. Experiments were repeated three times and a representative blot is shown. On the blots brightness and contrast were adjusted. Abbreviations: MRSA—methicillin-resistant Staphylococcus aureus, VRE—vancomycin-resistant Enterococcus, ND—not detected, MIC>40 μM, Veh—vehicle.

The **Ru-7** complex had a minimum inhibitory concentration (MIC) of 5 µM against the reference *Enterococcus faecalis* (ATCC29212) strain and a MIC of 10 µM against the *Staphylococcus aureus* (ATCC29213) reference strain. The **Ru-7** complex was bacteriostatic against all tested clinical VRE isolates with a MIC of 5 μM, while inhibited the growth of all clinical MRSA isolates with MIC of 5–40 µM (Figure 10A; Table 3).

**TABLE 3:** The clinical isolates used in the study and the MIC values (in µM) of the complexes against the isolates. VRE—vancomycin-resistant *Enterococcus*, MRSA—methicillin-resistant *Staphylococcus aureus*.

Species and strain identity		**Ru-7**	**Os-7**	**Ir-7**	**Os-2**	**Os-3**	**Os-4**	**Os-5**	**Os-6**	Sample	Year
Reference	*E. faecalis*	5	10	40<	10	5	40	40<	40<		
VRE	25 051	5	10	40<	10	5	40	40<	40<	Nephrostoma	2018
VRE	27 085	5	10	40<	10	5	40	40<	40<	Wound	2018
VRE	25 498	5	10	40<	10	5	40	40<	40<	Rectal swab for screening for multiresistant pathogens	2018
											
Reference	*S. aureus*	10	20	40<	2.5	2.5	40<	40<	40<		
MRSA	24 272	5	5	40<	5	5	40<	40<	40<	Throat	2018
MRSA	24 408	10	40<	40<	5	5	40	40<	40<	Bronchial	2018
MRSA	20 426	10	40<	40<	2.5	5	40<	40<	40<	Blood	2020
MRSA	24 035	40	40<	40<	2.5	2.5	40<	40<	40<	Wound	2018
MRSA	24 328	10	40<	40<	2.5	2.5	40<	40<	40<	Throat	2018
MRSA	24 268	20	40<	40<	5	2.5	40<	40<	40<	Throat	2018

The **Os-7** complex exhibited a MIC of 10 µM against the reference *Enterococcus faecalis* (ATCC29212) strain and a MIC of 20 µM against the *Staphylococcus aureus* (ATCC29213) reference strain. The **Os-7** complex was bacteriostatic against all clinical VRE isolates with a MIC of 10 μM, while inhibited the growth of 1 out of the 6 clinical MRSA isolates with a MIC of 5 µM (Figure 10A; Table 3).

The **Ir-7** complex had no activity either against reference strains or against the clinical isolates (Figure 10A) and was, therefore, omitted from the subsequent experiment.

The quinoline-containing complexes induced reactive oxygen species production that played central role in inducing cytostasis, therefore, we assessed whether **Ru-7**, **Os-7** and **Ir-7** induced 4HNE expression in the *Enterococcus faecalis* and *Streptococcus aureus* reference strains. All treatments were performed in the MIC concentration of the complexes. As the MIC values were higher than the IC_50_ measured on mammalian cells, we opted for longer treatment times (15 min, 30 min and 1 h). **Ru-7** and **Os-7** induced 4HNE expression in a band of ∼120 kDa in the *Enterococcus faecalis* reference strain (Figure 10B). In the reference *Staphylococcus aureus* strain **Ru-7** and **Os-7** induced 4HNE formation in band of ∼ 50 kDa (Figure 10B).

**FIGURE 11:**
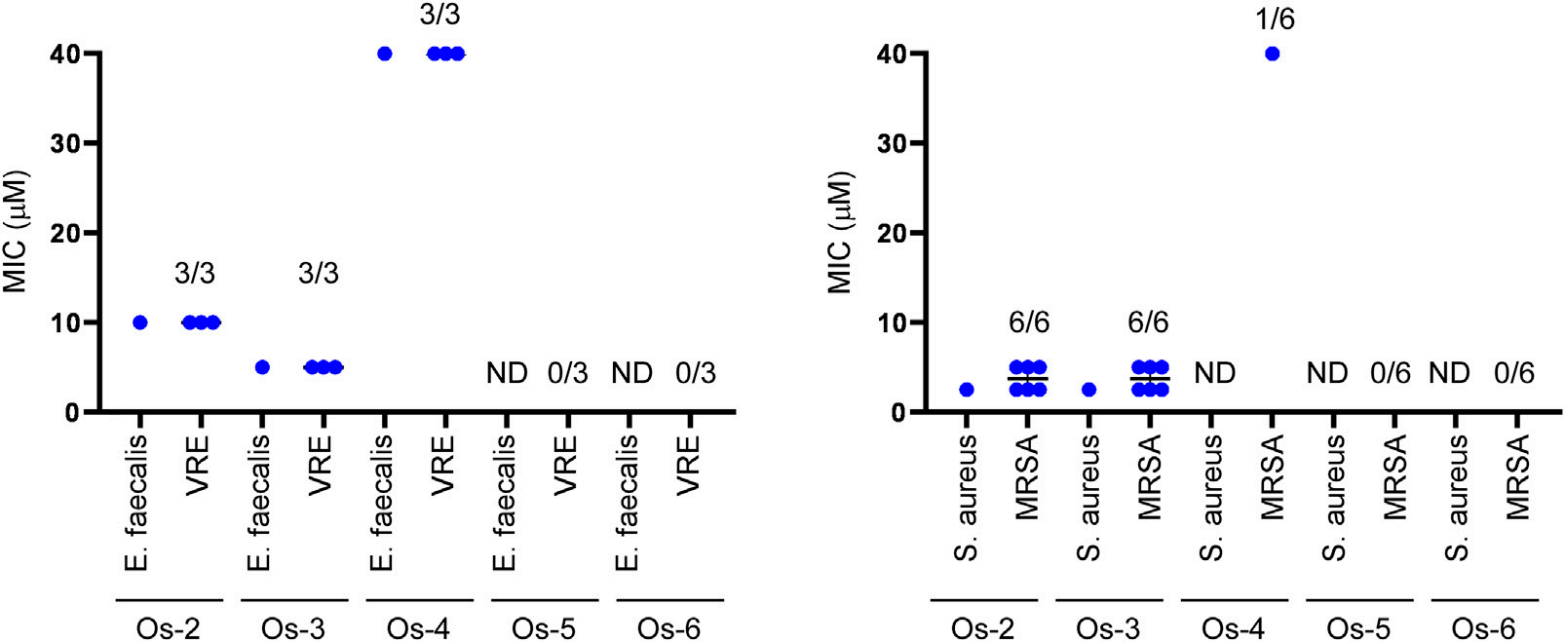
**Os-2** and **Os-3** complexes exert bacteriostatic activity against reference strains and clinical VRE and MRSA isolates. The MIC values of the complexes were determined against the reference strains of *S. aureus* (ATCC29213) and *E. faecalis*. (ATCC29212) and against clinical VRE and MRSA isolates by microdilution assays (repeated at least twice in duplicates) as described in Materials and Methods. The numbers indicate how many isolates were susceptible to the compound out of those tested; i.e., 1/6 stands for 1 isolate was susceptible out of 6 tested. Abbreviations: MRSA—methicillin-resistant *Staphylococcus aureus*, VRE—vancomycin-resistant *Enterococcus*, ND—not detected, MIC>40 µM.

As the **Ru-7**, and **Os-7** complexes exerted bacteriostatic activity we assessed the **Os-2**—**Os-6** compounds in this model system. *Enterococcus faecalis* and *Staphylococcus aureus* reference strains were susceptible to **Os-2** and **Os-3** complexes at MIC values of 2.5—10 µM (Table 3; Figure 11). The reference strain of *Enterococcus faecalis,* but not of *Staphylococcus aureus* was susceptible to the **Os-4** complex with a MIC value of 40 µM (Table 3; Figure 11). Multiresistant VRE and MRSA isolates were also susceptible to **Os-2** and **Os-4** with low micromolar MIC values (Table 3; Figure 11). **Os-4** inhibited the growth of the multiresistant VRE isolates at MIC value of 40 µM (Table 3; Figure 11). **Os-5** and **Os-6** did not inhibit the growth of either of the reference strains or the multiresistant clinical isolates up to 40 µM (Table 3; Figure 11).

## 3 Discussion

In previous studies we identified a set of antineoplastic and antimicrobial carbohydrate-based half sandwich-type complexes with platinum-group metal ions, such as ruthenium(II), osmium(II), iridium(III) and rhodium(III) (Kacsir et al., 2021; Kacsir et al., 2022). A main feature driving the biological activity of these complexes was their apolar nature (logD >+2). The *O*-benzoyl protective groups on the carbohydrate moiety played pivotal role in bringing about the apolar character of the complexes, the removal of the *O*-Bz protective groups or their replacement with *O*-acetyl groups abolished the biological activity of the complexes (Kacsir et al., 2021; Kacsir et al., 2022). These observations were new even in view of reports from other groups, as Hamala and co-workers (Hamala et al., 2020) showed that the length of the carbon chain of *O*-alkanoyl groups on the carbohydrate moiety in 1,4-bis(β-D-glycopyranosyl)tetrazene containing half-sandwich Ru(II) complexes influenced the biological activity (Hamala et al., 2020). The most active compounds contained lipophilic propionyl and butyryl groups. Rapid toxicity, evidenced from the MTT assays, appeared to contribute to the mechanism of cell death. The longer acyl chains improved the antineoplastic activity of the complexes (acetyl < propionyl < butyryl). Another study by Hanif and colleagues (Hanif et al., 2013) evidenced that in RAPTA-analogues, in which the apolar character of the arene moiety was increased by replacing phenyl to biphenyl, the inhibitory activity of the molecules was enhanced. In these compounds cytotoxicity depended on the apolar character of the arene moiety capping the Ru ion of the complex, while the arene ligand had little influence on the hydrolysis behavior.

Hereby, we assessed a set of compounds where the hydroxyl groups of the carbohydrate moiety were protected by aliphatic acyl groups with increasing chain length (C_3_→C_7_–CO). Increasing the length of the acyl chain up to 4 carbons (C_3_–CO) improved the biological activity of the complexes (butyryl < pentanoyl). However, when the acyl chain contained 5 or more carbons (C≥5–CO) the IC_50_ values increased above that of the C_4_-CO-protected complex (pentanoyl < hexanoyl < heptanoyl), despite the continuous increase in the logD value. Likely, increasing length of the acyl chain increased the apolar character of the complexes and thus decreased their solubility in cell medium impairing the cytostatic activity.

Furthermore, in contrast to the complexes with *O*-Bz protective groups, the *O*-alkanoyl-protected complexes exerted toxicity to the cells and their IC_50_ values were inferior to those of the complexes with *O*-Bz-protective groups (Kacsir et al., 2022). Apparently, the nature of the protective groups strongly influences the biological activity of the complexes. Aroyl residues provide superior performance over the alkanoyl groups.

Based on these observations, we prepared another *O*-perbenzoylated 1-glucopyranosyl-1,2,3-triazole ligand, where the pyridine moiety was replaced with a larger quinoline moiety. We synthesized Ru(II), Os(II), Ir(III) and Rh(III) complexes of that free ligand. The IC_50_ values of the quinoline complexes were superior to the previously published complexes (Kacsir et al., 2021; Kacsir et al., 2022). Furthermore, in previous studies pyridine-containing complexes had higher IC_50_ values on ID8 ovarian cancer cells than on A2780 ovarian cancer cells (Kacsir et al., 2021; Kacsir et al., 2022), however, the quinoline-containing complexes had similar submicromolar IC_50_ values on both ovarian cancer cell lines further supporting their superior biological activity. Importantly, none of the complexes was toxic or cytostatic on primary, non-transformed human dermal fibroblasts pointing out the selectivity of these compounds to cancer cells. Selectivity was assessed for structurally unrelated Os(II) and Ru(II) complexes with cinnamic acid-derived ligands that were selective for ovarian cancer cells and had no impact on the proliferation of keratinocytes, fibroblasts (Hildebrandt et al., 2022). Selectivity is not a common feature of Ru(II) or Os(II) complexes, as in another study (Iacopini et al., 2022) glycoconjugated ruthenium(II) arene complexes had no selectivity for non-transformed cells.

In addition to the selectivity of the quinoline-containing complexes towards transformed cancer cells apparently the complexes have widespread cytostatic activity among other carcinomas (as Capan2 cells), sarcomas (as SAOS cells) or hematological malignancies (as L428 cells). These observations are similar to our previous findings with analogous pyridine-containing osmium, ruthenium and iridium complexes (Kacsir et al., 2021; Kacsir et al., 2022). In addition, other ruthenium complexes, with similar structure, were shown to be active on different carcinoma cell lines as MDA-MD-231 and MCF7 breast cancer cells (Hamala et al., 2020; Kacsir et al., 2021), colon cancer (Berger et al., 2008; Hanif et al., 2013; Florindo et al., 2015; Florindo et al., 2016), lung cancer (Hanif et al., 2013), cervical carcinoma (HeLa) cells (Florindo et al., 2014), U251 glioblastoma cells (Kacsir et al., 2021), or Capan2 pancreatic adenocarcinoma cells (Kacsir et al., 2021; Kacsir et al., 2022) apart from ovarian cancer. These observations point towards a wide applicability of the complexes, identified in this study, in anticancer therapy.

Cisplatin resistance is a major drawback for the use of the currently registered platinum-based drugs (Lund et al., 2017; Mukherjea et al., 2020; Yu et al., 2020; Sipos et al., 2021). We provided evidence that the quinoline-containing compounds, we described hereby, had IC_50_ values in cisplatin resistant A2780 cells similar to the control, cisplatin-sensitive A2780 cells. Importantly, complex **Os-7** had the same IC_50_ value on both cell lines. Structurally unrelated Ru(II) and Os(II) complexes (Hildebrandt et al., 2022; Iacopini et al., 2022) can overcome cisplatin resistance in A2780 and SKOV3 cell models, similarly to our compounds.

In addition to the selective cytostatic activity, the quinoline-containing complexes exerted bacteriostatic activity similar to the bacteriostatic activity of platinum or palladium compounds (Quirante et al., 2011; Vieites et al., 2011; Zhang et al., 2011; McCarron et al., 2012; Yuan et al., 2018; Hummell and Kirienko, 2020; Yufanyi et al., 2020; Frei et al., 2021; Mansour, 2021) and ruthenium, osmium, iridium and rhodium complexes with similar structure (Balázs et al., 2022). Interestingly, the **Ir-7** complex had no bacteriostatic activity, although, in our previous report (Balázs et al., 2022) certain iridium complexes proved to be active. Our previous observation was that ruthenium and osmium complexes were more likely to be active than iridium or rhodium complexes (Balázs et al., 2022), however, yet we have no *bona fide* explanation to that phenomenon. The MIC values of the **Ru-7** and **Os-7** complexes were in the low micromolar range similar to their pyridine-containing counterparts against both the VRE and the MRSA isolates (Balázs et al., 2022). VRE isolates were more susceptible to **Ru-7** and **Os-7** than MRSA isolates that aligns well with our previous observation (Balázs et al., 2022). A very recent report showed that dinuclear Ru(II) sandwich complexes with *O*-acetyl protected galactopyranose moieties exhibited nanomolar IC_50_ against *Trypanosoma gondii*, indicating a further potential field of application of the compounds presented here (Holzer et al., 2022).

The complexes with *O*-alkanoyl-protected glucosyl-1,2,3-triazoles also exerted bacteriostatic activity on the reference strains of *Enterococcus faecalis* and *Staphylococcus aureus*, as well as on the multiresistant VRE and MRSA isolates. The complexes with C_3_-CO and the C_4_-CO alkanoyl protective groups were bacteriostatic in the low micromolar range. However, complexes with longer alkanoyl chains were less efficient or proved to be inactive. This observation is similar to the changes in cytostatic activity, where increasing length of the acyl chain increased the apolar character of the complexes and thus decreased their solubility. Importantly, both the alkanoyl-protected and the quinoline complexes were active on multiresistant clinical isolates suggesting that these compounds may represent a novel class of antibiotics.

The cytostatic activity of the complexes was dependent on the generation of reactive oxygen species (ROS). In fact, ROS production was evidenced among platinum group metal complexes (Xu et al., 2018; Fernandes, 2019; Parveen et al., 2019; Bakewell et al., 2020; Mihajlovic et al., 2020; Li et al., 2021a; Kacsir et al., 2021). Importantly, 4HNE signal was induced at an early time point, 15 min after treatment. Furthermore, 4HNE signal was induced on specific bands that were similar to those we observed earlier (Kacsir et al., 2021). A plethora of studies report that ROS production in tumor cells is limited and even minute increases in oxidative stress leads to cytostasis (Xu et al., 2018; Fernandes, 2019; Kovács et al., 2019; Sári et al., 2020a; Bakewell et al., 2020; Sári et al., 2020b; Mihajlovic et al., 2020; Smolková et al., 2020). We have demonstrated previously that complexes with similar structure induce oxidative stress and oxidative stress has central roles in their cytostatic activity (Kacsir et al., 2021; Kacsir et al., 2022). In the case of the quinoline-based complexes, that we report here, vitamin E protected cells against the cytostatic effects of the osmium, ruthenium and iridium complexes that underlines the pivotal role of oxidative stress elicited by the complexes. Of note, vitamin E has a long, apolar phytyl chain; if this phytyl chain was removed the protective capacity was lost in the case of ruthenium complexes of similar structure as the ones we report here (Kacsir et al., 2021). This observation together with the apolar nature of the complexes suggest that the complexes likely target apolar compartments in cells.

In this study we have assessed whether changing apolar parts of platinum-group metal half sandwich complexes with bidentate glycosyl heterocyclic ligands influenced their biological activity. We showed that replacing the *O*-benzoyl protective groups of the carbohydrate moiety to straight chain *O*-acyl groups worsened the cytostatic ability of the complexes and rendered them toxic. The replacement of the pyridine substituent with a quinoline moiety improved the IC_50_ value of the complexes. The complexes were active in a wide variety of carcinoma, sarcoma and lymphoma cell lines. Furthermore, we showed ROS production to play a central role in the biological activity of the complexes. Importantly, the bioactive derivatives were active on platinum-resistant cells suggesting that these complexes may be able to overcome cisplatin resistance *in vivo*. Finally, the bioactive complexes were bacteriostatic on MRSA and VRE clinical isolates with low micromolar MIC values.

## 4 Materials and methods

### 4.1 Syntheses

#### 4.1.1 General methods

The ^1^H and ^13^C NMR spectra of the newly synthesized compounds were recorded with DRX360 (360/90 MHz for ^1^H/^13^C) or DRX400 (400/100 MHz for ^1^H/^13^C) spectrometers (Bruker, Karlsruhe, Germany). Chemical shifts are referenced to Me_4_Si (^1^H-NMR) or to the residual solvent signals (^13^C-NMR). The HRMS data were obtained by using a Bruker maXis II (ESI-HRMS) spectrometer in positive ionization mode. For TLC analysis DC Kieselgel 60 F_254_ plates (Sigma-Aldrich, Saint Louis, MO, United States) were applied, and the spots on the plates were checked under UV light and were developed by gentle heating. For column chromatographic purification Kieselgel 60 (Molar Chemicals, Halásztelek, Hungary, particle size 0.063–0.2 mm) silica gel was applied. Anhydrous pyridine was purchased from VWR Chemicals, while anhydrous CH_2_Cl_2_ and MeOH were freshly prepared before using: CH_2_Cl_2_ was obtained by distillation from P_4_O_10_, while MeOH was distilled over Mg turnings and iodine. The dichloro(η^6^-*p*-cymene)ruthenium(II) dimer (**Ru-dimer**, Strem Chemicals, Newburyport, MA, United States), the dichloro(pentamethylcyclopentadienyl)iridium(III) dimer (**Ir-dimer**, Acros Organics), the dichloro(pentamethylcyclopentadienyl)rhodium(III) dimer (**Rh-dimer**, Alfa Aesar) and TlPF_6_ (Strem Chemicals) are commercially available chemicals purchased from the listed suppliers. The dichloro(η^6^-*p*-cymene)osmium(II) dimer (**Os-dimer**) was prepared according to a literature method (Godó et al., 2012). The 1-(β-D-glucopyranosyl)-4-(pyridin-2-yl)- and -(quinolin-2-yl)-1,2,3-triazoles (**1** and **2**, respectively) were synthesized according to our earlier described procedures (Kacsir et al., 2021).

#### 4.1.2 General procedure I for *O*-peracylation of the 1-(β-D-glucopyranosyl)-4-hetaryl-1,2,3-triazoles

A solution of the appropriate 1-(β-D-glucopyranosyl)-4-hetaryl-1,2,3-triazole (**1** or **2)** in anhydrous pyridine (4 mL/50 mg substrate) was cooled down in an ice bath and the corresponding carboxylic acid chloride (4.8 equiv.) was added under stirring. The reaction mixture was then heated at 60°C and monitored by TLC (1 : 1 CHCl_3_-MeOH and 1 : 2 EtOAc-hexane). If the TLC showed incomplete conversion after one hour, an additional portion of acid chloride (4.8 equiv.) was added to the mixture. After completion of the reaction the pyridine was removed *in vacuo*. The residue was dissolved in CHCl_3_ (30 mL) and extracted with sat. aq. solution of NaHCO_3_ (2 × 30 mL) and with water (35 mL). The separated organic phase was dried over MgSO_4_, filtered and evaporated. The residual crude product was purified by column chromatography.

#### 4.1.3 General procedure II for the preparation of the [(η^6^-*p*-cym)M^II^(N-N)Cl]PF_6_ (M = Ru, Os) and [(η^5^-Cp*)M^III^(N-N)Cl]PF_6_ (M = Ir, Rh) type complexes

The corresponding *O*-peracylated 1-(β-D-glucopyranosyl)-4-hetaryl-1,2,3-triazole (**L-2‒L-7**, 2.0 or 2.1 equiv.), the complex dimer (**Ru-/Os-/Ir-/Rh-dimer**, 1 equiv.) and TlPF_6_ (2 equiv.) were dissolved in a 1 : 1 mixture of anhydrous CH_2_Cl_2_ and MeOH (1–1 mL/10 mg dimer). The reaction mixture was vigorously stirred until the TLC (95 : 5 CHCl_3_-MeOH) showed complete disappearance of the starting dimer complex. After completion of the reaction, the precipitated TlCl was filtered off and the solvents were removed. The residual crude product was purified by crystallization or by column chromatography.

### 4.2 Determination of the distribution coefficients (logD)

The logD values of the newly synthesized complexes **Os-2‒Os-7**, **Ru-7**, **Ir-7** and **Rh-7** were determined according to a procedure described in our previous publications (Kacsir et al., 2021; Kacsir et al., 2022).

### 4.3 Materials

In the cell biology and biochemistry assays all chemicals were from Sigma-Aldrich unless otherwise stated. Cisplatin was purchased from Sigma-Aldrich.

### 4.4 Cell cultures

Cells were cultured under standard cell culture conditions, 37°C, 5% CO_2_, humidified atmosphere.

*A2780* cells were cultured in RMPI 1640 medium supplemented with 10% fetal calf serum, 2 mM glutamine, 1% penicillin-streptomycin.

*ID8* cells were cultured in high glucose DMEM (4.5 g/L glucose) medium supplemented with 4% fetal calf serum, 2 mM glutamine, 1% penicillin-streptomycin, 1% ITS supplement (I3146).

*Capan2* cells were maintained in MEM, 10% fetal bovine serum, 1% Penicillin/Streptomycin, 2 mM Glutamine.

*Human primary dermal fibroblasts* were cultured in low glucose DMEM (1 g/L glucose) medium supplemented with 20% fetal calf serum, 2 mM glutamine, 1% penicillin-streptomycin.

*L428* cells were maintained in RPMI 1640 medium supplemented with 10% fetal calf serum, 2 mM glutamine, 1% penicillin-streptomycin.

*Saos* cells were maintained in DMEM (4.5 g/L glucose) medium supplemented with 10% fetal calf serum, 2 mM glutamine, 1% penicillin-streptomycin.

*Cisplatin resistant A2780* cells were grown in RMPI 1640 medium supplemented with 10% fetal calf serum, 2 mM glutamine, 1% penicillin-streptomycin. Cisplatin resistant cells underwent selection (1 µM cisplatin) once a week for 3 days before plating for any assay.

### 4.5 Bacterial reference strains

We used the reference strains of *Staphylococcus aureus* (ATCC29213), and *Enterococcus faecalis* (ATCC29212) that were purchased from ATCC (Manassas, VA, United States).

### 4.6 Clinical isolates of *S. aureus* and *E. faecium*

We used a set of clinical isloates of *S. aureus and E. faecium* that were collected at the Medical Center of the University of Debrecen (Hungary) between 01. 01. 2018.—31. 12. 2020. The isolates were reported in (Balázs et al., 2022) and are presented in Table 3. The clinical isolates were identified using a Microflex MALDI-TOF mass spectrometer (Bruker, Billerica, MA, United States). Antibiotic susceptibility of the isolates was tested following the European Committee on Antimicrobial Susceptibility Testing (EUCAST, 2021) guidelines valid at the time of collection.

### 4.7 Methylthiazolyldiphenyl-tetrazolium bromide (MTT) reduction assay

MTT reduction assay measures the activity of mitochondrial complex I and can be used to detect toxicity (Virág et al., 1998; Henslee et al., 2016). The assay was performed similar to (Bakondi et al., 2003). Briefly, cells were plated in 96 well plates the day before the assay. Cells were treated with the compounds for 4 h, then MTT was added in 0.5 mg/ml final concentration and cells were incubated at 37°C in a cell incubator. Culture media was removed and the reduced MTT dye was dissolved in dimethyl-sulfoxide (DMSO) and plates were measured in a plate photometer (Thermo Scientific Multiscan GO spectrophotometer, Waltham, MA, United States) at 540 nm. On each plate wells were designed to contain untreated/vehicle-treated cells. In calculations the readings for these wells was considered as 1 and all readings were expressed relative to these values.

### 4.8 Sulforhodamine B (SRB) binding assay

The SRB assay measures total protein content that is proportional to cell number, hence can be used to assess cell proliferation or long-term cytostasis (Skehan et al., 1990). The assay was performed similar to (Kovács et al., 2019). Cells were seeded in 96 well plates the day before the assay. Cells were treated with the compounds for 48 h. Cells were fixed with 10% trichloroacetic acid (TCA). Fixed cells were washed in distilled water 3 times followed by staining with SRB (0.4 m/V% dissolved in 1% acetic acid) for 10 min. Stained cells were washed in 1% acetic acid 5 times; acetic acid was removed and cells were left to dry. Protein-bound SRB was released by adding 100 µl 10 mM Tris base. Plates were measured in a plate photometer (Thermo Scientific Multiscan GO spectrophotometer, Waltham, MA, United States) at 540 nm. On each plate wells were designed to contain vehicle-treated cells. In calculations the readings for these wells was considered as 1 and all readings were expressed relative to these values.

### 4.9 Assessment of cell proliferation on L428 cells

Cells were seeded in 96 well plates the day before the assay. Cells were treated with the compounds for 96 h. Cells were counted using a Burker chamber. On each plate wells were designed to contain untreated cells. In calculations the readings for these wells was considered as 1 and all readings were expressed relative to these values.

### 4.10 Annexin V—Propidium iodide double staining

The proportions of dead cells was assessed using the Annexin V—propidium iodide assay and was measured using flow cytometry using BD FacsCalibur (BD Biosciences, Franklin Lakes, NJ, United States) instrument and the FITC Annexin V/Dead Cell Apoptosis kit (Life Technologies, Eugene, OR, United States) according to the manufacturer’s instructions similar to (Bai et al., 2001). Quadrants were set based on the FITC and PI values observed for the vehicle-treated cells. Ten thousand cells were measured and the percent in the quadrants was used for the subsequent calculation.

### 4.11 Broth microdilution

Microdilution experiments were performed according to the standards of EUCAST (EUCAST, 2021). The bacterial isolates to be tested were grown on Mueller-Hinton agar plates. Inoculum density of bacteria was set at 5.0 × 10^5^ CFU/mL in microtiter plates in a final volume of 200 µL Mueller-Hinton broth. Tested concentration range was 0.08–40 µM (10 concentrations, two-fold serial dilutions), drug-free growth control and inoculum-free negative control were included. The inoculated plates were incubated for 24 h at 37°C then were assessed visually. Minimum inhibitory concentration (MIC) was defined as the lowest concentration with 50% ≤ inhibitory effect compared to the growth control. All experiments were performed at least twice in duplicates.

### 4.12 SDS-polyacrylamide gel electrophoresis and western blot

SDS-polyacrylamide gel electrophoresis and Western blot was performed as in (Márton et al., 2018) using an antibody against 4HNE (Abcam (ab46545) 1:1000) that was detected using a corresponding peroxidase-conjugated secondary antibody (1:10000, Cell Signaling Technology, Inc., Beverly, MA, United States). Protein loading in the A2780 lysates was controlled using a peroxidase-conjugated anti-actin antibody (Sigma, 1:1000). Blots were developed using the Western Pico ECL kit (Thermo Scientific). Equal loading in the bacterial lysates was controlled by amido black coloration of the blots. ECL and amido black pictures were captured using the ChemiDoc Touch Imaging System (Bio-Rad Laboratories, Inc., Hercules, California, United States) and the pictures were evaluated using the Image Lab software (Bio-Rad).

### 4.13 Statistical analysis

Statistical analysis was performed using 8.0.1 version of Graphpad Prism. Values were tested for normal distribution using the D’Agostino and Pearson normality test. When necessary, values were log normalized or were normalized using the Box-Cox normalization method (Box and Cox, 1964) as indicated in the figure captions. The following statistical test, *post hoc* test and the level of significance is indicated in the figure captions. Non-linear regression was performed using the built-in “[Inhibitor] vs. response—Variable slope (four parameters), least square fit” utility of Graphpad that yielded IC_50_ and Hill slope values.

## Data Availability

The datasets presented in the study can be found in online repositories. This data can be found here: https://figshare.com/s/2c942a812caea8869a67.

## References

[B1] Ayala A. Muñoz M. F. Argüelles S. (2014). Lipid peroxidation: production, metabolism, and signaling mechanisms of malondialdehyde and 4-hydroxy-2-nonenal. Oxid. Med. Cell Longev. 2014, 1–31. 10.1155/2014/360438 PMC4066722 24999379

[B2] Bai P. Bakondi E. Szabó E. Gergely P. Szabó C. Virág L. (2001). Partial protection by poly(ADP-ribose) polymerase inhibitors from nitroxyl-induced cytotoxity in thymocytes. Free Radic. Biol. Med. 31, 1616–1623. 10.1016/s0891-5849(01)00756-0 11744336

[B3] Bakewell S. Conde I. Fallah Y. McCoy M. Jin L. Shajahan-Haq A. N. (2020). Inhibition of DNA repair pathways and induction of ROS are potential mechanisms of action of the small molecule inhibitor BOLD-100 in breast cancer. Cancers (Basel) 12, 2647. 10.3390/cancers12092647 32947941 PMC7563761

[B4] Bakondi E. Gönczi M. Szabó E. Bai P. Pacher P. Gergely P. (2003). Role of intracellular calcium mobilization and cell-density-dependent signaling in oxidative-stress-induced cytotoxicity in HaCaT keratinocytes. J. Invest. Dermatol. 121, 88–95. 10.1046/j.1523-1747.2003.12329.x 12839568

[B5] Balázs B. Tóth Z. Kacsir I. Sipos A. Buglyó P. Somsák L. (2022). Targeting multiresistant Gram-positive bacteria by ruthenium, osmium, iridium and rhodium half-sandwich type complexes with bidentate monosaccharide ligands. Front. Chem. 10, 868234. 10.3389/fchem.2022.868234 35494644 PMC9039051

[B6] Berger I. Hanif M. Nazarov A. A. Hartinger C. G. John R. O. Kuznetsov M. L. (2008). *In vitro* anticancer activity and biologically relevant metabolization of organometallic ruthenium complexes with carbohydrate-based ligands. Chemistry—A Eur. J. 14, 9046–9057. 10.1002/chem.200801032 18688905

[B7] Bononi G. Iacopini D. Cicio G. Di Pietro S. Granchi C. Di Bussolo V. (2020). Glycoconjugated metal complexes as cancer diagnostic and therapeutic agents. Chemmedchem 16, 30–64. 10.1002/cmdc.202000456 32735702

[B8] Box G. E. P. Cox D. R. (1964). An analysis of transformations. J. R. Stat. Soc. B 26, 211–243. 10.1111/j.2517-6161.1964.tb00553.x

[B9] Brown A. Kumar S. Tchounwou P. B. (2019). Cisplatin-based chemotherapy of human cancers. J. Cancer Sci. Ther. 11, 97. 32148661 PMC7059781

[B10] Burris H. A. Bakewell S. Bendell J. C. Infante J. Jones S. F. Spigel D. R. (2016). Safety and activity of IT-139, a ruthenium-based compound, in patients with advanced solid tumours: a first-in-human, open-label, dose-escalation phase I study with expansion cohort. ESMO Open 1, e000154. 10.1136/esmoopen-2016-000154 28848672 PMC5548977

[B11] De Camargo M. S. De Grandis R. A. Da Silva M. M. Da Silva P. B. Santoni M. M. Eismann C. E. (2019). Determination of *in vitro* absorption in Caco-2 monolayers of anticancer Ru(II)-based complexes acting as dual human topoisomerase and PARP inhibitors. Biometals 32, 89–100. 10.1007/s10534-018-0160-0 30506342

[B12] EUCAST (2021). MIC determination of non-fastidious and fastidious organisms. [Online]. Available at: https://www.eucast.org/ast_of_bacteria/mic_determination/?no_cache=1 (Accessed 11 04, 2021).

[B13] Fernandes A. C. (2019). Synthesis, biological activity and medicinal applications of ruthenium complexes containing carbohydrate ligands. Curr. Med. Chem. 26, 6412–6437. 10.2174/0929867326666190124124350 30678616

[B14] Fetoni A. R. Paciello F. Mezzogori D. Rolesi R. Eramo S. L. Paludetti G. (2015). Molecular targets for anticancer redox chemotherapy and cisplatin-induced ototoxicity: the role of curcumin on pSTAT3 and nrf-2 signalling. Br. J. Cancer 113, 1434–1444. 10.1038/bjc.2015.359 26469832 PMC4815880

[B15] Florindo P. Marques I. J. Nunes C. D. Fernandes A. C. (2014). Synthesis, characterization and cytotoxicity of cyclopentadienyl ruthenium(II) complexes containing carbohydrate-derived ligands. J. Organomet. Chem. 760, 240–247. 10.1016/j.jorganchem.2013.09.004

[B16] Florindo P. R. Pereira D. M. Borralho P. M. Rodrigues C. M. P. Piedade M. F. M. Fernandes A. C. (2015). Cyclopentadienyl-Ruthenium(II) and iron(II) organometallic compounds with carbohydrate derivative ligands as good colorectal anticancer agents. J. Med. Chem. 58, 4339–4347. 10.1021/acs.jmedchem.5b00403 25923600

[B17] Florindo P. R. Pereira D. M. Borralho P. M. Costa P. J. Piedade M. F. M. Rodrigues C. M. P. (2016). New [(η^5^-C_5_H_5_)Ru(N–N)(PPh_3_)] [PF_6_] compounds: colon anticancer activity and GLUT-mediated cellular uptake of carbohydrate-appended complexes. Dalton Trans. 45, 11926–11930. 10.1039/c6dt01571a 27216868

[B18] Frei A. Ramu S. Lowe G. J. Dinh H. Semenec L. Elliott A. G. (2021). Platinum cyclooctadiene complexes with activity against gram-positive bacteria. ChemMedChem 16, 3165–3171. 10.1002/cmdc.202100157 34018686 PMC8596843

[B19] Gano L. Pinheiro T. Matos A. P. Tortosa F. Jorge T. F. Gonçalves M. S. (2019). Antitumour and toxicity evaluation of a Ru(II)-Cyclopentadienyl complex in a prostate cancer model by imaging tools. Anti-Cancer Agents Med. Chem. 19, 1262–1275. 10.2174/1871520619666190318152726 30887931

[B20] Gesztelyi R. Zsuga J. Kemény-Beke A. Varga B. Juhász B. Tósaki A. (2012). The Hill equation and the origin of quantitative pharmacology. Archive Hist. Exact Sci. 66, 427–438. 10.1007/s00407-012-0098-5

[B21] Gichumbi J. M. Friedrich H. B. (2018). Half-sandwich complexes of platinum group metals (Ir, Rh, Ru and Os) and some recent biological and catalytic applications. J. Organomet. Chem. 866, 123–143. 10.1016/j.jorganchem.2018.04.021

[B22] Godó A. J. Bényei A. C. Duff B. Egan D. A. Buglyó P. (2012). Synthesis and X-ray diffraction structures of novel half-sandwich Os(II)-and Ru(II)-hydroxamate complexes. RSC Adv. 2, 1486–1495. 10.1039/c1ra00998b

[B23] Hamala V. Martišová A. Červenková Šťastná L. Karban J. Dančo A. Šimarek A. (2020). Ruthenium tetrazene complexes bearing glucose moieties on their periphery: Synthesis, characterization, and *in vitro* cytotoxicity. Appl. Organomet. Chem. 34, e5896. 10.1002/aoc.5896

[B24] Hanif M. Meier S. Nazarov A. Risse J. Legin A. Casini A. (2013). Influence of the π-coordinated arene on the anticancer activity of ruthenium(II) carbohydrate organometallic complexes. Front. Chem. 1, 27. 10.3389/fchem.2013.00027 24790955 PMC3982558

[B25] Hanif M. Babak M. V. Hartinger C. G. (2014). Development of anticancer agents: wizardry with osmium. Drug Discov. Today 19, 1640–1648. 10.1016/j.drudis.2014.06.016 24955838

[B26] Hartinger C. G. Nazarov A. A. Ashraf S. M. Dyson P. J. Keppler B. K. (2008). Carbohydrate-metal complexes and their potential as anticancer agents. Curr. Med. Chem. 15, 2574–2591. 10.2174/092986708785908978 18855680

[B27] Hartinger C. G. Phillips A. D. Nazarov A. A. (2011). Polynuclear ruthenium, osmium and gold complexes. The quest for innovative anticancer chemotherapeutics. Curr. Top. Med. Chem. 11, 2688–2702. 10.2174/156802611798040769 22039871

[B28] Henslee E. A. Torcal Serrano R. M. Labeed F. H. Jabr R. I. Fry C. H. Hughes M. P. (2016). Accurate quantification of apoptosis progression and toxicity using a dielectrophoretic approach. Analyst 141, 6408–6415. 10.1039/c6an01596d 27774532

[B29] Hildebrandt J. Häfner N. Kritsch D. Görls H. Dürst M. Runnebaum I. B. (2022). Highly cytotoxic osmium(II) compounds and their ruthenium(II) analogues targeting ovarian carcinoma cell lines and evading cisplatin resistance mechanisms. Int. J. Mol. Sci. 23, 4976. 10.3390/ijms23094976 35563367 PMC9102668

[B30] Holzer I. Desiatkina O. Anghel N. Johns S. K. Boubaker G. Hemphill A. (2022). Trithiolato-bridged dinuclear arene ruthenium(ll)- glycoconjugates: Synthesis and antiparasitic activity. Cambridge: Cambridge Open Engage. ChemRxiv.

[B31] Hummell N. A. Kirienko N. V. (2020). Repurposing bioactive compounds for treating multidrug-resistant pathogens. J. Med. Microbiol. 69, 881–894. 10.1099/jmm.0.001172 32163353 PMC7363280

[B32] Iacopini D. Vančo J. Di Pietro S. Bordoni V. Zacchini S. Marchetti F. (2022). New glycoconjugation strategies for ruthenium(II) arene complexes via phosphane ligands and assessment of their antiproliferative activity. Bioorg. Chem. 126, 105901. 10.1016/j.bioorg.2022.105901 35671646

[B33] Kacsir I. Sipos A. Ujlaki G. Buglyó P. Somsák L. Bai P. (2021). Ruthenium half-sandwich type complexes with bidentate monosaccharide ligands show antineoplastic activity in ovarian cancer cell models through reactive oxygen species production. Int. J. Mol. Sci. 22, 10454. 10.3390/ijms221910454 34638791 PMC8508960

[B34] Kacsir I. Sipos A. Bényei A. Janka E. Buglyó P. Somsák L. (2022). Reactive oxygen species production is responsible for antineoplastic activity of osmium, ruthenium, iridium and rhodium half-sandwich type complexes with bidentate glycosyl hetero-cyclic ligands in various cancer cell models. Int. J. Mol. Sci. 23, 813. 10.3390/ijms23020813 35054999 PMC8776094

[B35] Kenny R. G. Marmion C. J. (2019). Toward multi-targeted platinum and ruthenium drugs-A new paradigm in cancer drug treatment regimens? Chem. Rev. 119, 1058–1137. 10.1021/acs.chemrev.8b00271 30640441

[B36] Konkankit C. C. Marker S. C. Knopf K. M. Wilson J. J. (2018). Anticancer activity of complexes of the third row transition metals, rhenium, osmium, and iridium. Dalton Trans. 47, 9934–9974. 10.1039/c8dt01858h 29904760

[B37] Kovács P. Csonka T. Kovács T. Sári Z. Ujlaki G. Sipos A. (2019). Lithocholic acid, a metabolite of the microbiome, increases oxidative stress in breast cancer. Cancers (Basel) 11, 1255. 10.3390/cancers11091255 31461945 PMC6769524

[B38] Kulkarni G. S. Lilge L. Nesbitt M. Dumoulin-White R. J. Mandel A. Jewett M. a. S. (2022). A phase 1b clinical study of intravesical photodynamic therapy in patients with Bacillus calmette-guérin-unresponsive non-muscle-invasive bladder cancer. Eur. Urol. Open Sci. 41, 105–111. 10.1016/j.euros.2022.04.015 35813250 PMC9257636

[B39] Leijen S. Burgers S. A. Baas P. Pluim D. Tibben M. Van Werkhoven E. (2015). Phase I/II study with ruthenium compound NAMI-A and gemcitabine in patients with non-small cell lung cancer after first line therapy. Investig. New Drugs 33, 201–214. 10.1007/s10637-014-0179-1 25344453

[B40] Leung C. H. Zhong H. J. Chan D. S. H. Ma D. L. (2013). Bioactive iridium and rhodium complexes as therapeutic agents. Coord. Chem. Rev. 257, 1764–1776. 10.1016/j.ccr.2013.01.034

[B41] Li G. Liu H. Feng R. Kang T. S. Wang W. Ko C. N. (2021). A bioactive ligand-conjugated iridium(III) metal-based complex as a Keap1-Nrf2 protein-protein interaction inhibitor against acetaminophen-induced acute liver injury. Redox Biol. 48, 102129. 10.1016/j.redox.2021.102129 34526248 PMC8710994

[B42] Li Y. Liu B. Shi H. Wang Y. Sun Q. Zhang Q. (2021). Metal complexes against breast cancer stem cells. Dalton Trans. 50, 14498–14512. 10.1039/d1dt02909f 34591055

[B43] Liu Z. Sadler P. J. (2014). Organoiridium complexes: anticancer agents and catalysts. Accounts Chem. Res. 47, 1174–1185. 10.1021/ar400266c PMC3994614 24555658

[B44] Liu J. Lai H. Xiong Z. Chen B. Chen T. (2019). Functionalization and cancer-targeting design of ruthenium complexes for precise cancer therapy. Chem. Commun. 55, 9904–9914. 10.1039/c9cc04098f 31360938

[B45] Lund R. J. Huhtinen K. Salmi J. Rantala J. Nguyen E. V. Moulder R. (2017). DNA methylation and transcriptome changes associated with cisplatin resistance in ovarian cancer. Sci. Rep. 7, 1469. 10.1038/s41598-017-01624-4 28473707 PMC5431431

[B46] Máliková K. Masaryk L. Štarha P. (2021). Anticancer half-sandwich rhodium(III) complexes. Inorganics 9, 26. 10.3390/inorganics9040026

[B47] Mansour A. M. (2021). Pd(ii) and Pt(ii) complexes of tridentate ligands with selective toxicity against Cryptococcus neoformans and Candida albicans. RSC Adv. 11, 39748–39757. 10.1039/d1ra06559a 35494132 PMC9044551

[B48] Márton J. Fodor T. Nagy L. Vida A. Kis G. Brunyánszki A. (2018). PARP10 (ARTD10) modulates mitochondrial function. PLoS One 13, e0187789. 10.1371/journal.pone.0187789 29293500 PMC5749700

[B49] Mccarron A. J. Armstrong C. Glynn G. Millar B. C. Rooney P. J. Goldsmith C. E. (2012). Antibacterial effects on acinetobacter species of commonly employed antineoplastic agents used in the treatment of haematological malignancies: an *in vitro* laboratory evaluation. Br. J. Biomed. Sci. 69, 14–17. 10.1080/09674845.2012.11669916 22558799

[B50] Mcmullen M. Madariaga A. Lheureux S. (2020). New approaches for targeting platinum-resistant ovarian cancer. Semin. Cancer Biol. 77, 167–181. 10.1016/j.semcancer.2020.08.013 32871277

[B51] Meier-Menches S. M. Gerner C. Berger W. Hartinger C. G. Keppler B. K. (2018). Structure–activity relationships for ruthenium and osmium anticancer agents – towards clinical development. Chem. Soc. Rev. 47, 909–928. 10.1039/c7cs00332c 29170783

[B52] MelChart M. Sadler P. J. (2005). “Ruthenium arene anticancer complexes,” in Bioorganometallics, 39–64. 10.1039/b508531b 16193110

[B53] Mello-Andrade F. Cardoso C. G. Silva C. R. E. Chen-Chen L. Melo-Reis P. R. Lima A. P. (2018). Acute toxic effects of ruthenium (II)/amino acid/diphosphine complexes on Swiss mice and zebrafish embryos. Biomed. Pharmacother. 107, 1082–1092. 10.1016/j.biopha.2018.08.051 30257320

[B54] Mihajlovic K. Milosavljevic I. Jeremic J. Savic M. Sretenovic J. Srejovic I. M. (2020). Redox and apoptotic potential of novel ruthenium complexes in rat blood and heart. Can. J. Physiology Pharmacol. 99, 207–217. 10.1139/cjpp-2020-0349 32976727

[B55] Mukherjea D. Dhukhwa A. Sapra A. Bhandari P. Woolford K. Franke J. (2020). Strategies to reduce the risk of platinum containing antineoplastic drug-induced ototoxicity. Expert Opin. Drug Metab. Toxicol. 16, 965–982. 10.1080/17425255.2020.1806235 32757852 PMC7606369

[B56] Nabiyeva T. Marschner C. Blom B. (2020). Synthesis, structure and anti-cancer activity of osmium complexes bearing pi-bound arene substituents and phosphane Co-ligands: A review. Eur. J. Med. Chem. 201, 112483. 10.1016/j.ejmech.2020.112483 32592914

[B57] Parveen S. Hanif M. Leung E. Tong K. K. H. Yang A. Astin J. (2019). Anticancer organorhodium and -iridium complexes with low toxicity *in vivo* but high potency *in vitro*: DNA damage, reactive oxygen species formation, and haemolytic activity. Chem. Commun. 55, 12016–12019. 10.1039/c9cc03822a 31498360

[B58] Quirante J. Ruiz D. Gonzalez A. López C. Cascante M. Cortés R. (2011). Platinum(II) and palladium(II) complexes with (N, N') and (C, N, N')- ligands derived from pyrazole as anticancer and antimalarial agents: synthesis, characterization and *in vitro* activities. J. Inorg. Biochem. 105, 1720–1728. 10.1016/j.jinorgbio.2011.09.021 22104300

[B59] Sári Z. Mikó E. Kovács T. Boratkó A. Ujlaki G. Jankó L. (2020a). Indoxylsulfate, a metabolite of the microbiome, has cytostatic effects in breast cancer via activation of AHR and PXR receptors and induction of oxidative stress. Cancers (Basel) 12, 2915. 10.3390/cancers12102915 33050543 PMC7599465

[B60] Sári Z. Mikó E. Kovács T. Jankó L. Csonka T. Sebő E. (2020b). Indolepropionic acid, a metabolite of the microbiome, has cytostatic properties in breast cancer by activating AHR and PXR receptors and inducing oxidative stress. Cancers (Basel) 12, 2411. 10.3390/cancers12092411 32854297 PMC7565149

[B61] Sipos A. Ujlaki G. Mikó E. Maka E. Szabó J. Uray K. (2021). The role of the microbiome in ovarian cancer: mechanistic insights into oncobiosis and to bacterial metabolite signaling. Mol. Med. 27, 33. 10.1186/s10020-021-00295-2 33794773 PMC8017782

[B62] Skehan P. Storeng R. Scudiero D. Monks A. Mcmahon J. Vistica D. (1990). New colorimetric cytotoxicity assay for anticancer-drug screening. J. Natl. Cancer Inst. 82, 1107–1112. 10.1093/jnci/82.13.1107 2359136

[B63] Smolková K. Mikó E. Kovács T. Leguina-Ruzzi A. Sipos A. Bai P. (2020). Nuclear factor erythroid 2-related factor 2 in regulating cancer metabolism. Antioxid. Redox Signal. 33, 966–997. 10.1089/ars.2020.8024 31989830 PMC7533893

[B64] Štarha P. Trávníček Z. (2019). Non-platinum complexes containing releasable biologically active ligands. Coord. Chem. Rev. 395, 130–145. 10.1016/j.ccr.2019.06.001

[B65] Vieites M. Smircich P. Pagano M. Otero L. Fischer F. L. Terenzi H. (2011). DNA as molecular target of analogous palladium and platinum anti-trypanosoma cruzi compounds: a comparative study. J. Inorg. Biochem. 105, 1704–1711. 10.1016/j.jinorgbio.2011.07.018 22142771

[B66] Virág L. Salzman A. L. Szabó C. (1998). Poly(ADP-ribose) synthetase activation mediates mitochondrial injury during oxidant-induced cell death. J. Immunol. 161, 3753–3759. 10.4049/jimmunol.161.7.3753 9759901

[B67] Xu Z. Kong D. He X. Guo L. Ge X. Liu X. (2018). Mitochondria-targeted half-sandwich rutheniumII diimine complexes: anticancer and antimetastasis via ROS-mediated signalling. Inorg. Chem. Front. 5, 2100–2105. 10.1039/c8qi00476e

[B68] Yu C. Wang Z. Sun Z. Zhang L. Zhang W. Xu Y. (2020). Platinum-based combination therapy: Molecular rationale, current clinical uses, and future perspectives. J. Med. Chem. 63, 13397–13412. 10.1021/acs.jmedchem.0c00950 32813515

[B69] Yuan M. Chua S. L. Liu Y. Drautz-Moses D. I. Yam J. K. H. Aung T. T. (2018). Repurposing the anticancer drug cisplatin with the aim of developing novel *Pseudomonas aeruginosa* infection control agents. Beilstein J. Org. Chem. 14, 3059–3069. 10.3762/bjoc.14.284 30591828 PMC6296412

[B70] Yufanyi D. M. Abbo H. S. Titinchi S. J. J. Neville T. (2020). Platinum(II) and ruthenium(II) complexes in medicine: Antimycobacterial and anti-HIV activities. Coord. Chem. Rev. 414, 213285. 10.1016/j.ccr.2020.213285

[B71] Yusoh N. A. Ahmad H. Gill M. R. (2020). Combining PARP inhibition with platinum, ruthenium or gold complexes for cancer therapy. ChemMedChem 15, 2121–2135. 10.1002/cmdc.202000391 32812709 PMC7754470

[B72] Zeng L. Gupta P. Chen Y. Wang E. Ji L. Chao H. (2017). The development of anticancer ruthenium(II) complexes: from single molecule compounds to nanomaterials. Chem. Soc. Rev. 46, 5771–5804. 10.1039/c7cs00195a 28654103 PMC5624840

[B73] Zhang H. Forman H. J. (2017). 4-hydroxynonenal-mediated signaling and aging. Free Radic. Biol. Med. 111, 219–225. 10.1016/j.freeradbiomed.2016.11.032 27876535 PMC5438786

[B74] Zhang L. Zheng Y. Callahan B. Belfort M. Liu Y. (2011). Cisplatin inhibits protein splicing, suggesting inteins as therapeutic targets in mycobacteria. J. Biol. Chem. 286, 1277–1282. 10.1074/jbc.m110.171124 21059649 PMC3020735

