# Supplementary material for: Half sandwich-type osmium, ruthenium, iridium and rhodium complexes with bidentate glycosyl heterocyclic ligands induce cytostasis in platinum-resistant ovarian cancer cells and bacteriostasis in Gram-positive multiresistant bacteria
Source: Front Chem. 2023 Jan 30;11:1086267. doi: 10.3389/fchem.2023.1086267 (PMC9923724; doi:10.3389/fchem.2023.1086267)
Supplement: Supplementary file 1 [file DataSheet1.PDF]

frontiers

Supplementary Material

Table of contents

1. Syntheses 2

1.1 General methods 2

1.2 General procedure I for *O*-peracylation of the 1-(β-D-glucopyranosyl)-4-hetaryl-1,2,3-triazoles....................2

1.3 General procedure II for the preparation of the [(η^6-*p*-cym)M^{II}(N-N)Cl]PF_6 (M = Ru, Os) and [(η^5-Cp*)M^{III}(N-N)Cl]PF_6 (M = Ir, Rh) type complexes 2

1.4 Syntheses and characterization of the new compounds....................3

1-(2',3',4',6'-Tetra-*O*-butanoyl-β-D-glucopyranosyl)-4-(pyridin-2-yl)-1,2,3-triazole (**L-2**) 3

1-(2',3',4',6'-Tetra-*O*-pentanoyl-β-D-glucopyranosyl)-4-(pyridin-2-yl)-1,2,3-triazole (**L-3**).......3

1-(2',3',4',6'-Tetra-*O*-hexanoyl-β-D-glucopyranosyl)-4-(pyridin-2-yl)-1,2,3-triazole (**L-4**)........3

1-(2',3',4',6'-Tetra-*O*-heptanoyl-β-D-glucopyranosyl)-4-(pyridin-2-yl)-1,2,3-triazole (**L-5**).......4

1-(2',3',4',6'-Tetra-*O*-octanoyl-β-D-glucopyranosyl)-4-(pyridin-2-yl)-1,2,3-triazole (**L-6**).........4

1-(2',3',4',6'-Tetra-*O*-benzoyl-β-D-glucopyranosyl)-4-(quinolin-2-yl)-1,2,3-triazole (**L-7**) 4

Complex **Os-2** 5

Complex **Os-3** 5

Complex **Os-4** 5

Complex **Os-5** 6

Complex **Os-6** 6

Complex **Ru-7**....................7

Complex **Os-7** 7

Complex **Ir-7**....................8

Complex **Rh-7**....................8

2. Copies of ^{1}H and ^{13}C NMR spectra....................10

3. Table S1. Distribution coefficient of the synthesized complexes (logD)....................25

5. References 27

1. Syntheses

1.1 General methods

The ^{1}H and ^{13}C NMR spectra of the newly synthesized compounds were recorded with DRX360 (360/90 MHz for $^{1}H/^{13}C$) or DRX400 (400/100 MHz for $^{1}H/^{13}C$) spectrometers (Bruker, Karlsruhe, Germany). Chemical shifts are referenced to Me_4Si (^{1}H-NMR) or to the residual solvent signals (^{13}C-NMR). The HRMS data were obtained by using a Bruker maXis II (ESI-HRMS) spectrometer in positive ionization mode. For TLC analysis DC Kieselgel 60 F_{254} plates (Sigma-Aldrich, Saint Louis, MO, USA) were applied, and the spots on the plates were checked under UV light and were developed by gentle heating. For column chromatographic purification Kieselgel 60 (Molar Chemicals, Halásztelek, Hungary, particle size 0.063–0.2 mm) silica gel was applied. Anhydrous pyridine was purchased from VWR Chemicals, while anhydrous CH_2Cl_2 and MeOH were freshly prepared before using: CH_2Cl_2 was obtained by distillation from P_4O_{10}, while MeOH was distilled over Mg turnings and iodine. The dichloro(η^6-*p*-cymene)ruthenium(II) dimer (**Ru-dimer**, Strem Chemicals, Newburyport, MA, USA), the dichloro(pentamethylcyclopentadienyl)iridium(III) dimer (**Ir-dimer**, Acros Organics), the dichloro(pentamethylcyclopentadienyl)rhodium(III) dimer (**Rh-dimer**, Alfa Aesar) and $TlPF_6$ (Strem Chemicals) are commercially available chemicals purchased from the listed suppliers. The dichloro(η^6-*p*-cymene)osmium(II) dimer (**Os-dimer**) was prepared according to a literature method (Godó et al., 2012). The 1-(β-D-glucopyranosyl)-4-(pyridin-2-yl)- and -(quinolin-2-yl)-1,2,3-triazoles (**1** and **2**, respectively) were synthesized according to our earlier described procedures (Kacsir et al., 2021).

1.2 General procedure I for *O*-peracylation of the 1-(β-D-glucopyranosyl)-4-hetaryl-1,2,3-triazoles

A solution of the appropriate 1-(β-D-glucopyranosyl)-4-hetaryl-1,2,3-triazole (**1** or **2)** in anhydrous pyridine (4 mL / 50 mg substrate) was cooled down in an ice bath and the corresponding carboxylic acid chloride (4.8 equiv.) was added under stirring. The reaction mixture was then heated at 60 °C and monitored by TLC (1 : 1 $CHCl_3$-MeOH and 1 : 2 EtOAc-hexane). If the TLC showed incomplete conversion after one hour, an additional portion of acid chloride (4.8 equiv.) was added to the mixture. After completion of the reaction the pyridine was removed in vacuo. The residue was dissolved in $CHCl_3$ (30 mL) and extracted with sat. aq. solution of $NaHCO_3$ (2 × 30 mL) and with water (35 mL). The separated organic phase was dried over $MgSO_4$, filtered and evaporated. The residual crude product was purified by column chromatography.

1.3 General procedure II for the preparation of the [(η^6-*p*-cym)M^{II}(N-N)Cl]PF_6 (M = Ru, Os) and [(η^5-Cp*)M^{III}(N-N)Cl]PF_6 (M = Ir, Rh) type complexes

The corresponding *O*-peracylated 1-(β-D-gucopyranosyl)-4-hetaryl-1,2,3-triazole (**L-2**–**L-7**, 2.0 or 2.1 equiv.), the complex dimer (**Ru-/Os-/Ir-/Rh-dimer**, 1 equiv.) and $TlPF_6$ (2 equiv.) were dissolved in a 1 : 1 mixture of anhydrous CH_2Cl_2 and MeOH (1-1 mL / 10 mg dimer). The reaction mixture was vigorously stirred until the TLC (95 : 5 $CHCl_3$-MeOH) showed complete disappearance of the starting dimer complex. After completion of the reaction, the precipitated TlCl was filtered off and the solvents were removed. The residual crude product was purified by crystallization or by column chromatography.

1.4 Syntheses and characterization of the new compounds

1-(2’,3’,4’,6’-Tetra-*O*-butanoyl-β-D-glucopyranosyl)-4-(pyridin-2-yl)-1,2,3-triazole (L-2)

Prepared from triazole **1** (0.050 g, 0.16 mmol) and butyryl chloride (2 × 81 µL, 2 × 0.78 mmol) according to general procedure I. Reaction time: 3 h. Purified by column chromatography (1 : 3 EtOAc-hexane) to give 82 mg (87 %) white amorphous solid. R_f = 0.47 (1 : 2 EtOAc-hexane). ^{1}H NMR (360 MHz, $CDCl_3$) δ (ppm): 8.61 (1H, d, *J* = 4.7 Hz, Py-H-6), 8.43 (1H, s, Tria-H-5), 8.13 (1H, d, *J* = 7.9 Hz, Py-H-3), 7.77 (1H, t, *J* = 7.6 Hz, Py-H-4), 7.25 (1H, dd, *J* = 7.6, 4.7 Hz, Py-H-5), 5.97 (1H, d, *J* = 8.7 Hz, H-1’), 5.54, 5.50, 5.32 (3 × 1H, 3 pt, *J* = 9.6, 9.5 Hz in each, H-2’, H-3’, H-4’), 4.30 (1H, dd, *J* = 12.6, 4.8 Hz, H-6’a), 4.18 (1H, dd, *J* = 12.6, < 1 Hz, H-6’b), 4.05 (1H, ddd, *J* = 9.6, 4.8, < 1 Hz, H-5’), 2.36-2.23 (6H, m, 3 × CH_2), 2.11 (2H, t, *J* = 7.4 Hz, CH_2), 1.70-1.53 (6H, m, 3 × CH_2), 1.41 (2H, sext, *J* = 7.4 Hz, CH_2), 0.96-0.88 (9H, m, 3 × CH_3), 0.72 (3H, t, *J* = 7.4 Hz, CH_3); ^{13}C NMR (90 MHz, $CDCl_3$) δ (ppm): 173.0, 172.3, 171.8, 171.3 (4 × C=O), 149.5, 148.8 (Tria-C-4, Py-C-2), 149.5 (Py-C-6), 136.8, 123.0, 120.2 (Py-C-3, Py-C-4, Py-C-5), 120.5 (Tria-C-5), 85.9 (C-1’), 75.2, 72.2, 70.1, 67.3 (C-2’ – C-5’), 61.2 (C-6’), 35.7 (2), 35.6, 35.3 (4 × CH_2), 18.1,, 18.0 (2), 17.9 (4 × CH_2), 13.5 (2), 13.4, 13.2 (4 × CH_3). ESI-HRMS positive mode (m/z): cald for: $C_{29}H_{40}N_4O_9Na^+$ $[M+Na]^+$ 611.2686. Found: 611.2687.

1-(2’,3’,4’,6’-Tetra-*O*-pentanoyl-β-D-glucopyranosyl)-4-(pyridin-2-yl)-1,2,3-triazole (L-3)

Prepared from triazole **1** (0.050 g, 0.16 mmol) and valeryl chloride (2 × 95 µL, 2 × 0.78 mmol) according to general procedure I. Reaction time: 1.5 h. Purified by column chromatography (1 : 3 EtOAc-hexane) to give 57 mg (55 %) pale yellow amorphous solid. R_f = 0.58 (3 : 7 EtOAc-hexane). ^{1}H NMR (360 MHz, $CDCl_3$) δ (ppm): 8.61 (1H, d, *J* = 4.5 Hz, Py-H-6), 8.41 (1H, s, Tria-H-5), 8.14 (1H, d, *J* = 7.9 Hz, Py-H-3), 7.78 (1H, dt, *J* = 7.9, 1.5 Hz, Py-H-4), 7.25 (1H, ddd, *J* = 7.9, 4.5, 1.5 Hz, Py-H-5), 5.95 (1H, d, *J* = 8.7 Hz, H-1’), 5.53, 5.49, 5.31 (3 × 1H, 3 pt, *J* = 9.5, 9.4 Hz in each, H-2’, H-3’, H-4’), 4.28 (1H, dd, *J* = 12.6, 4.8 Hz, H-6’a), 4.18 (1H, dd, *J* = 12.6, 1.2 Hz, H-6’b), 4.03 (1H, ddd, *J* = 9.6, 4.8, 1.2 Hz, H-5’), 2.38-2.24 (6H, m, 3 × CH_2), 2.13 (2H, t, *J* = 7.4 Hz, CH_2), 1.64-1.24 (14H, m, 7 × CH_2), 1.36 (2H, sext, *J* = 7.3 Hz, CH_2), 0.94-0.87 (9H, m, 3 × CH_3), 0.75 (3H, t, *J* = 7.3 Hz, CH_3); ^{13}C NMR (90 MHz, $CDCl_3$) δ (ppm): 173.3, 172.6, 172.0, 171.6 (4 × C=O), 149.5, 148.9 (Tria-C-4, Py-C-2), 149.4 (Py-C-6), 136.8, 123.1, 120.2 (Py-C-3, Py-C-4, Py-C-5), 120.5 (Tria-C-5), 85.9 (C-1’), 75.2, 72.2, 70.2, 67.3 (C-2’ – C-5’), 61.3 (C-6’), 33.6, 33.5 (2), 33.3 (4 × CH_2), 26.7 (3), 26.5 (4 × CH_2), 22.1 (3), 21.8 (4 × CH_2), 13.6, 13.5 (2), 13.4 (4 × CH_3). ESI-HRMS positive mode (m/z): cald for: $C_{33}H_{48}N_4O_9Na^+$ $[M+Na]^+$ 667.3313. Found: 667.3311.

1-(2’,3’,4’,6’-Tetra-*O*-hexanoyl-β-D-glucopyranosyl)-4-(pyridin-2-yl)-1,2,3-triazole (L-4)

Prepared from triazole **1** (0.050 g, 0.16 mmol) and hexanoyl chloride (2 × 110 µL, 2 × 0.78 mmol) according to general procedure I. Reaction time: 3 h. Purified by column chromatography (1 : 4 EtOAc-hexane) to give 80 mg (71 %) pale yellow amorphous solid. R_f = 0.69 (3 : 7 EtOAc-hexane). ^{1}H NMR (360 MHz, $CDCl_3$) δ (ppm): 8.61 (1H, d, *J* = 4.9 Hz, Py-H-6), 8.40 (1H, s, Tria-H-5), 8.14 (1H, d, *J* = 7.9 Hz, Py-H-3), 7.78 (1H, dt, *J* = 7.7, 1.7 Hz, Py-H-4), 7.25 (1H, ddd, *J* = 7.7, 4.9, 1 < Hz, Py-H-5), 5.94 (1H, d, *J* = 8.9 Hz, H-1’), 5.52, 5.47, 5.29 (3 × 1H, 3 pt, *J* = 9.6, 9.5 Hz in each, H-2’, H-3’, H-4’), 4.27 (1H, dd, *J* = 12.6, 4.7 Hz, H-6’a), 4.18 (1H, dd, *J* = 12.6, 1.9 Hz, H-6’b), 4.03 (1H, ddd, *J* = 9.6, 4.7, < 1 Hz, H-5’), 2.36-2.23 (6H, m, 3 × CH_2), 2.12 (2H, t, *J* = 7.5 Hz, CH_2), 1.65-1.20, (20H, m, 10 × CH_2), 1.19-1.11 (2H, m, CH_2), 1.07-1.01 (2H, m, CH_2), 0.92-0.86 (9H, m, 3× CH_3), 0.75 (3H, t, *J* = 7.3 Hz, CH_3); ^{13}C NMR (90 MHz, $CDCl_3$) δ (ppm): 173.3, 172.6, 172.0, 171.6 (4 × C=O), 149.6, 148.9 (Tria-C-4, Py-C-2), 149.5 (Py-C-6), 136.8, 123.1, 120.3 (Py-C-3, Py-C-4, Py-C-5), 120.5 (Tria-C-5), 85.9 (C-1’), 75.3, 72.3, 70.2, 67.4 (C-2’ – C-5’), 61.3 (C-6’), 33.9, 33.8

(2), 33.5 (4 × CH_2), 31.2 (2), 31.1, 30.8 (4 × CH_2), 24.3 (3), 24.2 (4 × CH_2), 22.2 (2), 22.1, 22.0 (4 × CH_2), 13.9, 13.8, 13.7, 13.6 (4 × CH_3). ESI-HRMS positive mode (m/z): cald for: $C_{37}H_{56}N_4O_9Na^+$ $[M+Na]^+$ 723.3940. Found: 723.3937.

1-(2’,3’,4’,6’-Tetra-*O*-heptanoyl-β-D-glucopyranosyl)-4-(pyridin-2-yl)-1,2,3-triazole (L-5)

Prepared from triazole **1** (0.050 g, 0.16 mmol) and heptanoyl chloride (2 × 110 μL, 2 × 0.71 mmol) according to general procedure I. Reaction time: 2 h. Purified by column chromatography (2 : 7 EtOAc-hexane) to give 97 mg (80 %) yellow amorphous solid. R_f = 0.51 (3 : 7 EtOAc-hexane). ^1H NMR (360 MHz, $CDCl_3$) δ (ppm): 8.61 (1H, d, *J* = 4.2 Hz, Py-H-6), 8.42 (1H, s, Tria-H-5), 8.14 (1H, d, *J* = 7.8 Hz, Py-H-3), 7.77 (1H, t, *J* = 7.8 Hz, Py-H-4), 7.26-7.23 (1H, m, Py-H-5), 5.97 (1H, d, *J* = 8.6 Hz, H-1’), 5.54, 5.49, 5.31 (3 × 1H, 3 pt, *J* = 9.5, 9.4 Hz in each, H-2’, H-3’, H-4’), 4.29 (1H, dd, *J* = 12.6, 4.7 Hz, H-6’a), 4.17 (1H, dd, *J* = 12.6, < 1 Hz, H-6’b), 4.07-4.04 (1H, m, H-5’), 2.37-2.24 (6H, m, 3 × CH_2), 2.12 (2H, t, *J* = 7.4 Hz, CH_2), 1.61-0.87 (41H, m, 16 × CH_2, 3 × CH_3), 0.78 (3H, t, *J* = 7.3 Hz, CH_3); ^{13}C NMR (90 MHz, $CDCl_3$) δ (ppm): 173.2, 172.5, 171.9, 171.5 (4 × C=O), 149.5, 148.8 (Tria-C-4, Py-C-2), 149.4 (Py-C-6), 136.7, 123.0, 120.2 (Py-C-3, Py-C-4, Py-C-5), 120.4 (Tria-C-5), 85.8 (C-1’), 75.2, 72.2, 70.1, 67.3 (C-2’ – C-5’), 61.2 (C-6’), 33.9, 33.8 (2), 33.5 (4 × CH_2), 31.3 (3), 31.1 (4 × CH_2), 28.6 (3), 28.3 (4 × CH_2), 24.6, 24.5 (2), 24.4 (4 × CH_2), 22.3, 22.3 (2), 22.2 (4 × CH_2), 13.9 (3), 13.8 (4 × CH_3). ESI-HRMS positive mode (m/z): cald for: $C_{41}H_{64}N_4O_9Na^+$ $[M+Na]^+$ 779.4566. Found: 779.4560.

1-(2’,3’,4’,6’-Tetra-*O*-octanoyl-β-D-glucopyranosyl)-4-(pyridin-2-yl)-1,2,3-triazole (L-6)

Prepared from triazole **1** (0.051 g, 0.17 mmol) and octanoyl chloride (2 × 135 μL, 2 × 0.78 mmol) according to general procedure I. Reaction time: 2.5 h. Purified by column chromatography (1 : 9 EtOAc-hexane) to give 105 mg (81 %) pale yellow amorphous solid. R_f = 0.45 (3 : 7 EtOAc-hexane). ^1H NMR (360 MHz, $CDCl_3$) δ (ppm): 8.62 (1H, dd, *J* = 4.9, 1.6 Hz, Py-H-6), 8.46 (1H, s, Tria-H-5), 8.16 (1H, d, *J* = 7.9 Hz, Py-H-3), 7.78 (1H, dt, *J* = 7.9, 1.6 Hz, Py-H-4), 7.26 (1H, ddd, *J* = 7.5, 4.9, < 1 Hz, Py-H-5), 5.95 (1H, d, *J* = 8.9 Hz, H-1’), 5.54, 5.48, 5.30 (3 × 1H, 3 pt, *J* = 9.9, 9.5 Hz in each, H-2’, H-3’, H-4’), 4.28 (1H, dd, *J* = 12.6, 4.9 Hz, H-6’a), 4.18 (1H, dd, *J* = 12.6, 1.8 Hz, H-6’b), 4.04 (1H, ddd, *J* = 9.9, 4.9, < 1 Hz, H-5’), 2.40-2.23 (6H, m, 3 × CH_2), 2.12 (2H, t, *J* = 7.4 Hz, CH_2), 1.71-0.78 (60H, m, 24 × CH_2, 4 × CH_3); ^{13}C NMR (90 MHz, $CDCl_3$) δ (ppm): 173.3, 172.6, 171.9, 171.5 (4 × C=O), 149.4, 148.5 (Tria-C-4, Py-C-2), 149.2 (Py-C-6), 137.0, 123.1, 120.4 (Py-C-3, Py-C-4, Py-C-5), 120.7 (Tria-C-5), 85.9 (C-1’), 75.3, 72.3, 70.1, 67.3 (C-2’ – C-5’), 61.3 (C-6’), 34.0, 33.9, 33.8, 33.5 (4 × CH_2), 31.6, 31.5 (2), 31.4 (4 × CH_2), 29.0 (3), 28.8 (3), 28.7, 28.6 (8 × CH_2), 24.7, 24.6, 24.6, 24.5 (4 × CH_2), 22.5 (3), 22.4 (4 × CH_2), 14.0 (3), 13.9, (4 × CH_3). ESI-HRMS positive mode (m/z): cald for: $C_{45}H_{72}N_4O_9Na^+$ $[M+Na]^+$ 835.5192. Found: 835.5192.

1-(2’,3’,4’,6’-Tetra-*O*-benzoyl-β-D-glucopyranosyl)-4-(quinolin-2-yl)-1,2,3-triazole (L-7)

Prepared from triazole **2** (100 mg, 0.279 mmol) and benzoyl chloride (157 μL, 1.35 mmol) according to general procedure I. Reaction time: 2 h. Purified by column chromatography (2 : 3 EtOAc-hexane) to give 207 mg (96 %) white amorphous solid. R_f = 0.40 (2 : 3 EtOAc-hexane). ^1H NMR (400 MHz, $CDCl_3$) δ (ppm): 8.80 (H, s, Tria-H-5), 8.27-7.26 (26H, m, Ar, Qu), 6.37 (1H, d, *J* = 8.8 Hz, H-1’), 6.17 (1H, pt, *J* = 9.5, 9.2 Hz, H-2’ or H-3’ or H-4’), 6.11 (1H, pt, *J* = 9.4, 9.0 Hz, H-2’ or H-3’ or H-4’), 5.92 (1H, pt, *J* = 9.5, 9.4 Hz, H-2’ or H-3’ or H-4’), 4.72-4.50 (3H, m, H-5’, H-6’a, H-6’b); ^{13}C NMR (100 MHz, $CDCl_3$) δ (ppm): 166.2, 165.8, 165.3, 164.8 (4 × C=O), 149.9, 149.4, 148.2 (Tria-C-4, Qu-C-2, Qu-C-8a), 137.1, 133.8, 133.7, 133.6, 133.4, 130.3-128.5, 128.1, 128.0, 127.8, 126.7, 121.7, 118.9 (Tria-C-5, Qu-C-3 – Qu-C-8, Qu-C-4a, Ar), 86.5 (C-1’), 75.8, 73.2, 71.4, 69.0 (C-2′ –

C-5′), 62.9 (C-6’). ESI-HRMS positive mode (m/z): calcd for: $C_{45}H_{35}N_4O_9^+$ $[M+H]^+$ 775.2399; $C_{45}H_{34}N_4O_9Na^+$ $[M+Na]^+$ 797.2218. Found: $[M+H]^+$ 775.2395; $[M+Na]^+$ 797.2211.

Complex Os-2

Prepared from ligand **L-2** (30.4 mg, 0.052 mmol), **Os-dimer** (21.0 mg, 0.026 mmol) and $TlPF_6$ (17.7 mg, 0.051 mmol) according to general procedure II. Reaction time: 1 h. Purified by column chromatography (95 : 5 $CHCl_3$-MeOH) to yield 56 mg (96 %) yellow syrup. Diastereomeric ratio: 1 : 1. R_f = 0.34 (95 : 5 $CHCl_3$-MeOH). ^{1}H NMR (400 MHz, $CDCl_3$) δ (ppm): 9.25, 9.23 (2 × 1H, 2 d, *J* = 6.5 Hz in each, 2 × Py-H-6), 9.02, 8.89 (2 × 1H, 2 s, 2 × Tria-H-5), 8.04-8.01 (2H, m, 2 × Py-H-3), 7.99-7.94 (2H, m, 2 × Py-H-4), 7.55, 7.52 (2 × 1H, 2 dd, *J* = 7.4, 6.5 Hz, 2 × Py-H-5), 6.20-5.86 (12H, m, 2 × H-1’, 2 × (H-2’ or H-3’ or H-4’), 2 × 4 × *p*-cym-CH), 5.50, 5.48, 5.39, 5.37 (2 × 2H, 2 × 2 pt, *J* = 9.4, 9.2 Hz in each, H-2’ and/or H-3’ and/or H-4’), 4.38-4.14 (6H, m, 2 × H-5’, 2 × H-6’a, 2 × H-6’b), 2.63, 2.59 (2 × 1H, 2 hept, *J* = 6.9 Hz in each, 2 × *i*-Pr-CH), 2.37-0.76 (74H, m, 16 × CH_2, 14 × CH_3); ^{13}C NMR (90 MHz, $CDCl_3$) δ (ppm): 173.3, 173.2, 172.4, 172.3, 172.1, 172.0, 171.9, 171.6 (2 × 4 × C=O), 155.7, 155.6 (2 × Py-C-6), 148.4, 148.3 (2), 147.9 (2 × Tria-C-4, 2 × Py-C-2), 140.5, 140.4 (2 × Py-C-4), 129.8 (2) (2 × Tria-C-5), 127.7, 127.6 (2 × Py-C-5), 122.6 (2) (2 × Py-C-3), 96.9, 96.5, 95.7, 94.4 (2 × 2 × *p*-cym-C_{qAr}), 87.0, 86.6 (2 × C-1’), 78.4, 77.2, 77.0, 76.8, 75.7, 75.6, 75.5, 74.8, 74.4, 73.6, 72.9, 72.6, 70.1, 69.3, 67.3, 67.1 (2 × (C-2’ – C-5’), 2 × 4 × *p*-cym-CH_{Ar}), 61.3, 61.2 (2 × C-6’), 35.9 (4), 35.8 (2), 35.7, 35.4 (2 × 4 × CH_2), 31.2, 31.1 (2 × *i*-Pr-*C*H), 22.9, 22.5, 22.3, 21.9 (2 × 2 × *i*-Pr-*C*H_3), 18.6, 18.5 (2 × C_6H_4-*C*H_3), 18.4, 18.3 (4), 18.2, 18.1 (2) (2 × 4 × CH_2), 13.7 (3), 13.6 (3), 13.6, 13.5 (2 × 4 × CH_3). ESI-HRMS positive mode (m/z): cald for: $C_{39}H_{54}ClN_4O_9Os^+$ $[M-PF_6]^+$ 949.3180. Found: 949.3180.

Complex Os-3

Prepared from ligand **L-3** (33.1 mg, 0.052 mmol), **Os-dimer** (20.0 mg, 0.025 mmol) and $TlPF_6$ (17.6 mg, 0.050 mmol) according to general procedure II. Reaction time: 1 h. Purified by column chromatography (95 : 5 $CHCl_3$-MeOH) to yield 52 mg (88 %) yellow syrup. Diastereomeric ratio: 4 : 3. R_f = 0.38 (95 : 5 $CHCl_3$-MeOH). ^{1}H NMR (400 MHz, $CDCl_3$) δ (ppm): 9.25, 9.23 (2 × 1H, 2 d, *J* = 6.6 Hz in each, 2 × Py-H-6), 9.03, 8.90 (2 × 1H, 2 s, 2 × Tria-H-5), 8.04-8.01 (2H, m, 2 × Py-H-3), 7.99-7.94 (2H, m, 2 × Py-H-4), 7.55-7.50 (2H, m, 2 × Py-H-5), 6.20-5.86 (12H, m, 2 × H-1’, 2 × (H-2’ or H-3’ or H-4’), 2 × 4 × *p*-cym-CH), 5.51, 5.47, 5.40, 5.35 (2 × 2H, 2 × 2 pt, *J* = 9.4, 9.2 Hz in each, H-2’ and/or H-3’ and/or H-4’), 4.38-4.14 (6H, m, 2 × H-5’, 2 × H-6’a, 2 × H-6’b), 2.67-2.55 (2H, m, 2 × *i*-Pr-CH), 2.40-0.77 (90H, m, 24 × CH_2, 14 × CH_3); ^{13}C NMR (90 MHz, $CDCl_3$) δ (ppm): 173.4, 173.3, 172.5, 172.4, 172.2, 172.1, 172.0, 171.7 (2 × 4 × C=O), 155.7, 155.5 (2 × Py-C-6), 148.3, 148.2 (2), 147.8 (2 × Tria-C-4, 2 × Py-C-2), 140.4, 140.3 (2 × Py-C-4), 129.8, 129.6 (2 × Tria-C-5), 127.6, 127.5 (2 × Py-C-5), 122.6 (2) (2 × Py-C-3), 96.8, 96.4, 95.8, 94.3 (2 × 2 × *p*-cym-C_{qAr}), 86.9, 86.5 (2 × C-1’), 78.4, 76.9 (3), 75.7 (2), 75.6, 74.7, 74.3, 73.4, 72.8, 72.5, 70.0, 69.3, 67.2, 67.0 (2 × (C-2’ – C-5’), 2 × 4 × *p*-cym-CH_{Ar}), 61.3, 61.1 (2 × C-6’), 33.7 (3), 33.6 (2), 33.5, 33.3 (2) (2 × 4 × CH_2), 31.1, 31.0 (2 × *i*-Pr-*C*H), 26.8 (2), 26.8 (2), 26.7 (2), 26.6, 26.6 (2 × 4 × CH_2), 22.9, 22.4, 22.3, 21.8 (2 × 2 × *i*-Pr-*C*H_3), 22.2 (4), 22.1 (2), 22.0 (2) (2 × 4 × CH_2), 18.6 (2) (2 × C_6H_4-*C*H_3), 13.8 (3), 13.7 (3), 13.6 (2), (2 × 4 × CH_3). ESI-HRMS positive mode (m/z): cald for: $C_{43}H_{62}ClN_4O_9Os^+$ $[M-PF_6]^+$ 1005.3807. Found: 1005.3806.

Complex Os-4

Prepared from ligand **L-4** (18.6 mg, 0.027 mmol), **Os-dimer** (10.0 mg, 0.013 mmol) and $TlPF_6$ (8.8 mg, 0.025 mmol) according to general procedure II. Reaction time: 1 h. Purified by column chromatography (95 : 5 $CHCl_3$-MeOH) to yield 25 mg (80 %) yellow syrup. Diastereomeric ratio: 5 :

4. R_f = 0.40 (95 : 5 $CHCl_3$-MeOH). ^{1}H NMR (400 MHz, $CDCl_3$) δ (ppm): 9.22, 9.20 (2 × 1H, 2 d, *J* = 6.4 Hz in each, 2 × Py-H-6), 8.99, 8.86, (2 × 1H, 2 s, 2 × Tria-H-5), 8.03-8.00 (2H, m, 2 × Py-H-3), 7.97-7.93 (2H, m, 2 × Py-H-4), 7.53-7.48 (2 × 1H, m, 2 × Py-H-5), 6.18-5.86 (12H, m, 2 × H-1', 2 × H-2' or H-3' or H-4', 2 × 4 × *p*-cym-CH), 5.49, 5.47 (2 × 1H, 2 pt, *J* = 9.1, 9.1 Hz in each, H-2' or H-3' or H-4'), 5.37, 5.35 (2 × 1H, 2 pt, *J* = 10.1, 10.1 Hz in each, H-2' or H-3' or H-4'), 4.39-4.13 (6H, m, 2 × H-5', 2 × H-6'a, 2 × H-6'b), 2.69-2.56 (2H, m, 2 × *i*-Pr-CH), 2.38-0.78 (106H, m, 32 × CH_2, 14 × CH_3); ^{13}C NMR (90 MHz, $CDCl_3$) δ (ppm): 173.5, 173.4, 172.6, 172.4, 172.3, 172.2, 172.1, 171.8 (2 × 4 × C=O), 155.6, 155.4 (2 × Py-C-6), 148.4 (3), 147.8 (2 × Tria-C-4, 2 × Py-C-2), 140.5, 140.4 (2 × Py-C-4), 127.7, 127.5 (2 × Py-C-5), 126.1, 125.8 (2 × Tria-C-5), 122.8, 122.7 (2 × Py-C-3), 97.0, 96.5, 95.7, 94.3 (2 × 2 × *p*-cym-C_{qAr}), 87.1, 86.7 (2 × C-1'), 78.5, 77.2, 77.0, 76.8, 75.9, 75.8, 75.7, 74.9, 74.5, 73.6, 72.9, 72.6, 70.1, 69.4, 67.3, 67.2 (2 × (C-2' – C-5'), 2 × 4 × *p*-cym-CH_{Ar}), 61.4, 61.2 (2 × C-6'), 34.1 (4), 34.9 (2), 34.8, 33.6 (2 × 4 × CH_2), 31.4 (4), 31.3 (3), 31.2 (2 × 4 × CH_2), 31.2 (2) (2 × *i*-Pr-CH), 24.5 (3), 24.4 (2). 24.3 (3) (2 × 4 × CH_2), 22.9, 22.5, 22.4, 21.9 (2 × 2 × *i*-Pr-*C*H_3), 22.4 (4), 22.3 (2), 22.2 (2) (2 × 4 × CH_2), 18.7, 18.6 (2 × C_6H_4-*C*H_3), 14.0 (2), 13.9 (4), 13.8 (2) (2 × 4 × CH_3). ESI-HRMS positive mode (m/z): cald for: $C_{47}H_{70}ClN_4O_9Os^+$ $[M-PF_6]^+$ 1061.4433. Found: 1061.4432.

Complex Os-5

Prepared from ligand **L-5** (38.4 mg, 0.051 mmol), **Os-dimer** (20.2 mg, 0.025 mmol) and $TlPF_6$ (17 mg, 0.049 mmol) according to general procedure II. Reaction time: 1 h. Purified by column chromatography (95 : 5 $CHCl_3$-MeOH) to yield 34 mg (53 %) yellow syrup. Diastereomeric ratio: 9 : 7. R_f = 0.48 (95 : 5 $CHCl_3$-MeOH). ^{1}H NMR (400 MHz, $CDCl_3$) δ (ppm): 9.23, 9.21 (2 × 1H, 2 d, *J* = 6.6 Hz in each, 2 × Py-H-6), 9.02, 8.88 (2 × 1H, 2 s, 2 × Tria-H-5), 8.03, 8.01 (2 × 1H, 2 d, *J* = 7.3 Hz, 2 × Py-H-3), 7.95, 7.95 (2 × 1H, 2 t, *J* = 7.7 Hz, 2 × Py-H-4), 7.53-7.49 (2H, m, 2 × Py-H-5), 6.18-5.85 (12H, m, 2 × H-1', 2 × (H-2' or H-3' or H-4'), 2 × 4 × *p*-cym-CH), 5.50, 5.45 (2 × 1H, 2 pt, *J* = 9.1, 9.1 Hz in each, H-2' or H-3' or H-4'), 5.39, 5.34 (2 × 1H, 2 pt, *J* = 10.1, 10.1 Hz in each, H-2' or H-3' or H-4'), 4.39-4.13 (6H, m, 2 × H-5', 2 × H-6'a, 2 × H-6'b), 2.69-2.56 (2 × 1H, m, 2 × *i*-Pr-CH), 2.38-0.78 (122H, m, 40 × CH_2, 14 × CH_3); ^{13}C NMR (90 MHz, $CDCl_3$) δ (ppm): 173.4, 173.3, 172.5, 172.4, 172.2, 172.1, 172.1, 171.7 (2 × 4 × C=O), 155.5, 155.3 (2 × Py-C-6), 148.3, 148.2, 147.7 (2) (2 × Tria-C-4, 2 × Py-C-2), 140.4, 140.3 (2 × Py-C-4), 129.8, 129.7 (2 × Tria-C-5), 127.5, 127.4 (2 × Py-C-5), 122.7, 122.6 (2 × Py-C-3), 96.9, 96.4, 95.6, 94.2 (2 × 2 × *p*-cym-C_{qAr}), 87.0, 86.6 (2 × C-1'), 78.4, 76.9, 75.8, 75.7, 75.7, 75.6, 74.8, 74.4, 73.5, 72.8, 72.6 (2), 70.0, 69.3, 67.2, 67.0 (2 × (C-2' – C-5'), 2 × 4 × *p*-cym-CH_{Ar}), 61.3, 61.1 (2 × C-6'), 34.0 (3), 33.9 (2), 33.8, 33.6 (2) (2 × 4 × CH_2), 31.5 (2), 31.4 (4), 31.4, 31.3 (2 × 4 × CH_2), 31.1, 31.0 (2 × *i*-Pr-*C*H), 28.8 (2), 28.8 (4), 28.7, 28.6 (2 × 4 × CH_2), 24.8 (2), 24.7, 24.7 (2), 24.6, 24.6, 24.5 (2 × 4 × CH_2), 22.9, 22.3 (2), 21.9 (2 × 2 × *i*-Pr-*C*H_3), 22.5 (4), 22.4 (4), (2 × 4 × CH_2), 18.6 (2) (2 × C_6H_4-*C*H_3), 14.1 (3), 14.0 (4), 14.0 (2 × 4 × CH_3). ESI-HRMS positive mode (m/z): cald for: $C_{51}H_{78}ClN_4O_9Os^+$ $[M-PF_6]^+$ 1117.5060. Found: 1117.5059.

Complex Os-6

Prepared from ligand **L-6** (42.2 mg, 0.052 mmol), **Os-dimer** (20.0 mg, 0.025 mmol) and $TlPF_6$ (17.0 mg, 0.049 mmol) according to general procedure II. Reaction time: 1 h. Purified by column chromatography (95 : 5 $CHCl_3$-MeOH) to yield 48 mg (72 %) yellow syrup. Diastereomeric ratio: 5 : 4. R_f = 0.38 (95 : 5 $CHCl_3$-MeOH). ^{1}H NMR (400 MHz, $CDCl_3$) δ (ppm): 9.24, 9.23 (2 × 1H, 2 d, *J* = 6.2 Hz in each, 2 × Py-H-6), 9.03, 8.90 (2 × 1H, 2 s, 2 × Tria-H-5), 8.04-8.01 (2H, m, 2 × Py-H-3), 7.95, 7.95 (2 × 1H, 2 pt, *J* = 7.4 Hz in each, 2 × Py-H-4), 7.54-7.49 (2H, m, 2 × Py-H-5), 6.19-5.86

(12H, m, 2 × H-1’, 2 × (H-2’ or H-3’ or H-4’), 2 × 4 × *p*-cym-CH), 5.50, 5.46 (2 × 1H, 2 pt, *J* = 9.4, 9.5 Hz in each, H-2’ or H-3’ or H-4’), 5.39, 5.34 (2 × 1H, 2 pt, *J* = 9.9, 10.0 Hz in each, H-2’ or H-3’ or H-4’), 4.40-4.15 (6H, m, 2 × H-5’, 2 × H-6’a, 2 × H-6’b), 2.62, 2.60 (2 × 1H, 2 hept, *J* = 6.9 Hz in each, 2 × *i*-Pr-CH), 2.38-0.81 (138H, m, 48 × CH_2, 14 × CH^3); ^{13}C NMR (90 MHz, $CDCl_3$) δ (ppm): 173.4, 173.3, 172.5, 172.4, 172.2, 172.1, 172.0, 171.8 (2 × 4 × C=O), 155.7, 155.6 (2 × Py-C-6), 148.3, 148.2 (2), 147.8 (2 × Tria-C-4, 2 × Py-C-2), 140.4, 140.3 (2 × Py-C-4), 129.8, 129.7 (2 × Tria-C-5), 127.6, 127.5 (2 × Py-C-5), 122.7, 122.6 (2 × Py-C-3), 96.9, 96.4, 95.8, 94.3 (2 × 2 × *p*-cym-C_{qAr}), 87.0, 86.6 (2 × C-1’), 78.4, 77.0, 76.9, 76.7, 75.7 (2), 75.6, 74.8, 74.4, 73.4, 72.8, 72.6, 70.0, 69.3, 67.2, 67.0 (2 × (C-2’ – C-5’), 2 × 4 × *p*-cym-CH_{Ar}), 61.3, 61.1 (2 × C-6’), 34.0 (2), 34.0 (2), 33.9 (2), 33.8, 33.6 (2 × 4 × CH_2), 31.7 (2), 31.7 (4), 31.6, 31.6 (2 × 4 × CH_2), 31.1, 31.0 (2 × *i*-Pr-*C*H), 29.1, 29.1 (2), 29.0, 29.0, 28.9, 28.9, 28.8 (2 × 4 × CH_2), 24.8 (2), 24.8(2), 24.7, 24.7, 24.6, 24.6 (2 × 4 × CH_2), 22.9, 22.4 (2), 22.3, 21.9 (2 × 2 × *i*-Pr-$\mathit{C}H_3$), 22.6 (5), 22.6, 22.5 (2 × 4 × CH_2), 18.6 (2) (2 × C_6H_4-$\mathit{C}H_3$), 14.1 (8) (2 × 4 × CH_3). ESI-HRMS positive mode (m/z): cald for: $C_{55}H_{86}ClN_4O_9Os^+$ $[M-PF_6]^+$ 1173.5686. Found: 1173.5687.

Complex Ru-7

Prepared from ligand **L-7** (26.6 mg, 0.0343 mmol, 2.1 eq.), **Ru-dimer** (10 mg, 0.0163 mmol) and $TlPF_6$ (11.4 mg, 0.0326 mmol) according to general procedure II. After filtration and removal of the solvent, the residue was dissolved in $CHCl_3$ (3 mL) and Et_2O (6 mL) was added. The precipitated product was filtered off, then washed with a solvent mixture of $CHCl_3$-Et_2O = 1 : 1 (1 mL) to give 26.2 mg (67 %) brown powder. Diastereomeric ratio: 2 : 1. R_f = 0.42 (95 : 5 $CHCl_3$-MeOH). ^1H NMR (400 MHz, $CDCl_3$) δ (ppm): 9.32 (s, minor Tria-H-5), 9.28 (s, major Tria-H-5), 8.63 (d, *J* = 8.8 Hz, major Qu-H-8), 8.60 (d, *J* = 8.9 Hz, minor Qu-H-8), 8.27 (d, *J* = 8.5 Hz, major Qu-H-4), 8.26 (d, *J* = 8.4 Hz, minor Qu-H-4), 8.07-7.28 (m, minor and major Qu-H-3, Qu-H-5 – Qu-H-7, Ar), 6.66 (pt, *J* = 9.3, 9.3 Hz, major H-2’ or H-3’ or H-4’), 6.57 (d, *J* = 9.3 Hz, major H-1’), 6.53 (d, *J* = 8.8 Hz, minor H-1’), 6.28 (pt, *J* = 9.5, 9.5 Hz, minor H-2’ or H-3’ or H-4’), 6.17 (pt, *J* = 9.5, 9.4 Hz, major H-2’ or H-3’ or H-4’), 6.16 (pt, *J* = 9.5, 9.2 Hz, minor H-2’ or H-3’ or H-4’), 6.01 (pt, *J* = 10.0, 9.6 Hz, minor H-2’ or H-3’ or H-4’), 5.95 (pt, *J* = 10.0, 9.6 Hz, major H-2’ or H-3’ or H-4’), 5.72-5.45 (m, minor and major 4 × *p*-cym-CH_{Ar}), 4.83-4.55 (m, minor and major H-5’, H-6’a, H-6’b), 2.35 (hept, *J* = 6.9 Hz, minor *i*-Pr-CH), 2.12 (hept, *J* = 6.9 Hz, major *i*-Pr-CH), 2.10 (s, major C_6H_4-$C\mathit{H_3}$), 2.02 (s, minor C_6H_4-$C\mathit{H_3}$), 0.85, 0.81 (2 d, *J* = 6.9 Hz in each, 2 × minor *i*-Pr-$C\mathit{H_3}$), 0.69, 0.60 (2 d, *J* = 6.9 Hz in each, 2 × major *i*-Pr-$C\mathit{H_3}$); ^{13}C NMR (100 MHz, $CDCl_3$) δ (ppm): 166.3, 165.6, 165.2, 165.0 (minor 4 × C=O), 166.2, 165.5, 165.3, 165.2 (major 4 × C=O), 149.2, 148.4, 147.5 (major Tria-C-4, Qu-C-2, Qu-C-8a), 149.1, 148.3, 147.8 (minor Tria-C-4, Qu-C-2, Qu-C-8a), 141.1, 134.3, 133.8 133.5, 133.3, 133.0, 130.6-128.0, 118.8, 118.7 (minor and major Tria-C-5, Qu-C-5 – Qu-C-8, Qu-C-4a, Ar), 105.6, 103.0 (minor *p*-cym-C_{qAr}), 105.6, 103.6 (major *p*-cym-C_{qAr}), 87.3, 87.2, 87.0, 86.7, 85.1, 85.0, 84.5, 83.4, 82.8 (minor and major *p*-cym-CH_{Ar}, C-1’), 76.2, 72.7, 72.2, 68.8 (minor C-2’ – C-5’), 75.8, 73.6, 70.5, 68.6 (major C-2’ – C-5’), 62.9 (minor C-6’), 62.7 (major C-6’), 31.1 (minor *i*-Pr-*C*H), 31.0 (major *i*-Pr-*C*H), 22.5, 21.4 (minor *i*-Pr-$\mathit{C}H_3$), 22.3, 21.4 (major *i*-Pr-$\mathit{C}H_3$), 18.8 (major C_6H_4-$\mathit{C}H_3$), 18.7 (minor C_6H_4-$\mathit{C}H_3$). ESI-HRMS positive mode (m/z): calcd for: $C_{55}H_{48}ClN_4O_9Ru^+$ $[M-PF_6]^+$ 1045.2165. Found: 1045.2163.

Complex Os-7

Prepared from ligand **L-7** (20.6 mg, 0.0266 mmol, 2.1 eq.), **Os-dimer** (10.0 mg, 0.0126mmol) and $TlPF_6$ (8.8 mg, 0.0252 mmol) according to general method II. After filtration and removal of the solvent, the residue was dissolved in $CHCl_3$ (3 mL) and Et_2O (6 mL) was added. The precipitated product was filtered off, then washed with a solvent mixture of $CHCl_3$-Et_2O = 1 : 1 (2 mL) to give

21.7 mg (67 %) brown powder. Diastereomeric ratio: 10 : 9. R_f = 0.38 (95 : 5 $CHCl_3$-MeOH). ^{1}H NMR (400 MHz, $CDCl_3$) δ (ppm): 9.36, 9.26 (2 × 1H, 2 s, 2 × Tria-H-5), 8.52, 8.50 (2 × 1H, 2 d, *J* = 8.9 Hz in both, 2 × Qu-H-8), 8.24, 8.22 (2 × 1H, 2 d, *J* = 8.9 Hz in both, 2 × Qu-H-4), 8.08-7.28 (48H, m, 2 × 20 × Ar, 2 × Py-H-3 – Py-H-5), 6.55, 6.51 (2 × 1H, 2 d, *J* = 9.3 Hz in each, 2 × H-1'), 6.65, 6.26, 6.18, 6.17, 6.03-5.93 (2 × 3H, 4 pt, m, *J* = 9.4 Hz in each, 2 × H-2', H-3', H-4'), 6.03-5.93, 5.82, 5.79, 5.74, 5.71, 5.69, 5.66 (2 × 4H, m, 6 d, *J* = 5.7 Hz in each, 2 × 4 × *p*-cym-CH_{Ar}), 4.84-4.55 (2 × 3H, m, 2 × H-5', 2 × H-6'a, 2 × H-6'b), 2.22, 2.13 (2 × 3H, 2 s, 2 × C_6H_4-CH_3), 2.20, 1.91 (2 × 1H, 2 hept, *J* = 6.9 Hz in each, 2 × *i*-Pr-CH), 0.80, 0.73, 0.54, 0.51 (2 × 2 × 3H, 2 × 2 d, *J* = 6.9 Hz in each 2 × 2 × *i*-Pr-CH_3); ^{13}C NMR (100 MHz, $CDCl_3$) δ (ppm): 166.4, 166.2, 165.6, 165.4, 165.3, 165.2, 165.1, 164.9 (2 × 4 × C=O), 149.8, 148.9, 148.6, 148.1 (2 × Tria-C-4, Qu-C-2, Qu-C-8a), 141.5, 141.3 (2 × Qu-C-2), 134.3, 134.2, 133.8, 133.6, 133.5, 133.4, 133.3, 133.2, 130.4-127.9 (2 × Qu-C-4a, Qu-C-5 – Qu-C-8, Tria-C-5, Ar), 118.5, 118.4, (2 × Qu-C-3), 97.2, 96.7, 96.6, 96.1 (2 × 2 × *p*-cym-C_{qAr}), 87.3, 86.8 (2 × C-1'), 78.9, 78.7, 76.7, 76.2, 75.8, 75.4, 75.2, 74.1, 73.5, 73.2, 72.7, 72.0, 70.4, 68.8, 68.6 (2 × 4 × *p*-cym-CH_{Ar}, 2 × C-2 – C-5'), 62.8, 62.7 (2 × C-6'), 31.2, 31.1 (2 × *i*-Pr-*C*H), 22.9, 22.6, 21.7, 21.6 (2 × 2 × *i*-Pr-CH_3), 18.7, 18.6 (2 × C_6H_4-CH_3). ESI-HRMS positive mode (m/z): calcd for: $C_{55}H_{48}ClN_4O_9Os^+$ $[M-PF_6]^+$ 1135.2725. Found: 1135.2724.

Complex Ir-7

Prepared from ligand **L-7** (20.4 mg, 0.0264 mmol, 2.1 eq.), **Ir-dimer** (10.0 mg, 0.0126 mmol) and $TlPF_6$ (8.7 mg, 0.0249 mmol) according to general method II. After filtration and removal of the solvent, the residue was dissolved in $CHCl_3$ (3 mL) and Et_2O (6 mL) was added. The precipitated product was filtered off, then washed with a solvent mixture of $CHCl_3$-Et_2O = 1 : 1 (1 mL) to give 19.7 mg (61 %) yellow powder. Diastereomeric ratio: 5 : 3. R_f = 0.41 (95 : 5 $CHCl_3$-MeOH). ^{1}H NMR (400 MHz, $CDCl_3$) δ (ppm): 9.41 (s, minor Tria-H-5), 9.34 (s, major Tria-H-5), 8.38-7.24 (m, minor and major Qu-H-3 – Qu-H-8, Ar), 6.61 (d, *J* = 9.3 Hz, major H-1'), 6.58 (d, *J* = 9.9 Hz, minor H-1'), 6.46 (pt, *J* = 9.4, 9.4 Hz, major H-2' or H-3' or H-4'), 6.29-6.23 (m, minor H-2' and/or H-3' and/or H-4'), 6.15 (pt, *J* = 9.4, 9.4 Hz, major H-2' or H-3' or H-4'), 6.02 (pt, *J* = 9.8, 9.7 Hz, minor H-2' or H-3' or H-4'), 5.93 (pt, *J* = 9.8, 9.7 Hz, minor H-2' or H-3' or H-4'), 4.81–4.54 (m, minor and major H-5′, H-6′a, H-6′b), 1.52 (s, major Cp*-CH_3), 1.48 (s, minor Cp*-CH_3); ^{13}C NMR (100 MHz, $CDCl_3$) δ (ppm): 166.3, 165.5, 165.3, 165.0 (minor and major 4 × C=O), 149.7, 148.9, 145.7, (minor Tria-C-4, Qu-C-2, Qu-C-8a), 149.3, 149.1, 145.6 (major Tria-C-4, Qu-C-2, Qu-C-8a), 141.8, 141.6, 134.2, 134.1, 134.0, 133.8, 133.5, 133.4, 133.3, 132.7, 132.6, 130.3-127.9, 127.3, 118.8 (minor and major Tria-C-5, Qu-C-3 – Qu-C-8, Qu-C-4a, Ar), 90.0 (major Cp*), 89.9 (minor Cp*), 87.4 (minor C-1′), 87.2 (major C-1′), 76.1, 75.9, 73.7, 72.9, 71.7, 70.5, 68.7, 68.5 (minor and major C-2′–C-5′), 62.8 (major C-6′), 62.7 (minor C-6′), 9.1 (minor and major Cp*-CH_3). ESI-HRMS positive mode (m/z): calcd for: $C_{55}H_{49}ClN_4O_9Ir^+$ $[M-PF_6]^+$ 1137.2813. Found: $[M-PF_6]^+$ 1137.2810.

Complex Rh-7

Prepared from ligand **L-7** (26.3 mg, 0.0340 mmol, 2.1 eq.), **Rh-dimer** (10.0 mg, 0.0162 mmol) and $TlPF_6$ (11.3 mg, 0.0323 mmol) according to general method II. After filtration and removal of the solvent, the residue was dissolved in $CHCl_3$ (3 mL) and Et_2O (6 mL) was added. The precipitated product was filtered off, then washed with a solvent mixture of $CHCl_3$-Et_2O = 1 : 1 (2 mL) to give 26.2 mg (80 %) orange powder. Diastereomeric ratio: 5 : 3. R_f = 0.38 (95 : 5 $CHCl_3$-MeOH). ^{1}H NMR (400 MHz, $CDCl_3$) δ (ppm): 9.33 (s, minor Tria-H-5), 9.28 (s, major Tria-H-5), 8.40-7.22 (m, minor and major Qu-H-3 – Qu-H-8, Ar), 6.61 (d, *J* = 9.3 Hz, major H-1'), 6.57 (d, *J* = 8.6 Hz, minor H-1'), 6.52 (pt, *J* = 9.3, 9.2 Hz, major H-2' or H-3' or H-4'), 6.28 (pt, *J* = 9.6, 9.4 Hz, minor H-2' or

H-3’ or H-4’), 6.23 (pt, *J* = 9.5, 9.0 Hz, minor H-2’ or H-3’ or H-4’), 6.17 (pt, *J* = 9.5, 9.4 Hz, major H-2’ or H-3’ or H-4’), 6.02 (pt, *J* = 9.5, 9.5 Hz, minor H-2’ or H-3’ or H-4’), 5.94 (pt, *J* = 9.8, 9.7 Hz, major H-2’ or H-3’ or H-4’), 4.80–4.54 (m, minor and major H-5′, H-6′a, H-6′b), 1.51 (s, major Cp*-CH_3), 1.44 (s, minor Cp*-CH_3); ^{13}C NMR (100 MHz, $CDCl_3$) δ (ppm): 166.2 (2), 165.7, 165.5 156.3, 165.2, 165.0, 164.7 (minor and major 4 × C=O), 148.1, 148.0, 147.9, 147.5, 145.9 (2) (minor and major Tria-C-4, Qu-C-2, Qu-C-8a), 141.3, 141.2, 134.1, 133.8, 133.7, 133.6, 133.5, 133.4, 133.3, 133.2, 130.3-127.8, 127.3, 119.0 (minor and major Tria-C-5, Qu-C-3 – Qu-C-8, Qu-C-4a, Ar), 97.9, 97.8 (minor and major Cp*), 87.5 (minor C-1′), 87.0 (major C-1′), 76.2, 75.8, 73.7, 72.8, 71.8, 70.6, 68.7, 68.6 (minor and major C-2′–C-5′), 62.8 (minor C-6′), 62.7 (major C-6′), 9.3 (major Cp*-*C*H_3), 9.2 (minor Cp*-*C*H_3). ESI-HRMS positive mode (m/z): calcd for: $C_{55}H_{49}ClN_4O_9Rh^+$ $[M-PF_6]^+$ 1047.2243. Found: $[M-PF_6]^+$ 1047.2240.

2. Copies of ^{1}H and ^{13}C NMR spectra

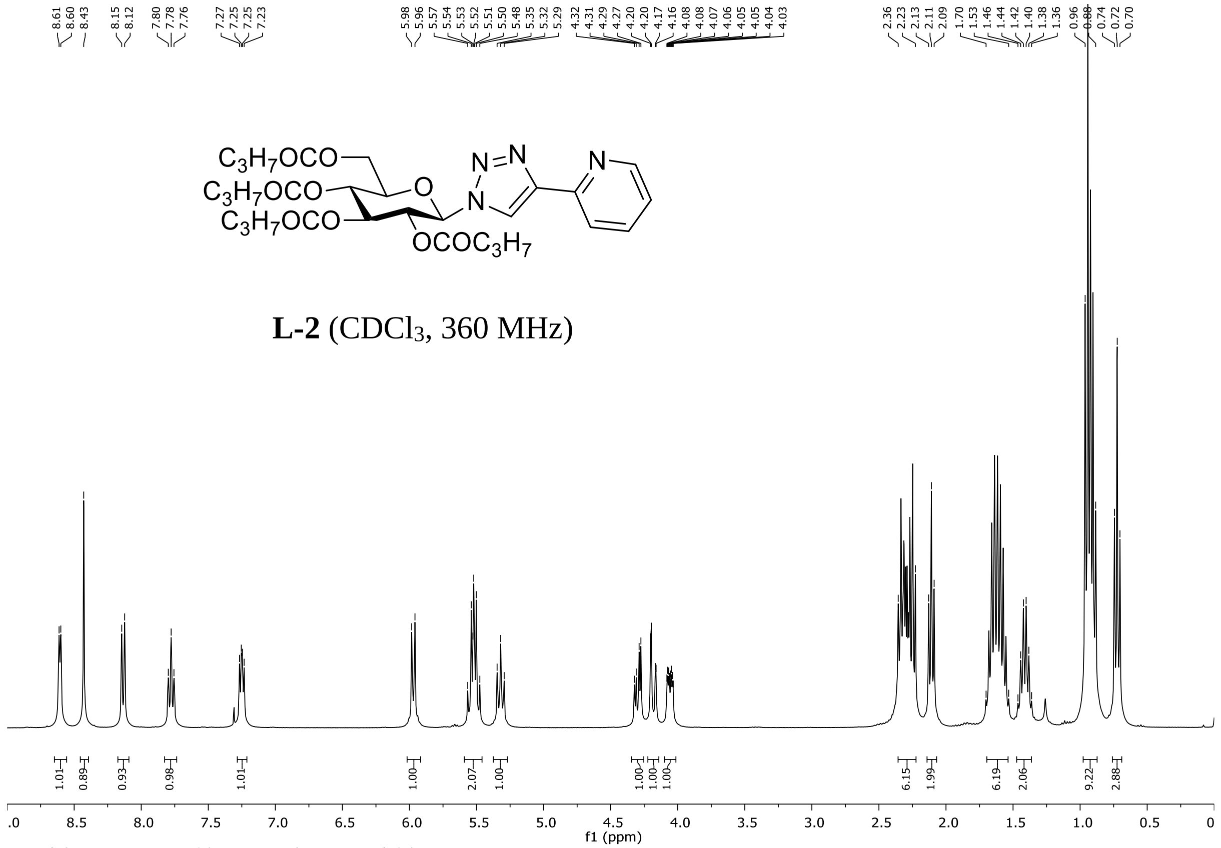

L-2 ($CDCl_3$, 360 MHz)

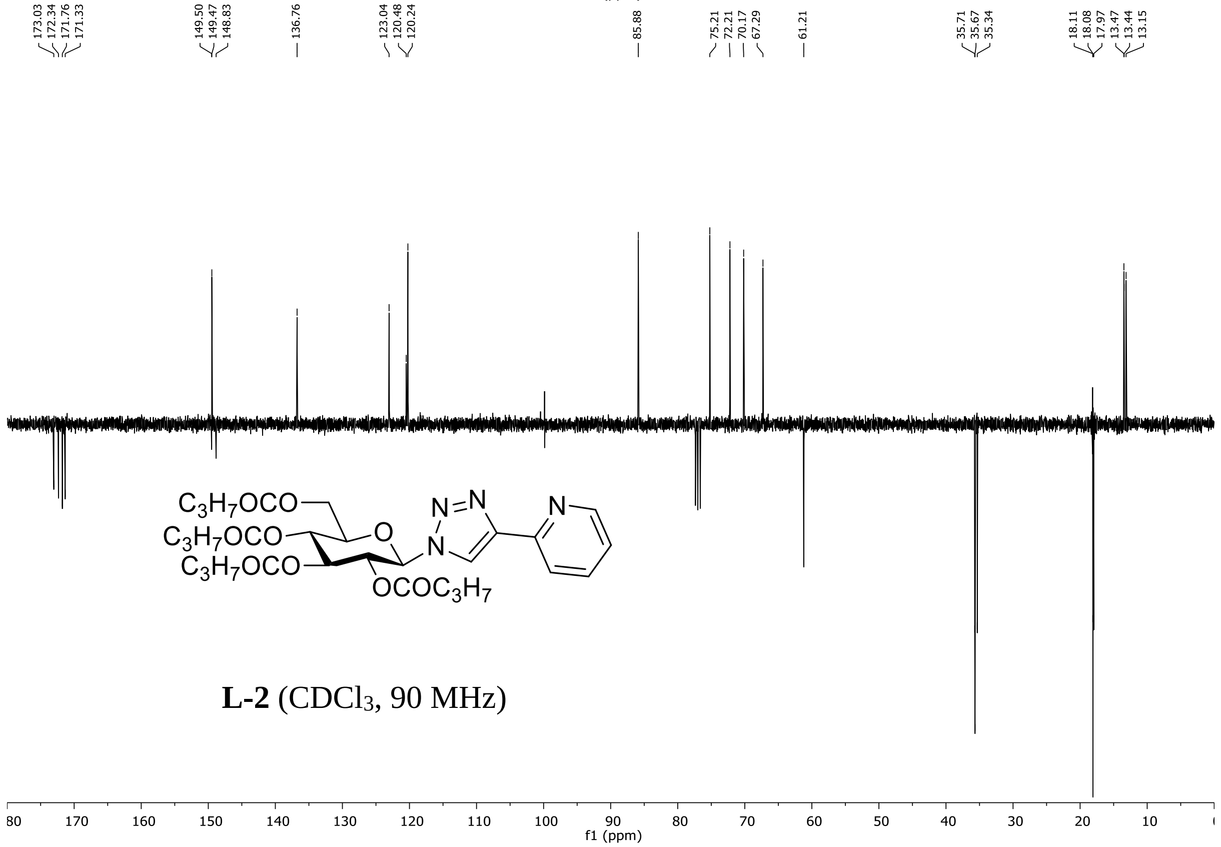

L-2 ($CDCl_3$, 90 MHz)

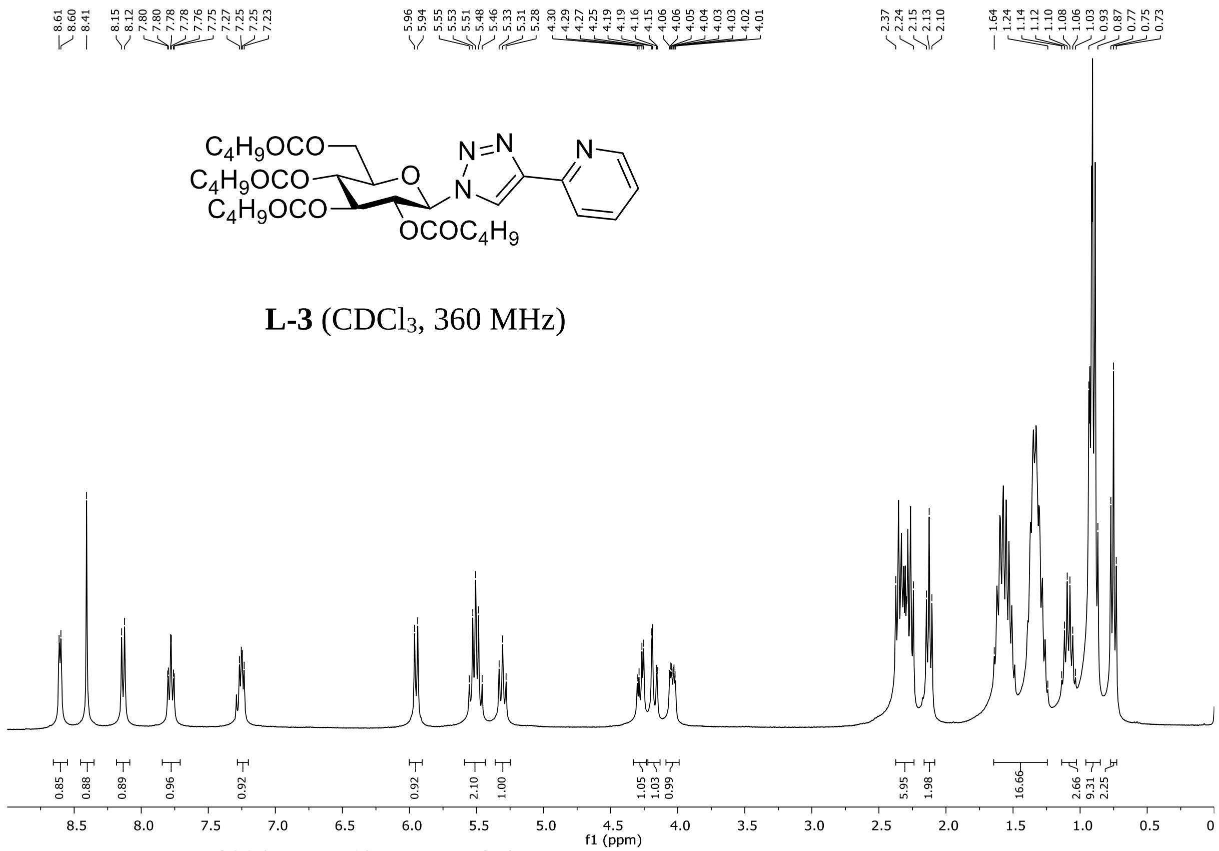

L-3 (CDCl$_3$, 360 MHz)

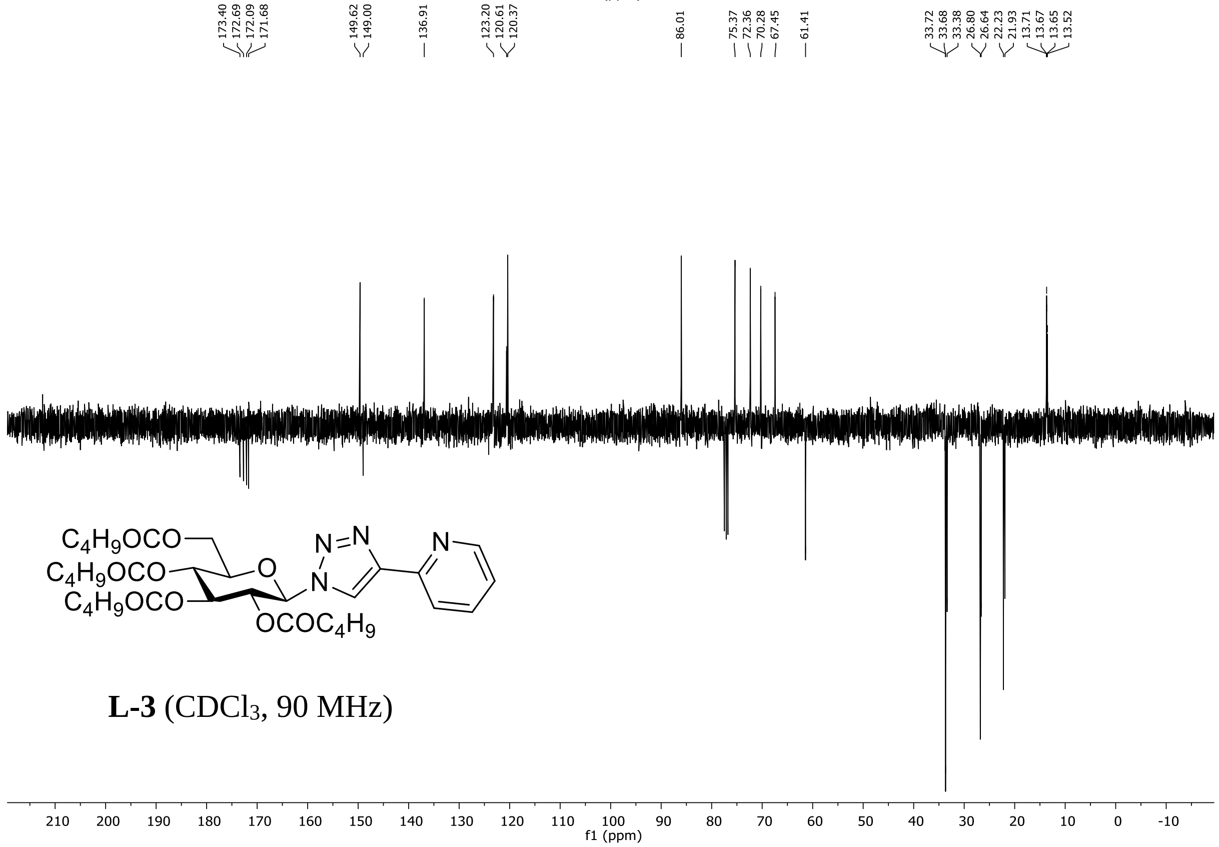

L-3 (CDCl$_3$, 90 MHz)

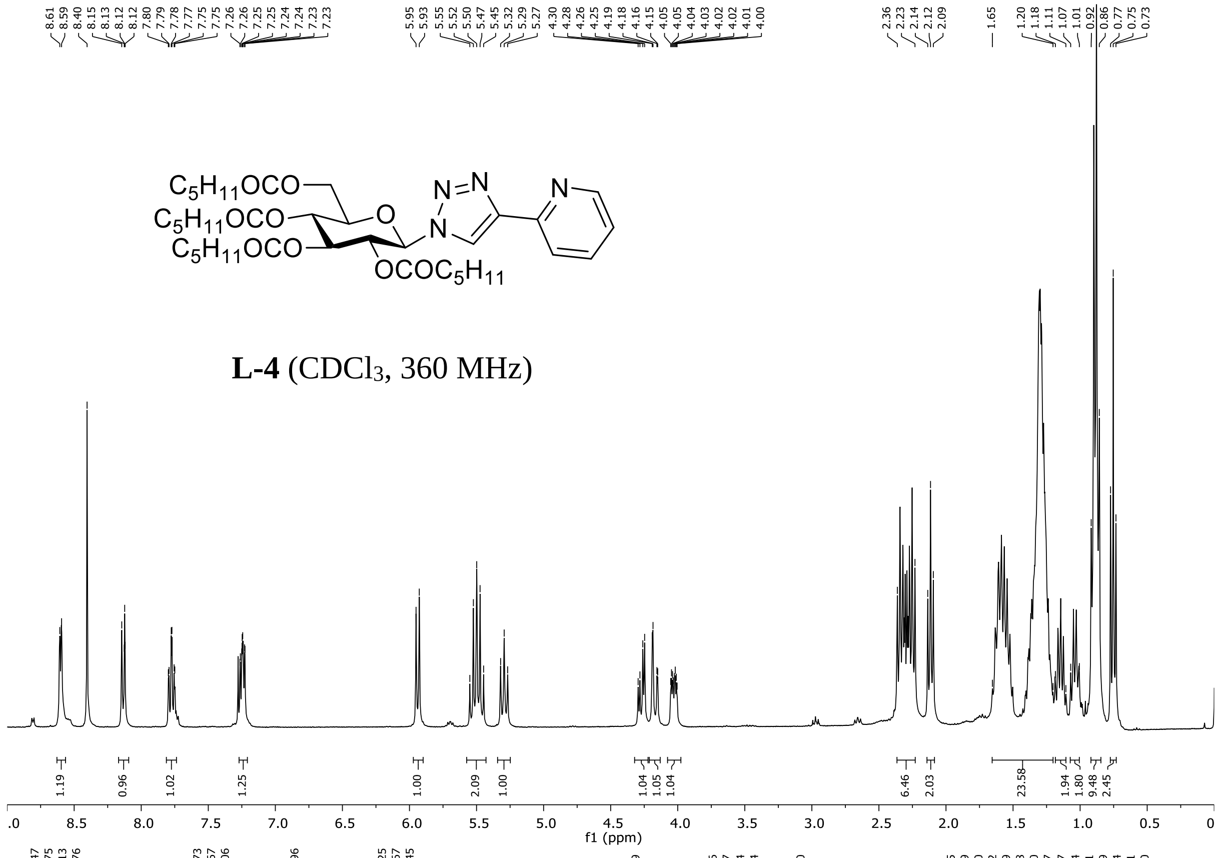
L-4 (CDCl3, 360 MHz)

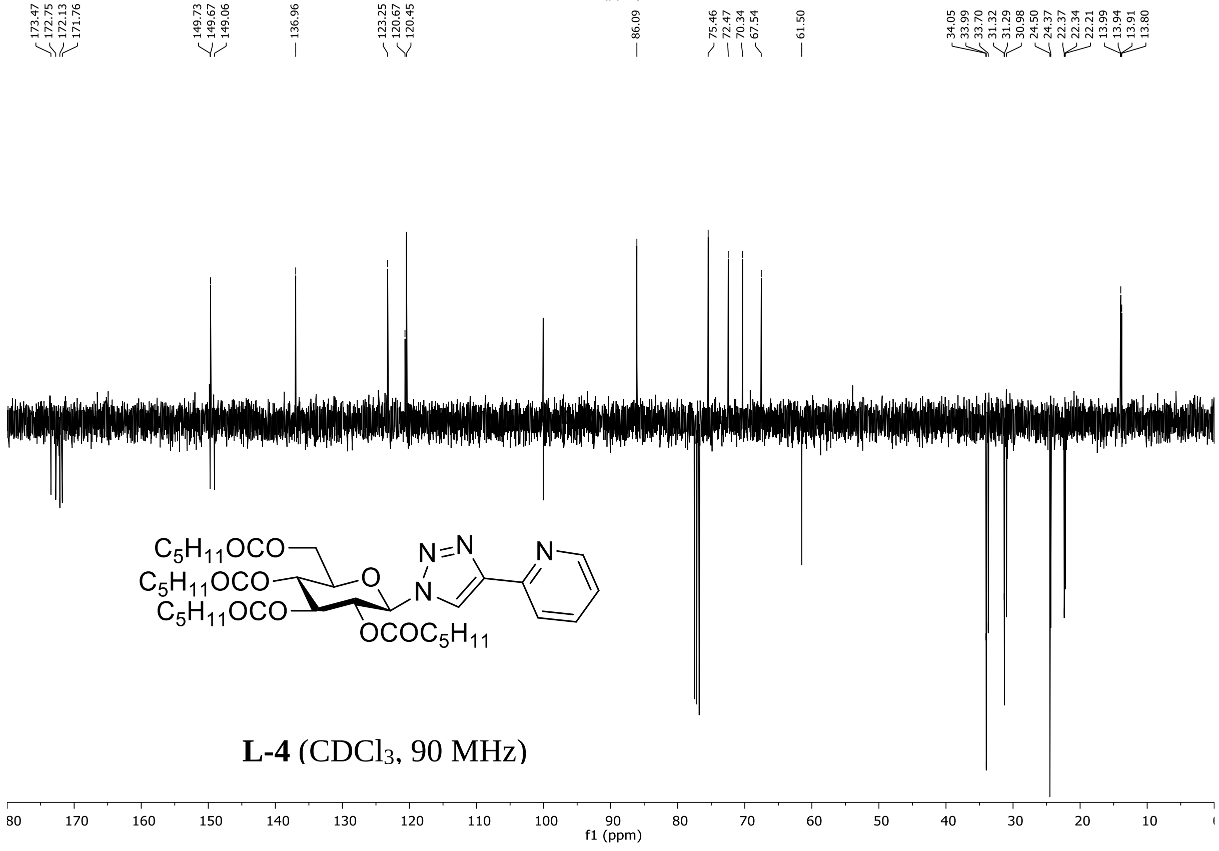
L-4 (CDCl3, 90 MHz)

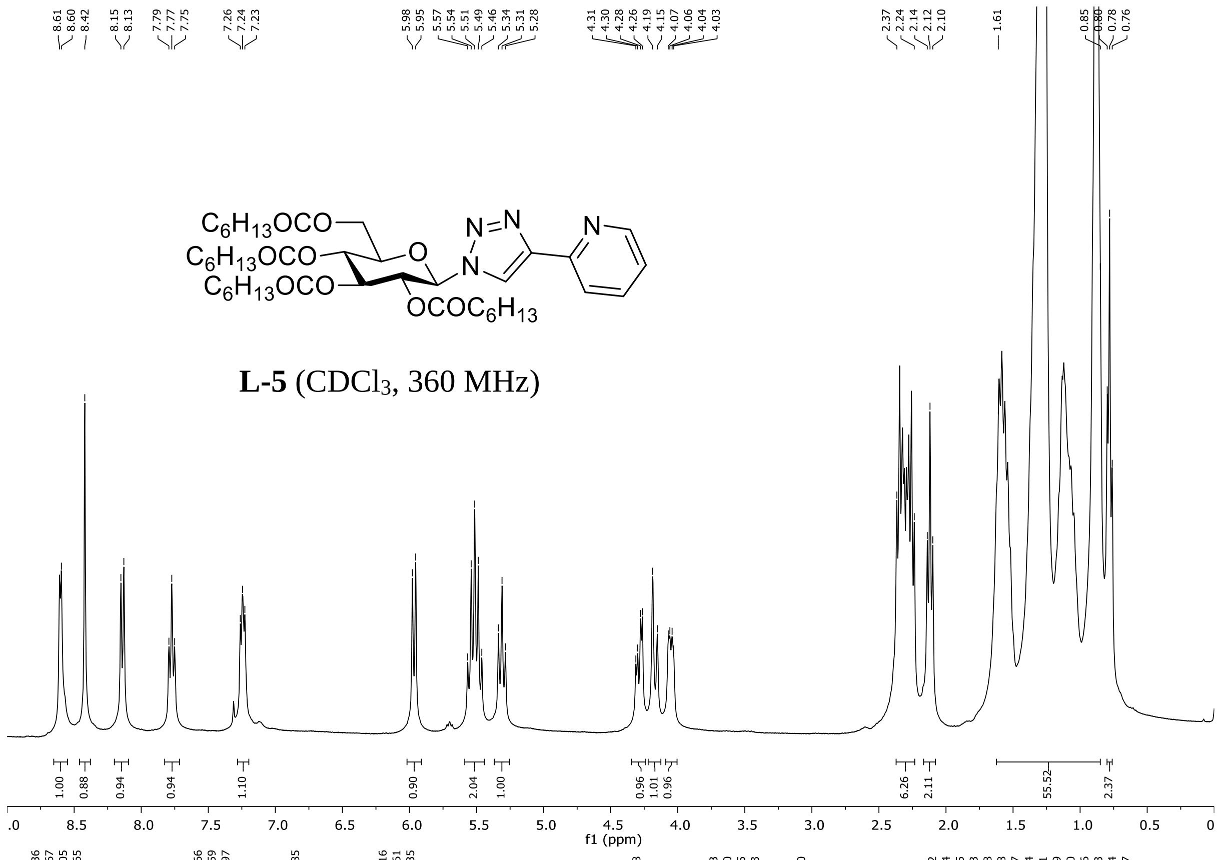
C6H13OCO
C6H13OCO
C6H13OCO
OCOC6H13
L-5 (CDCl3, 360 MHz)
f1 (ppm)

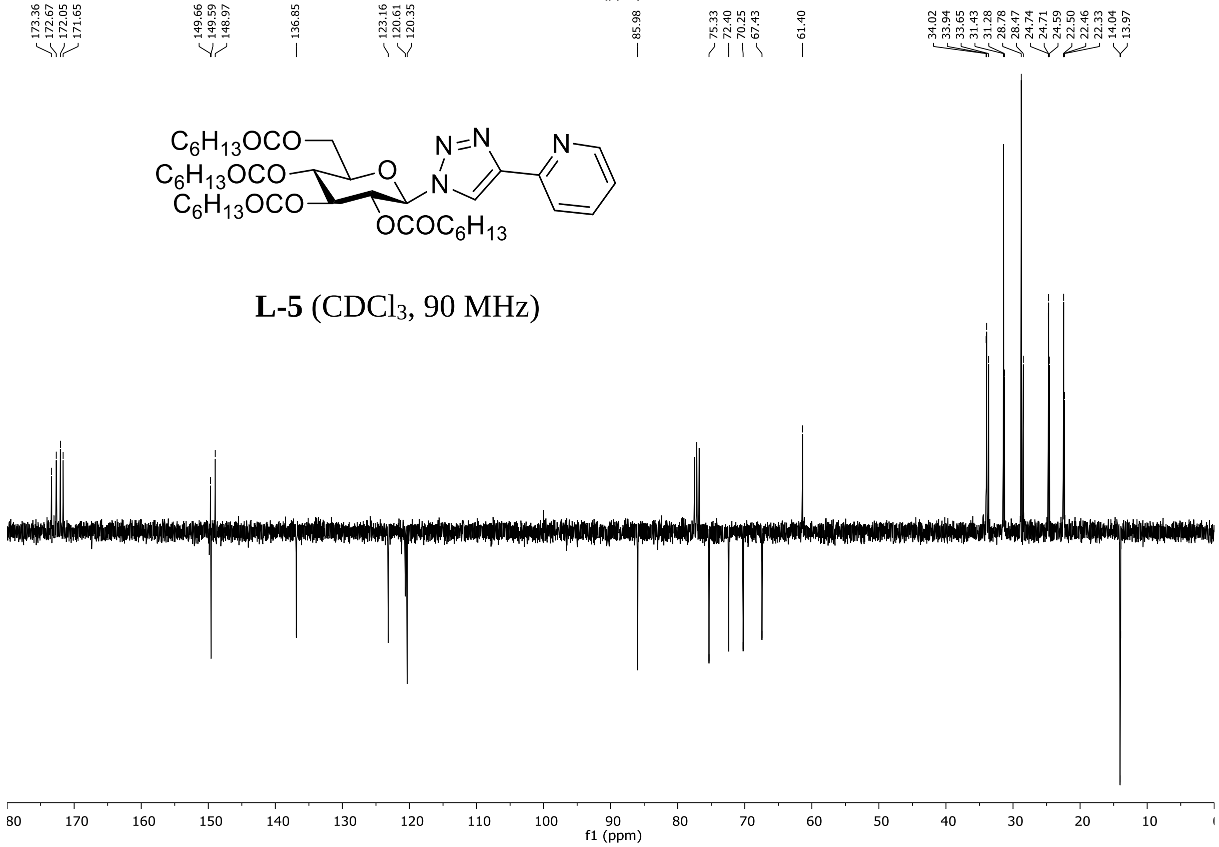
C6H13OCO
C6H13OCO
C6H13OCO
OCOC6H13
L-5 (CDCl3, 90 MHz)
f1 (ppm)

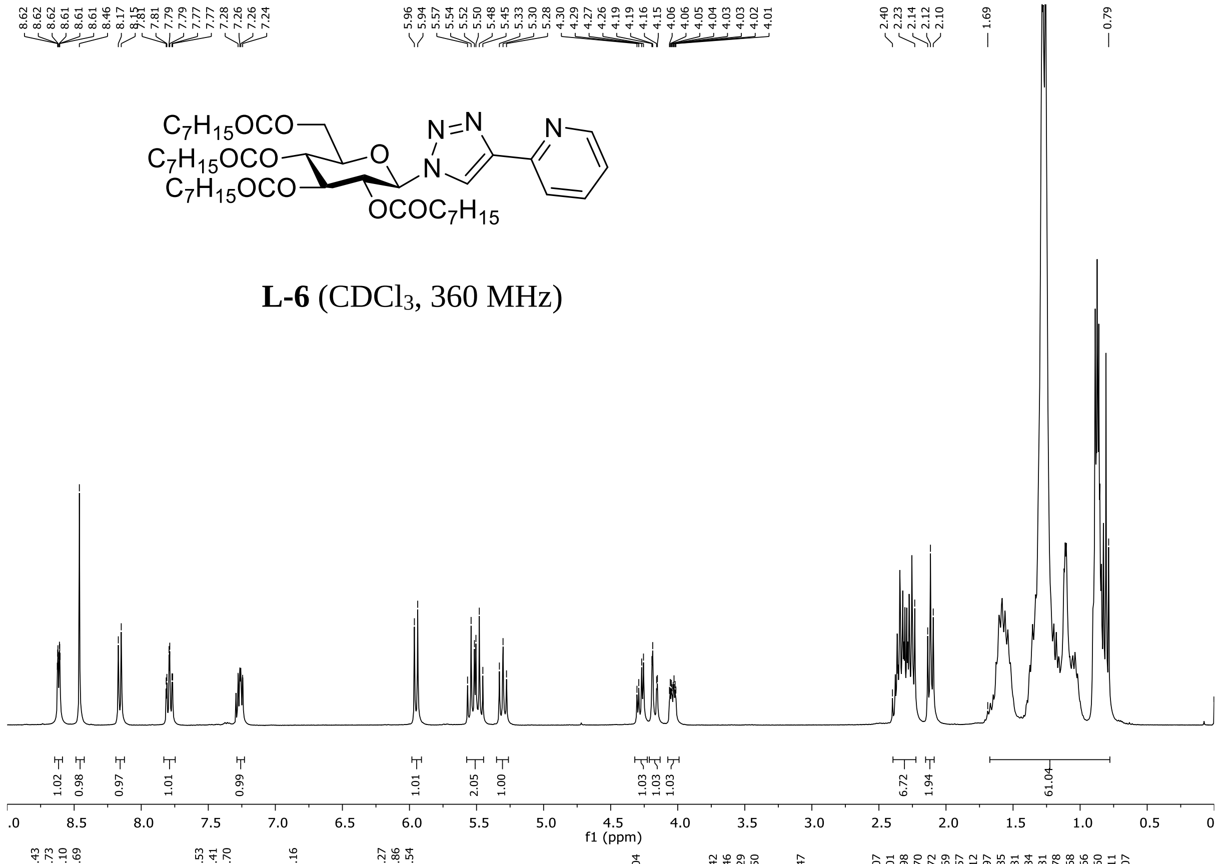

L-6 ($CDCl_3$, 360 MHz)

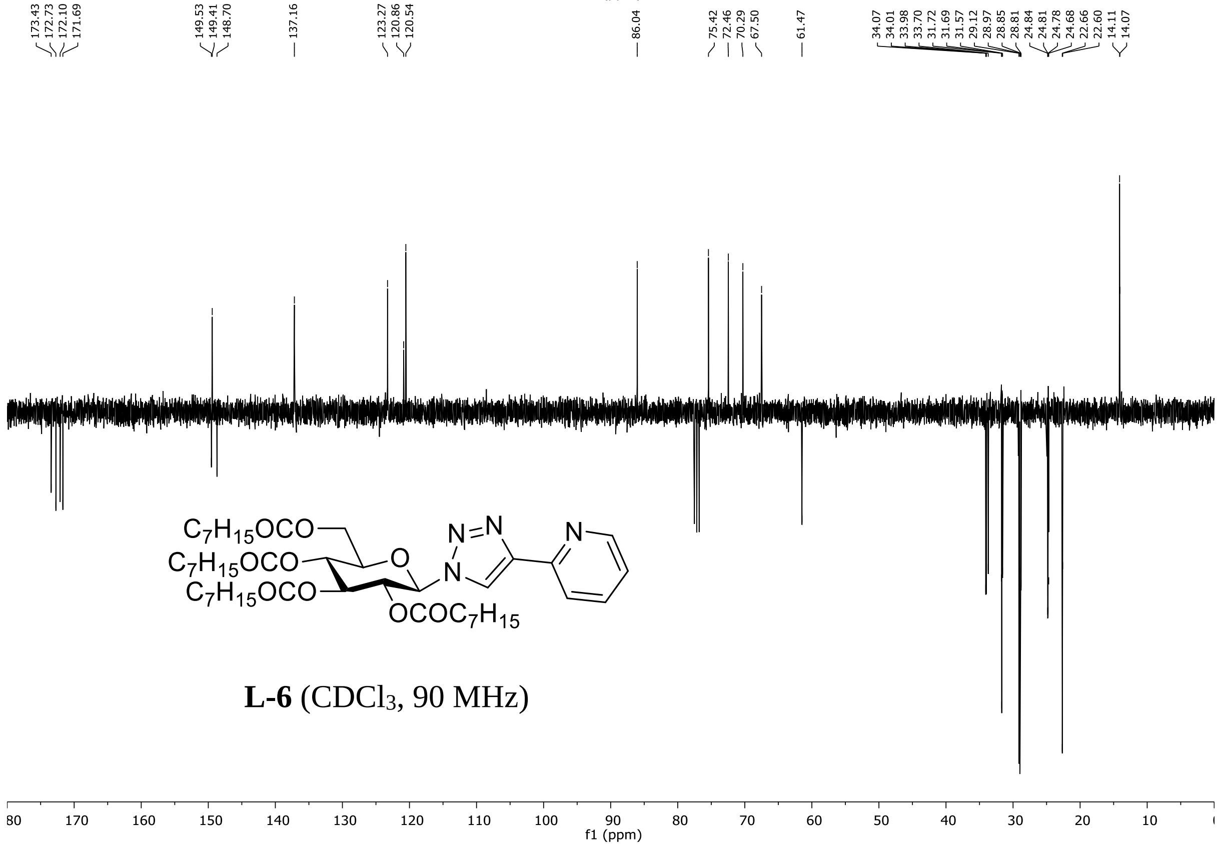

L-6 ($CDCl_3$, 90 MHz)

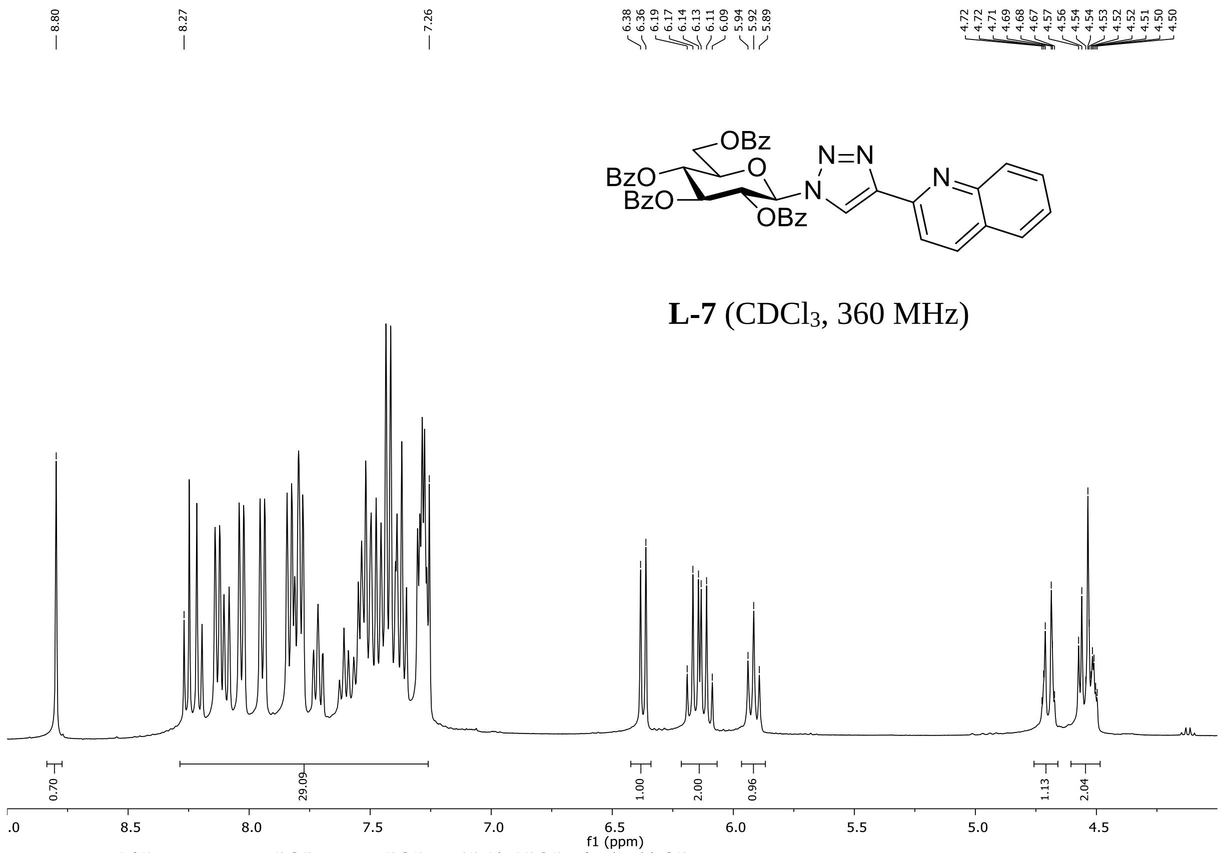
8.80
8.27
7.26
6.38 6.36 6.19 6.17 6.14 6.13 6.11 6.09 5.94 5.92 5.89
4.72 4.72 4.71 4.69 4.68 4.67 4.57 4.56 4.54 4.54 4.53 4.52 4.52 4.51 4.50 4.50
BzO
BzO
OBz
OBz
O
N
N=N
N
L-7 (CDCl3, 360 MHz)
0.70
29.09
1.00
2.00
0.96
1.13
2.04
f1 (ppm)

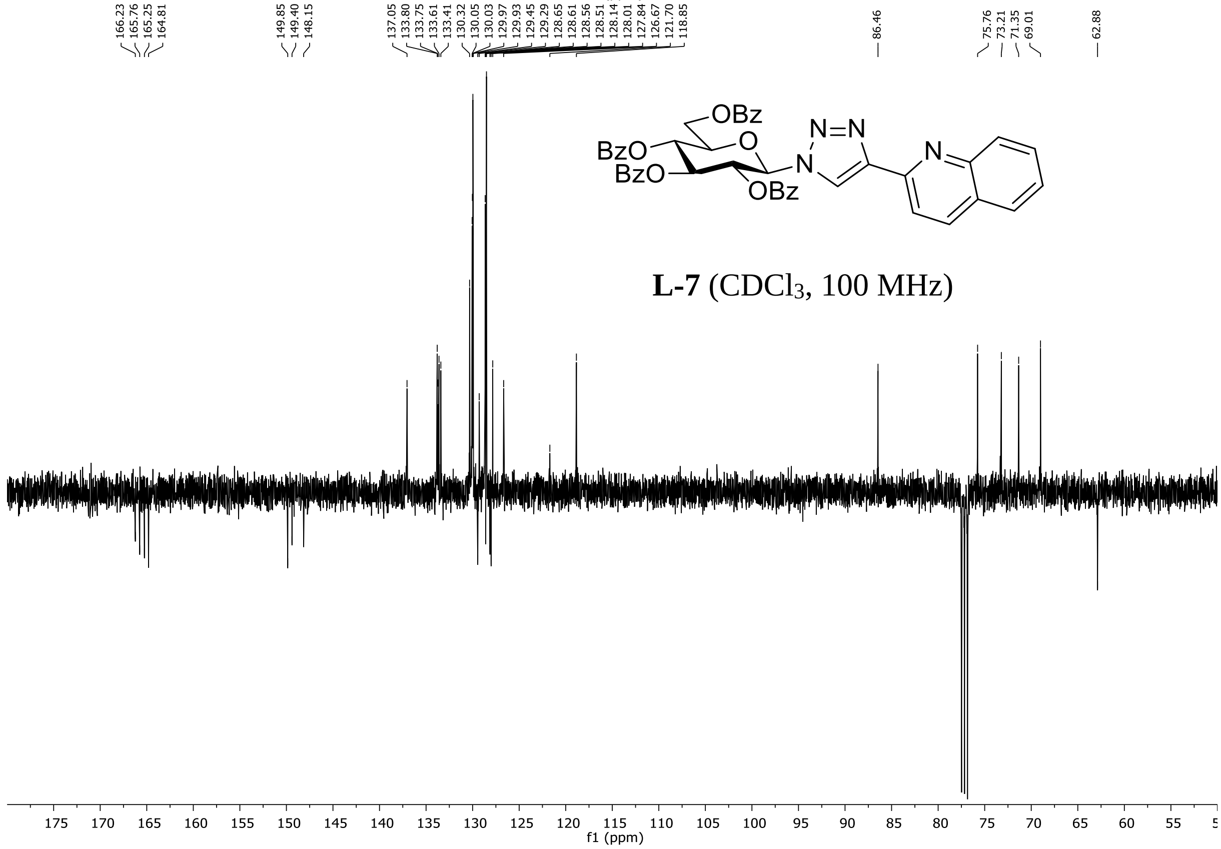
166.23 165.76 165.25 164.81
149.85 149.40 148.15
137.05 133.80 133.75 133.61 133.41 130.32 130.05 130.03 129.97 129.93 129.45 129.29 128.65 128.61 128.56 128.51 128.14 128.01 127.84 126.67 121.70 118.85
86.46
75.76 73.21 71.35 69.01
62.88
BzO
BzO
OBz
OBz
O
N
N=N
N
L-7 (CDCl3, 100 MHz)
f1 (ppm)

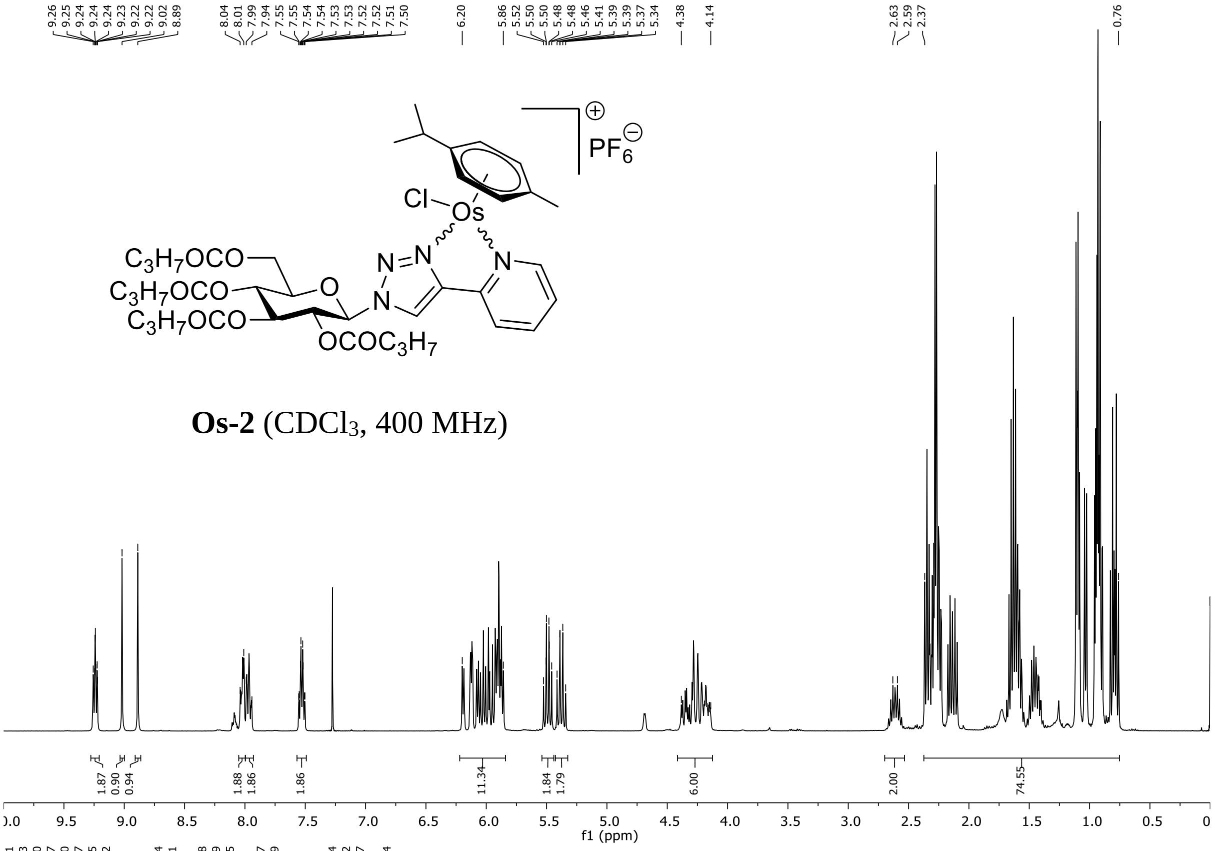

Os-2 ($CDCl_3$, 400 MHz)

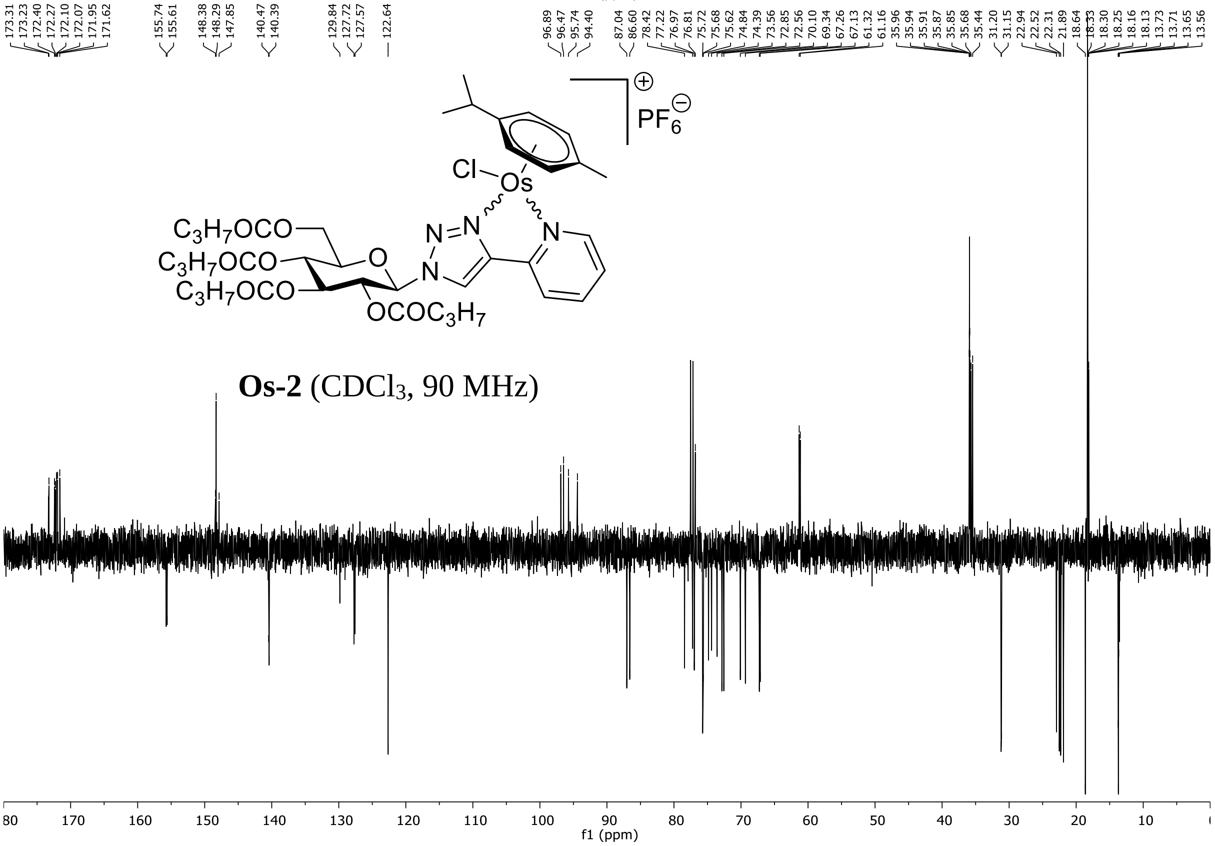

Os-2 ($CDCl_3$, 90 MHz)

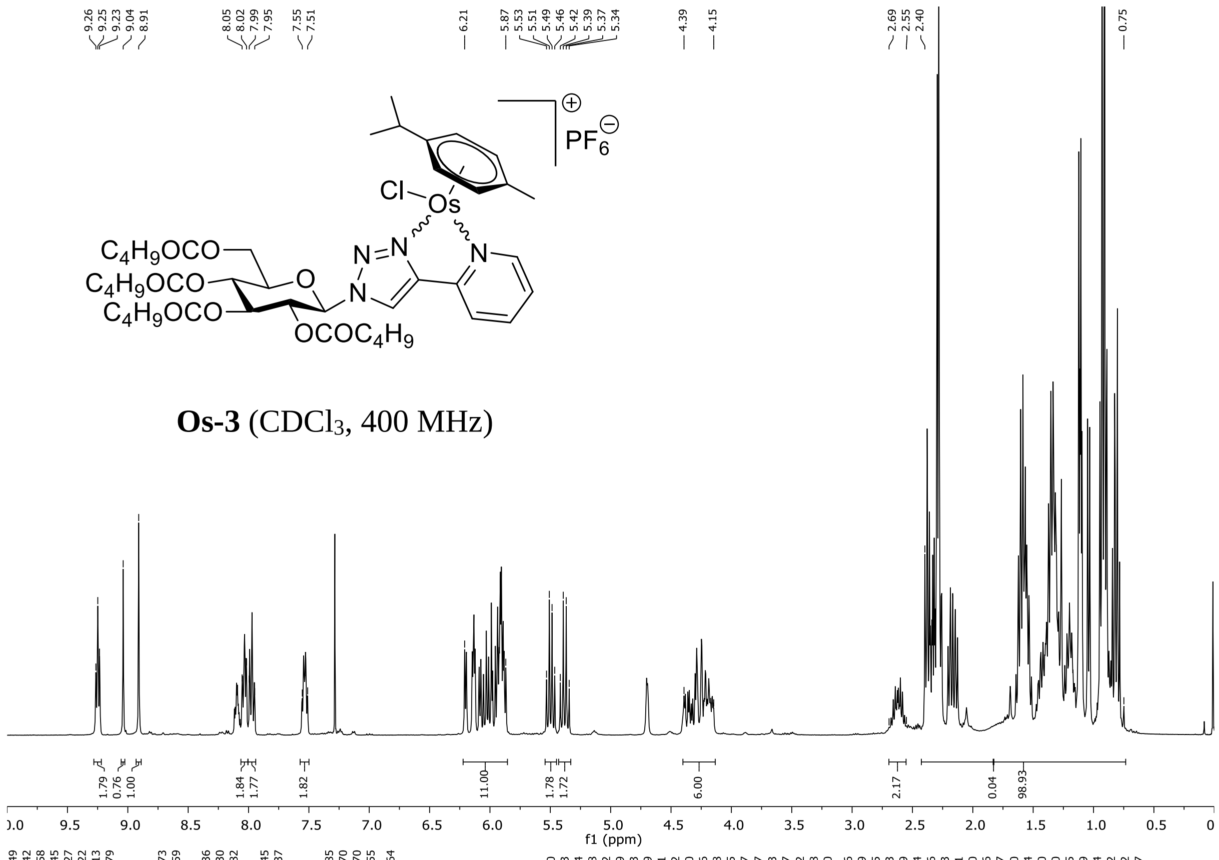
9.26 9.25 9.23 9.04 8.91 8.05 8.02 7.99 7.95 7.55 7.51 6.21 5.87 5.53 5.51 5.49 5.46 5.42 5.39 5.37 5.34 4.39 4.15 2.69 2.55 2.40 0.75
PF6
Cl
Os
N
C4H9OCO
O
OCOC4H9
Os-3 (CDCl3, 400 MHz)
1.79 0.76 1.00 1.84 1.77 1.82 11.00 1.78 1.72 6.00 2.17 0.04 98.93
f1 (ppm)

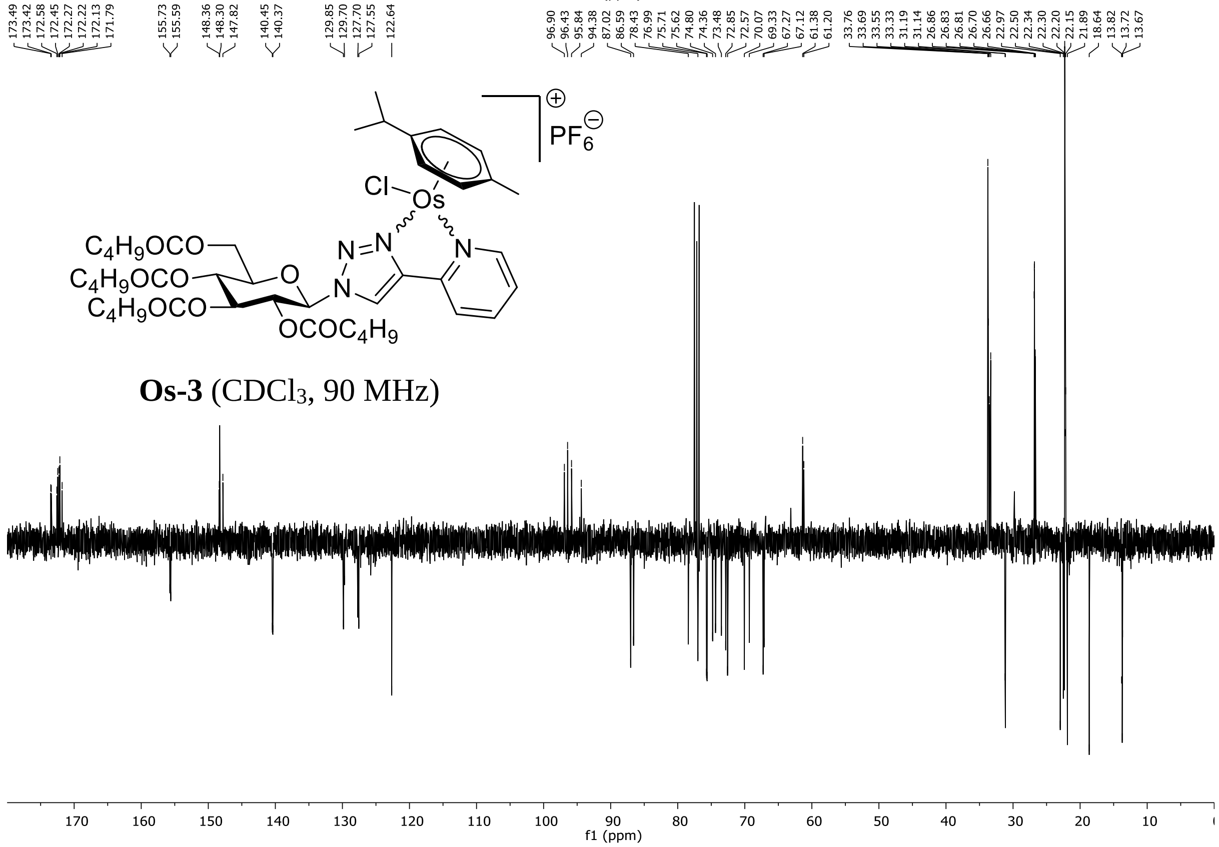
173.49 173.42 172.58 172.45 172.27 172.22 172.13 171.79 155.73 155.59 148.36 148.30 147.82 140.45 140.37 129.85 129.70 127.70 127.55 122.64 96.90 96.43 95.84 94.38 87.02 86.59 78.43 76.99 75.71 75.62 74.80 74.36 73.48 72.85 72.57 70.07 69.33 67.27 67.12 61.38 61.20 33.76 33.69 33.55 33.33 31.19 31.14 26.86 26.83 26.81 26.70 26.66 22.97 22.50 22.34 22.30 22.20 22.15 21.89 18.64 13.82 13.72 13.67
PF6
Cl
Os
N
C4H9OCO
O
OCOC4H9
Os-3 (CDCl3, 90 MHz)
f1 (ppm)

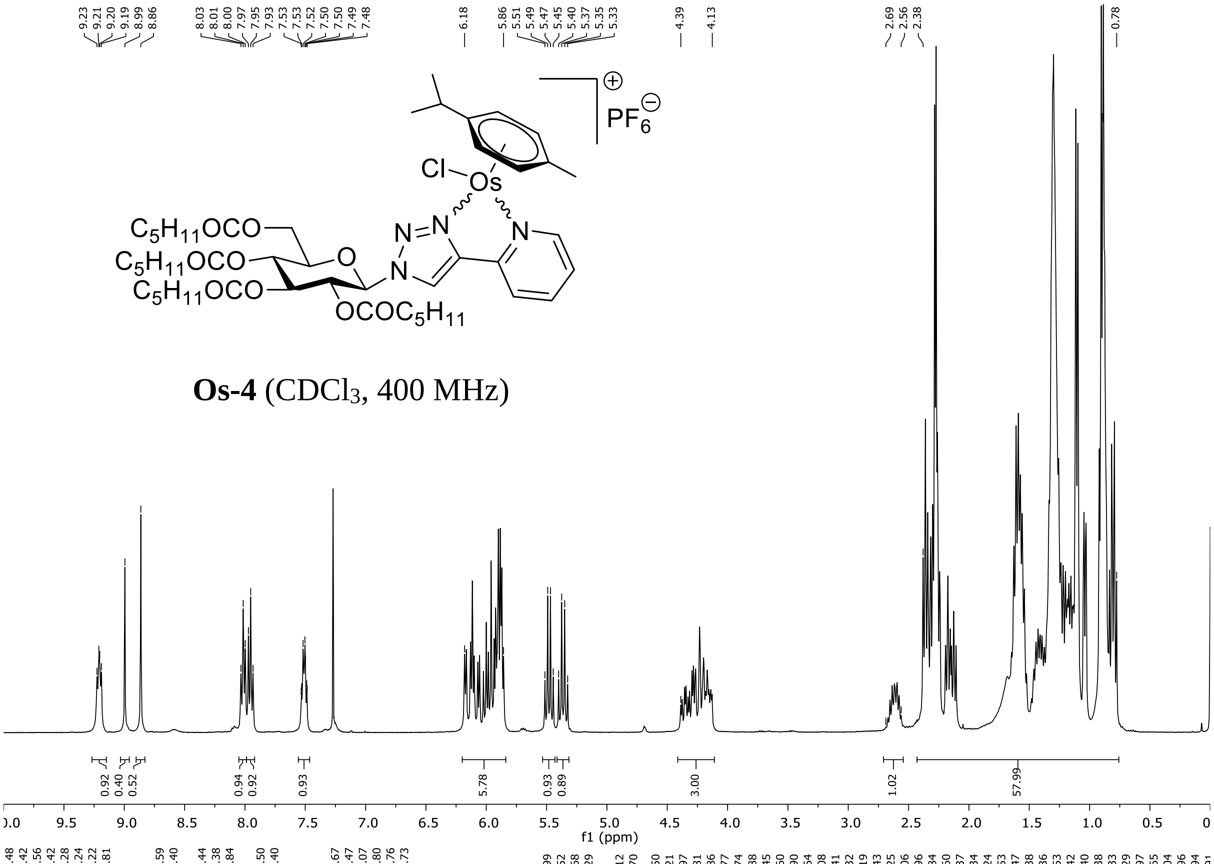

Os-4 ($CDCl_3$, 400 MHz)

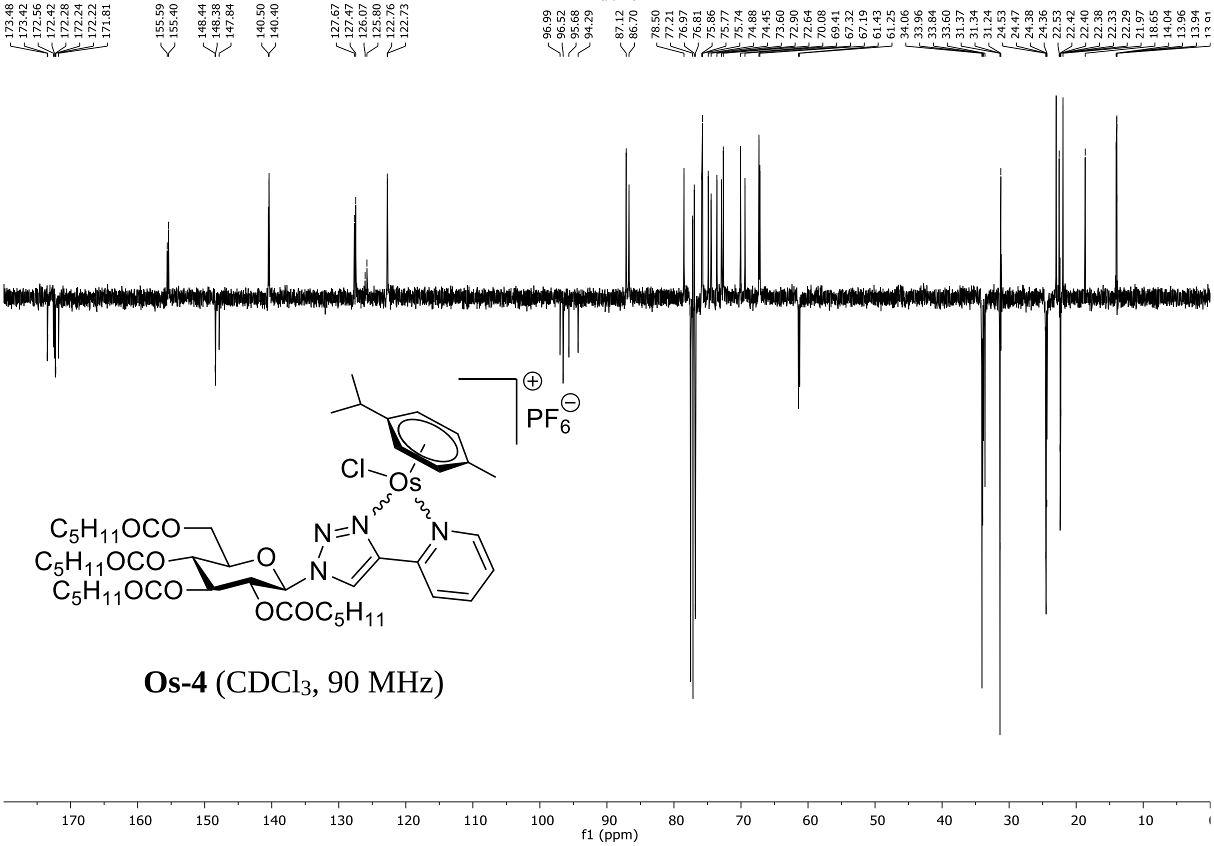

Os-4 ($CDCl_3$, 90 MHz)

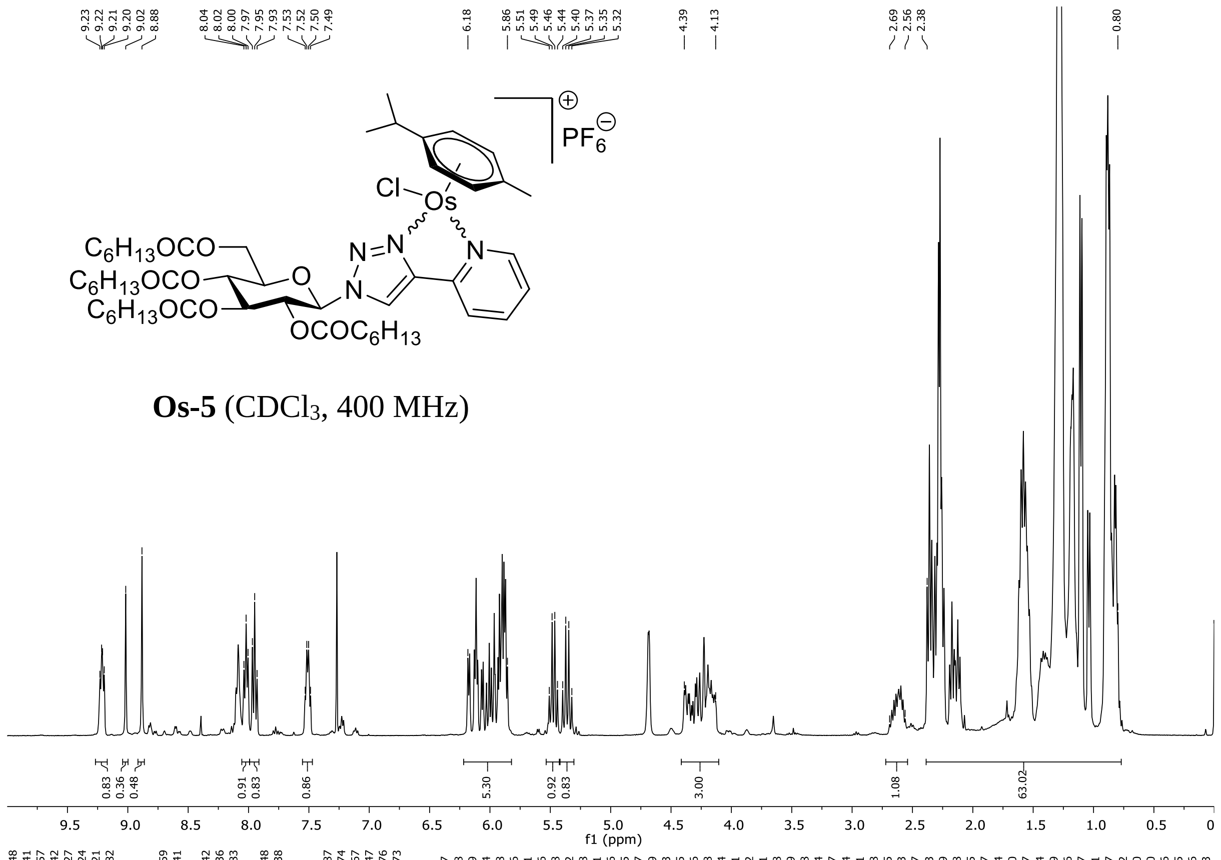
Os-5 (CDCl3, 400 MHz)
f1 (ppm)

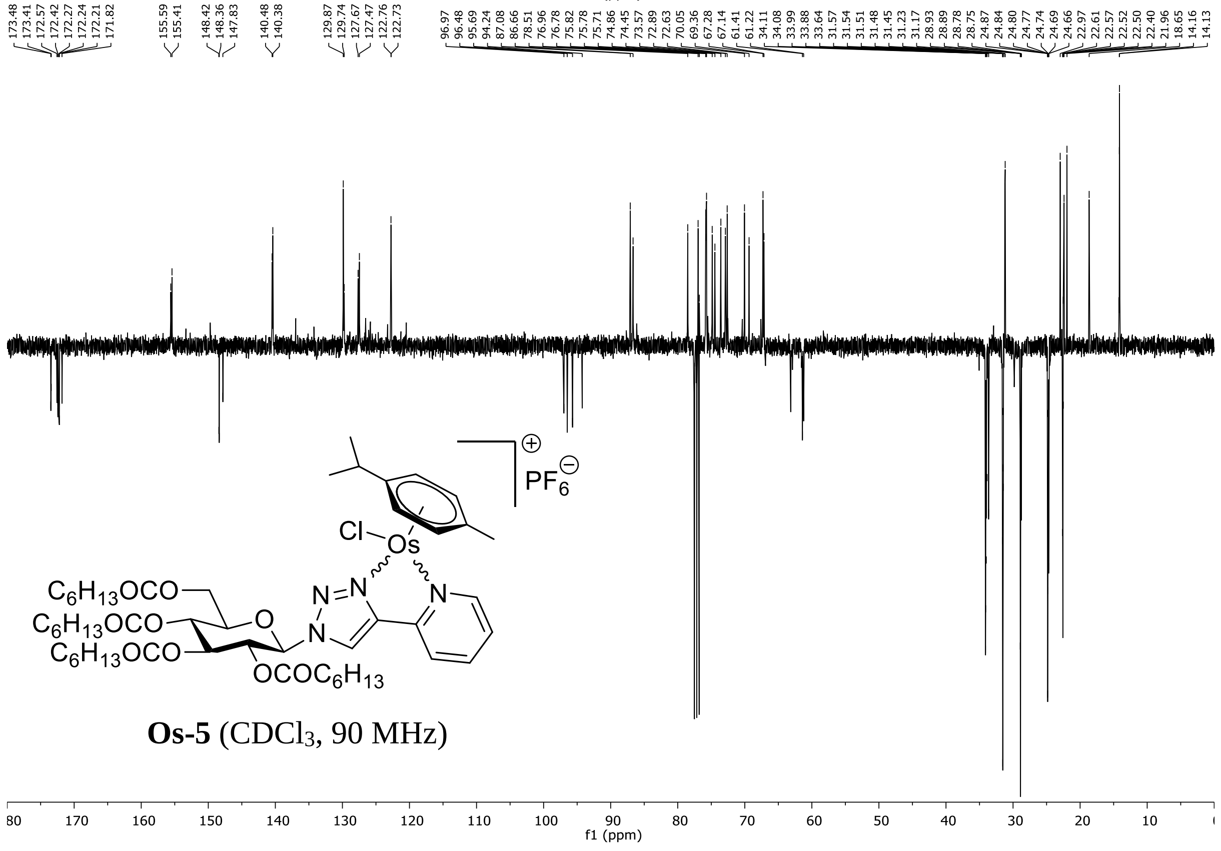
Os-5 (CDCl3, 90 MHz)
f1 (ppm)

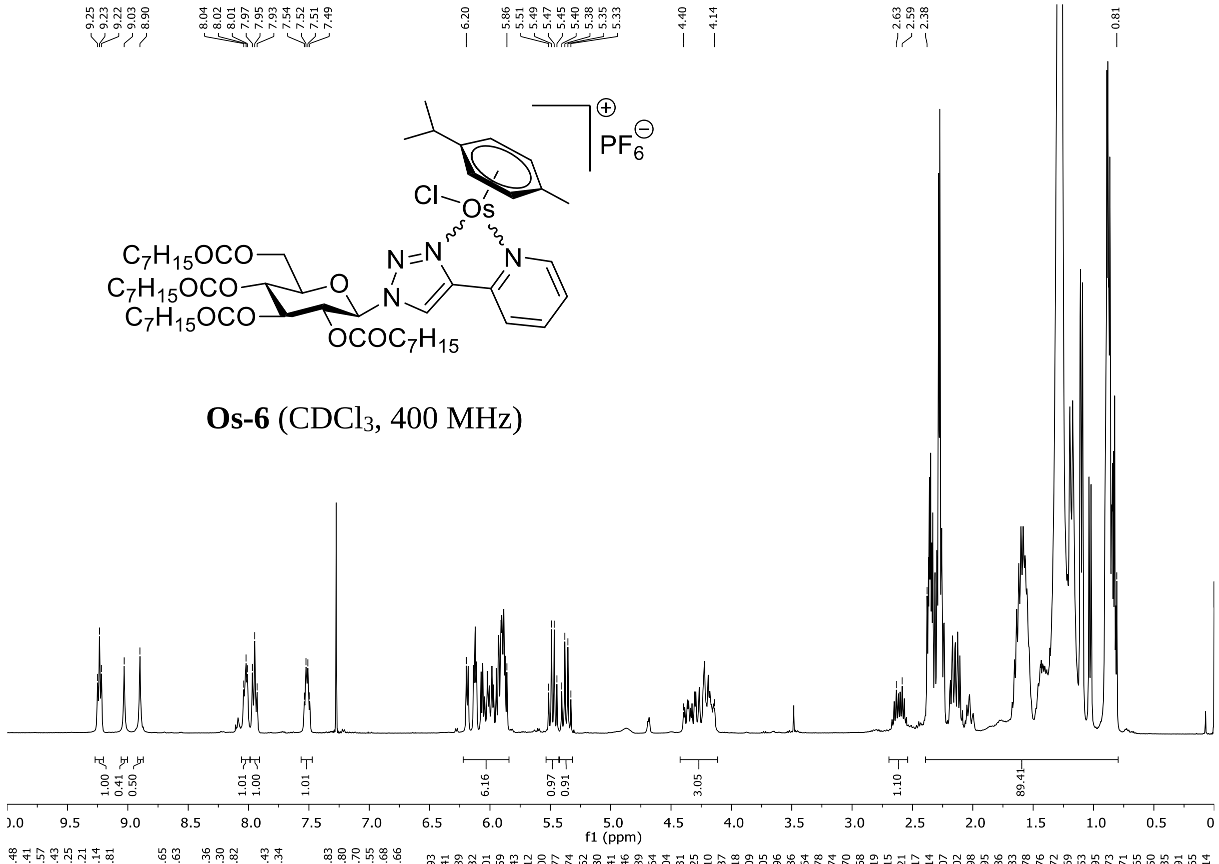

Os-6 (CDCl$_3$, 400 MHz)

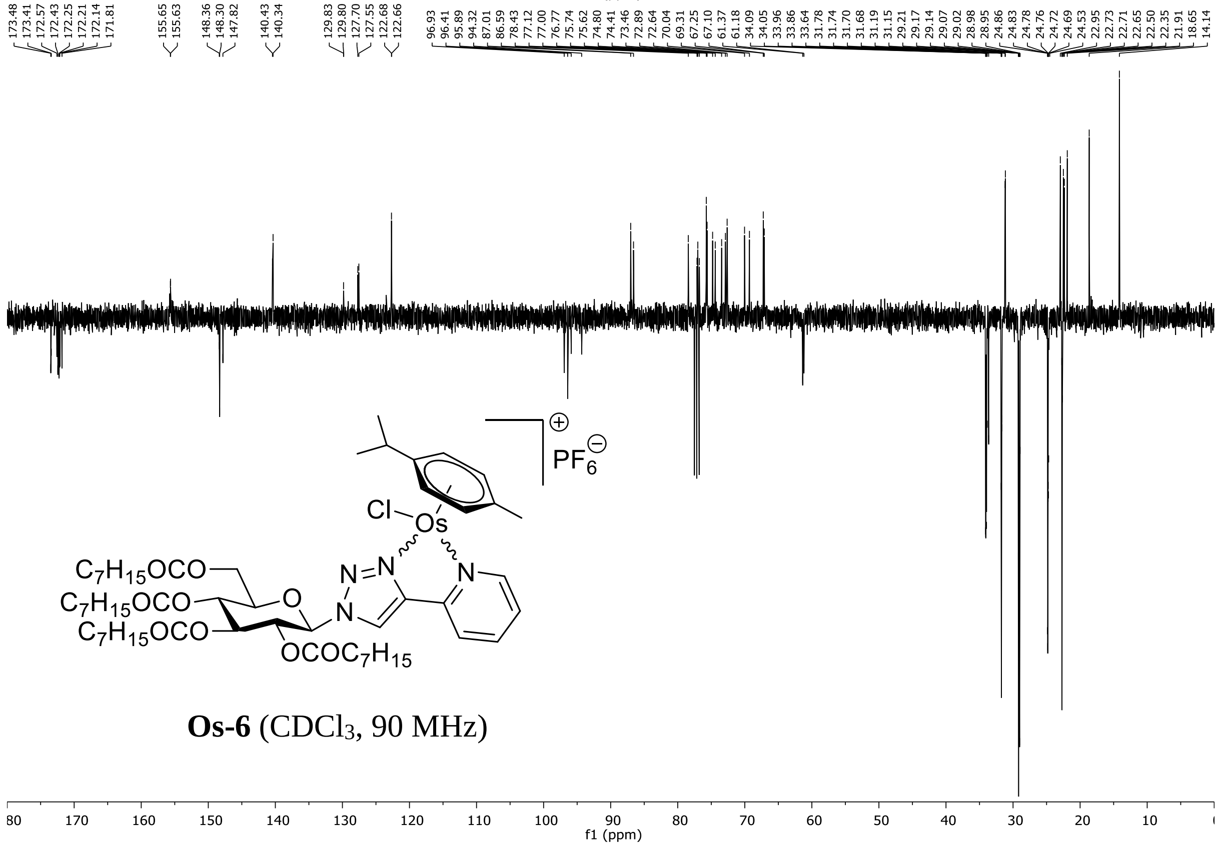

Os-6 (CDCl$_3$, 90 MHz)

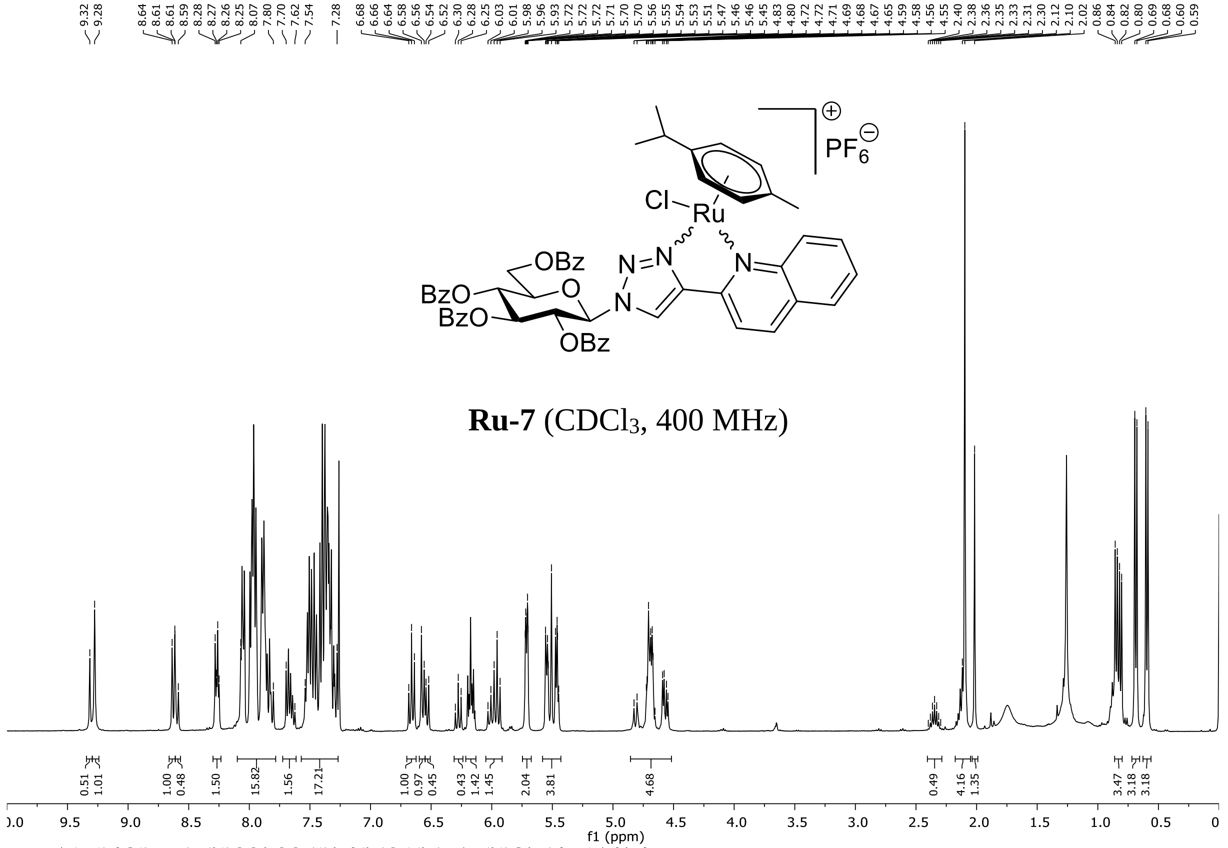

Ru-7 ($CDCl_3$, 400 MHz)

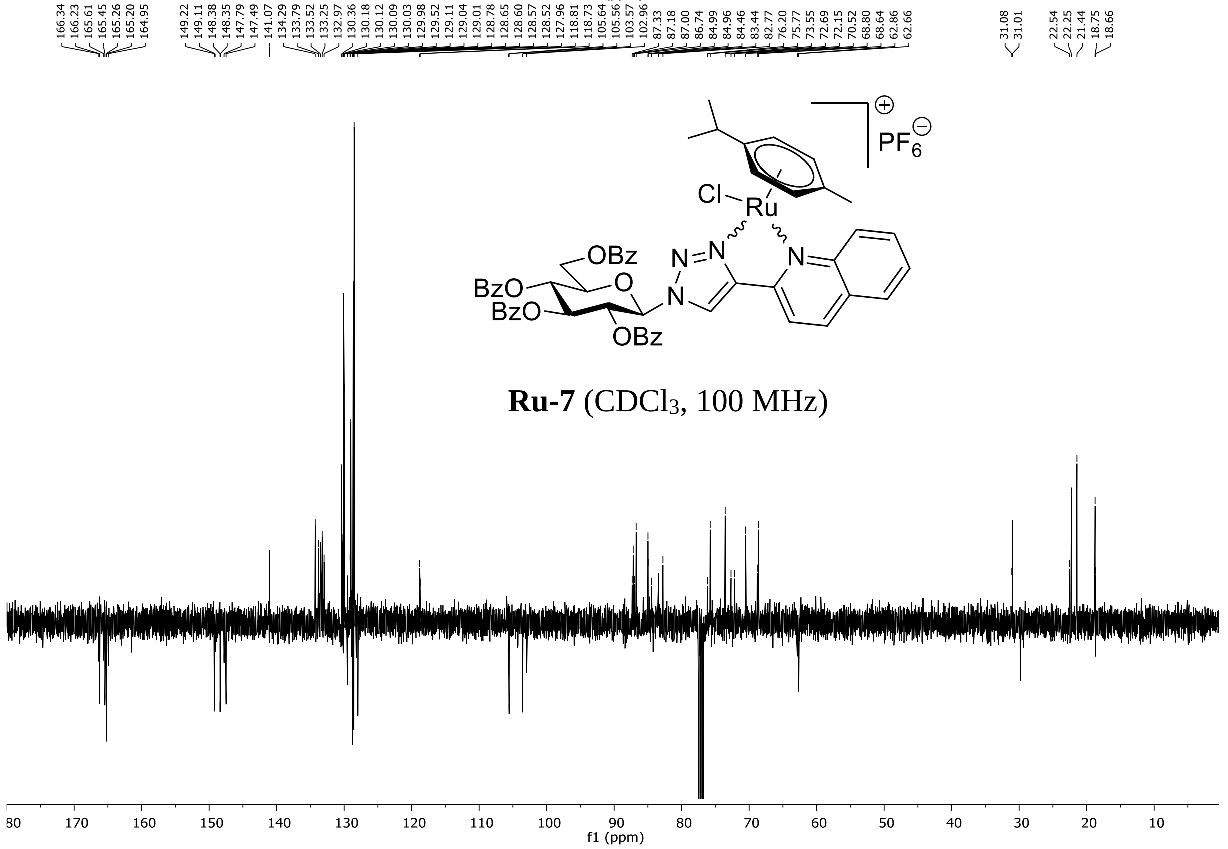

Ru-7 ($CDCl_3$, 100 MHz)

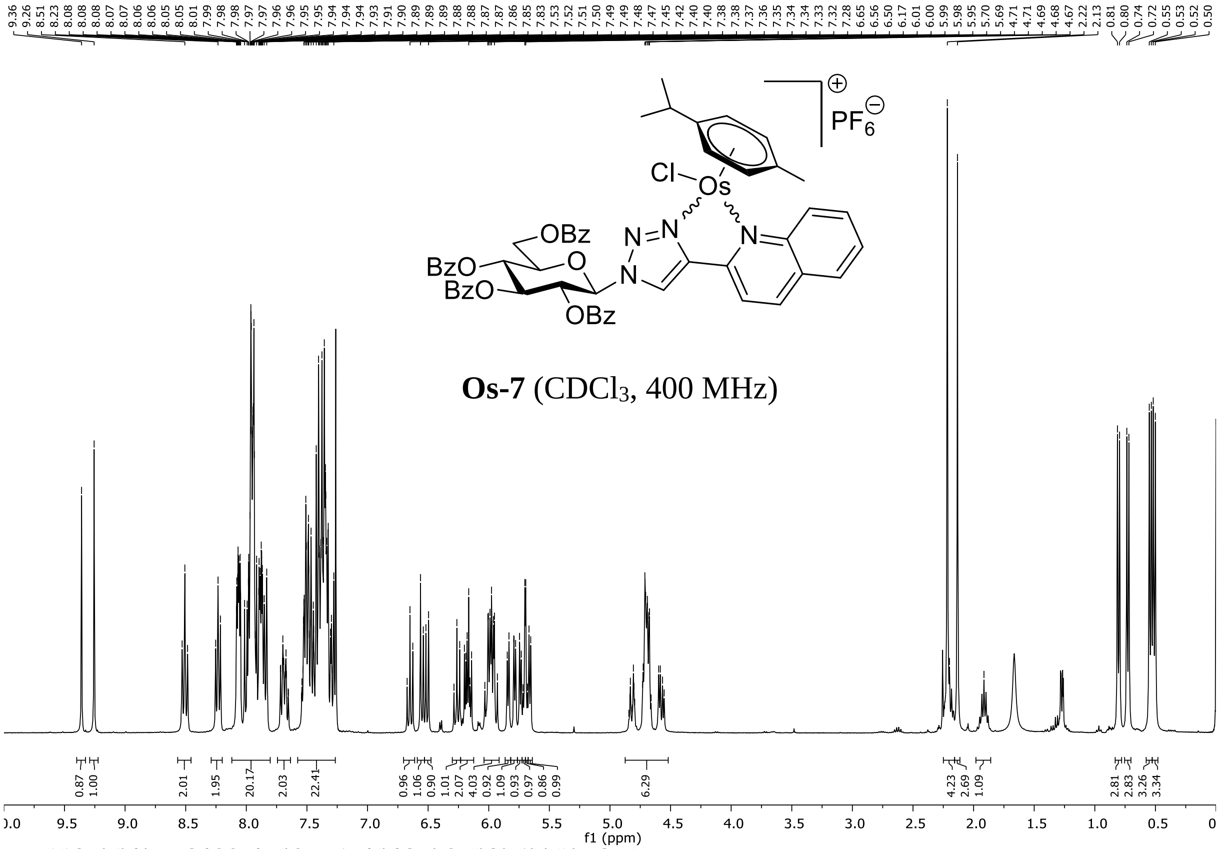

Os-7 ($CDCl_3$, 400 MHz)

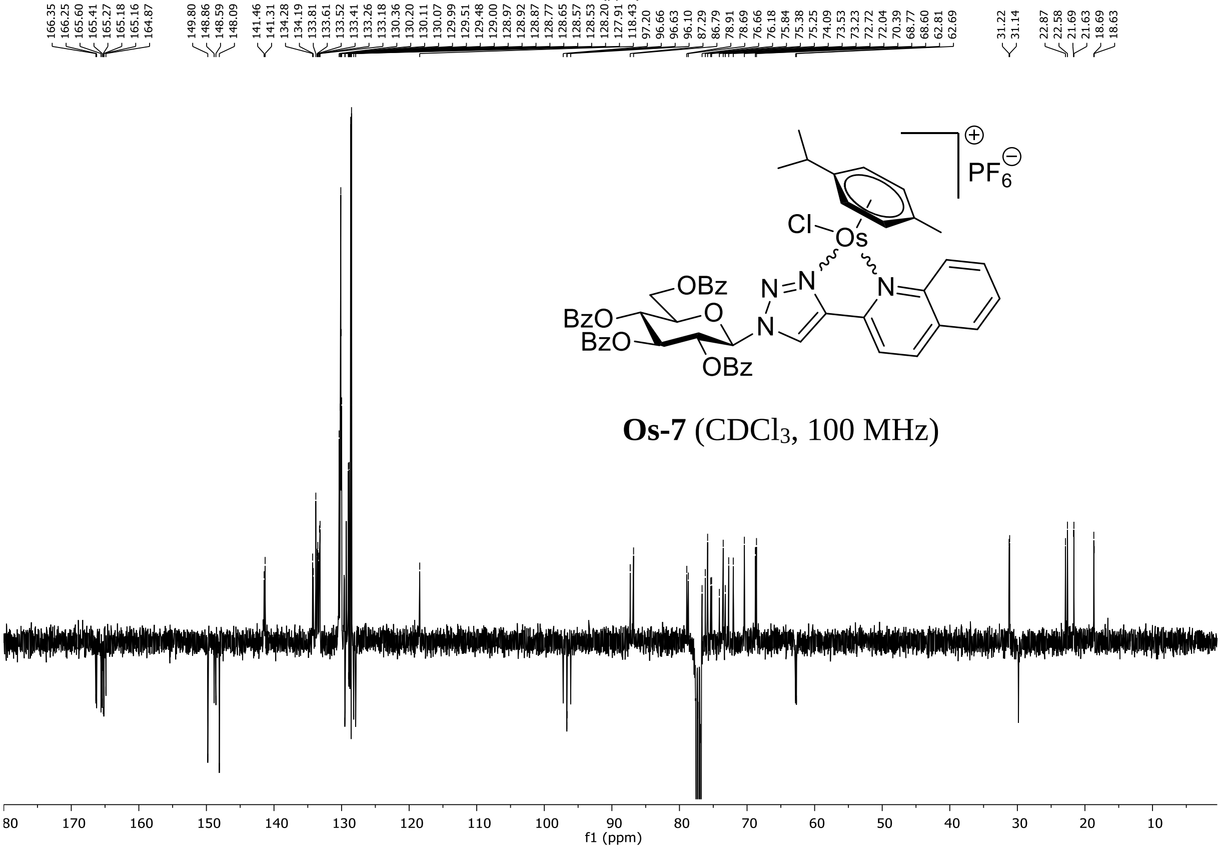

Os-7 ($CDCl_3$, 100 MHz)

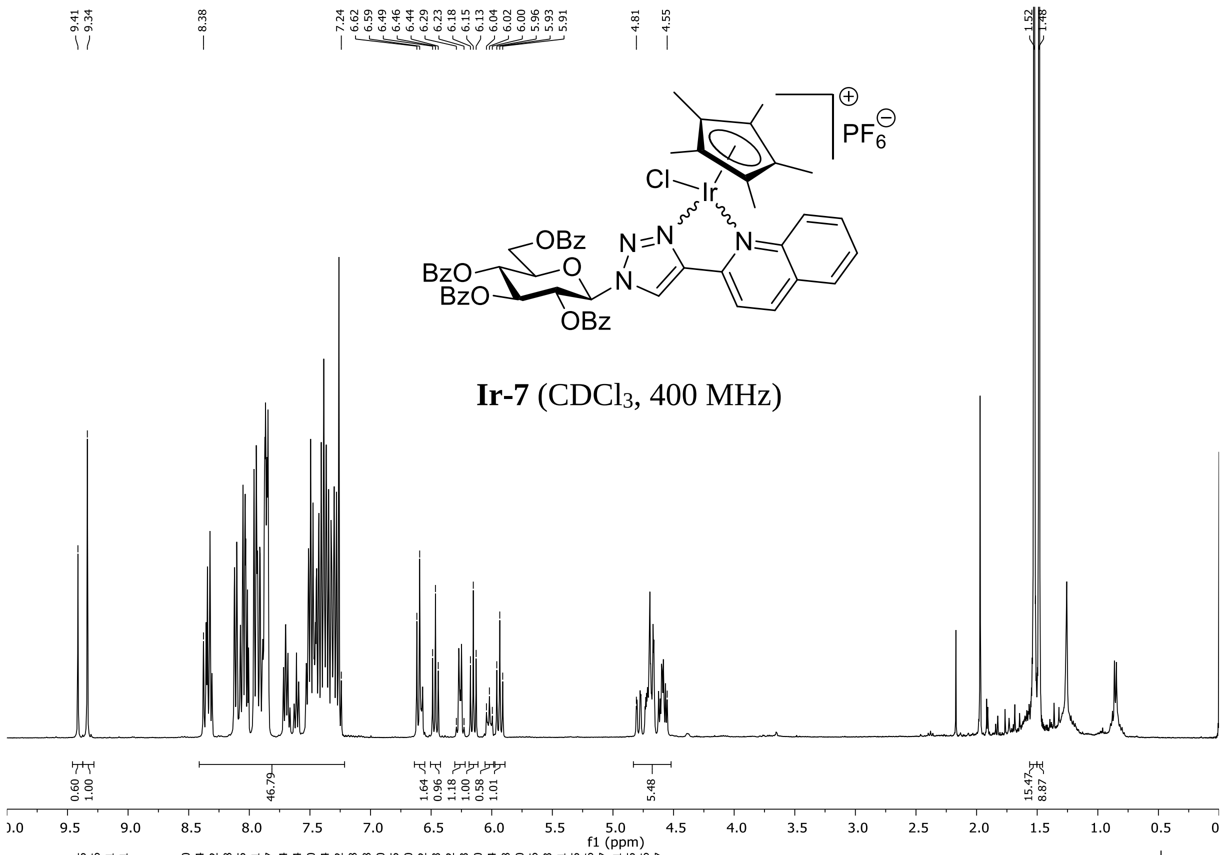
Ir-7 (CDCl3, 400 MHz)
OBz
BzO
BzO
OBz
PF6
Cl
Ir
f1 (ppm)

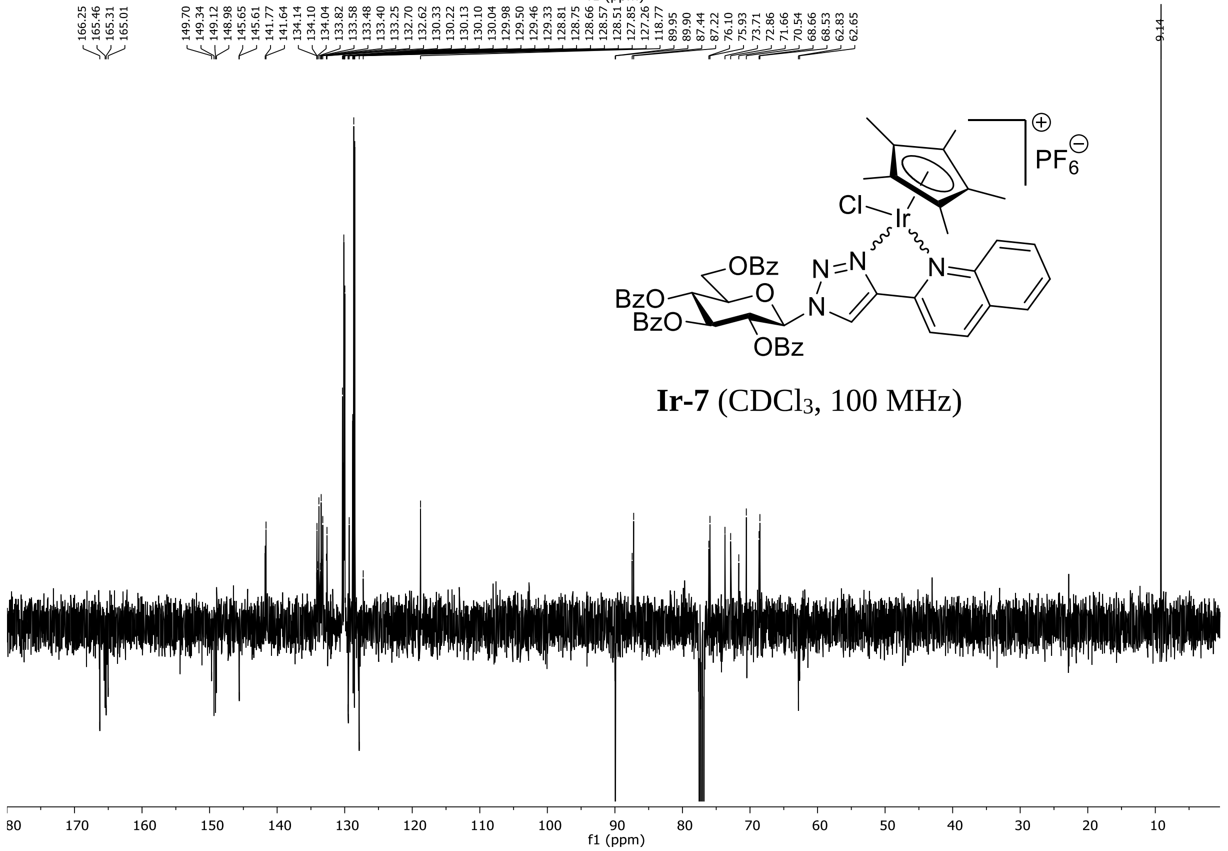
Ir-7 (CDCl3, 100 MHz)
OBz
BzO
BzO
OBz
PF6
Cl
Ir
f1 (ppm)

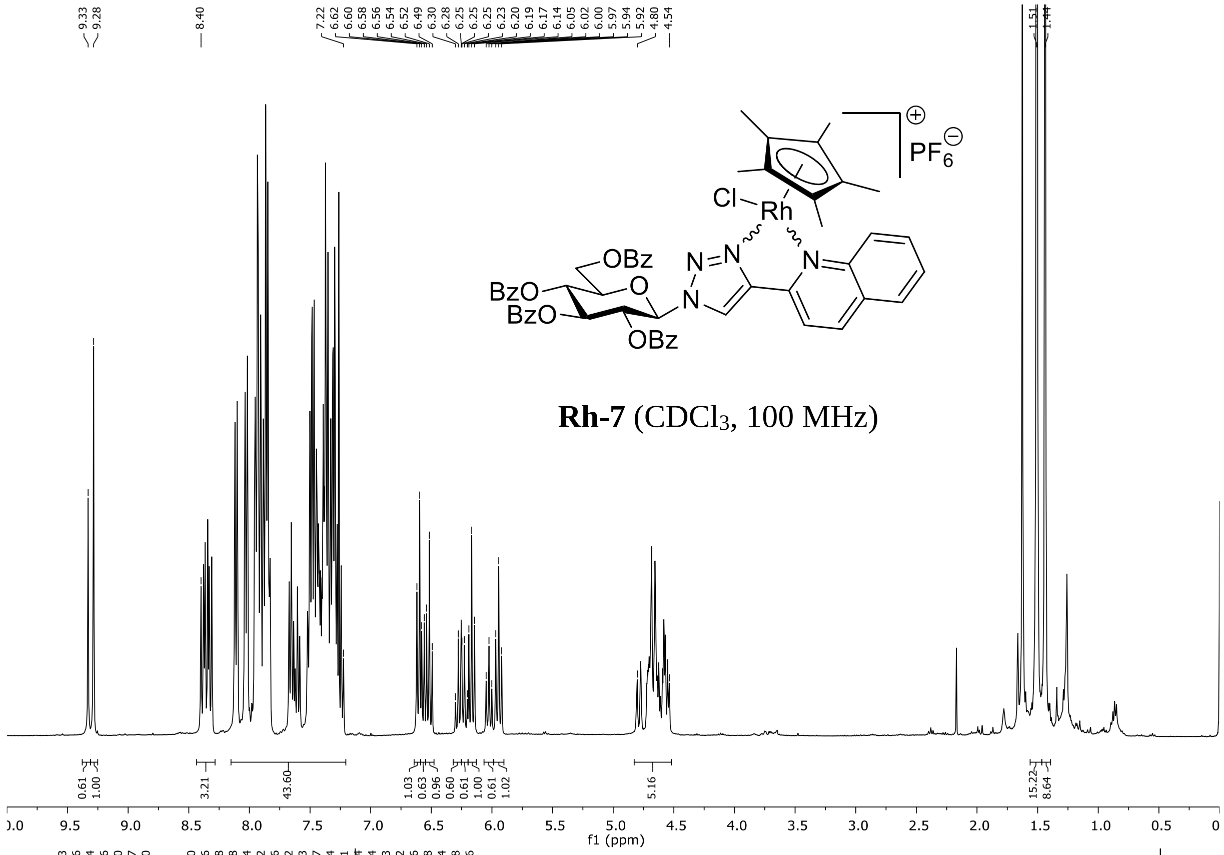
9.33
9.28
8.40
7.22
6.62
6.60
6.58
6.56
6.54
6.52
6.49
6.30
6.28
6.25
6.25
6.25
6.23
6.20
6.19
6.17
6.14
6.05
6.02
6.00
5.97
5.94
5.92
4.80
4.54
1.51
1.44
PF6
Cl
Rh
N
N=N
N
OBz
BzO
BzO
O
OBz
Rh-7 (CDCl3, 100 MHz)
0.61
1.00
3.21
43.60
1.03
0.63
0.96
0.60
0.61
1.00
0.61
1.02
5.16
15.22
8.64
f1 (ppm)

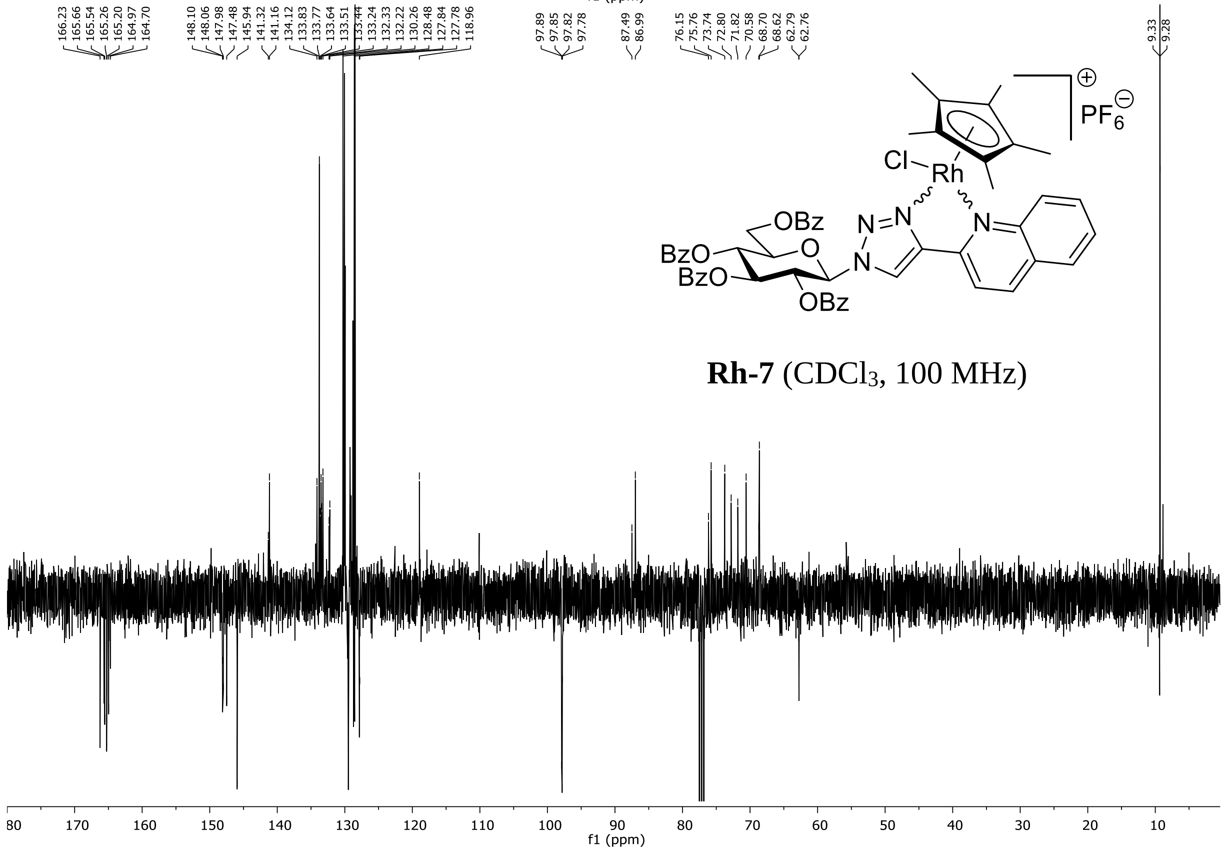
166.23
165.66
165.54
165.26
165.20
164.97
164.70
148.10
148.06
147.98
147.48
145.94
141.32
141.16
134.12
133.83
133.77
133.64
133.51
133.44
133.24
132.33
132.22
130.26
128.48
127.84
127.78
118.96
97.89
97.85
97.82
97.78
87.49
86.99
76.15
75.76
73.74
72.80
71.82
70.58
68.70
68.62
62.79
62.76
9.33
9.28
PF6
Cl
Rh
N
N=N
N
OBz
BzO
BzO
O
OBz
Rh-7 (CDCl3, 100 MHz)
f1 (ppm)

3. **Table S1.** Distribution coefficient of the synthesized complexes (logD)		
**Complex**	**Structure**	**logD**
**Os-2**	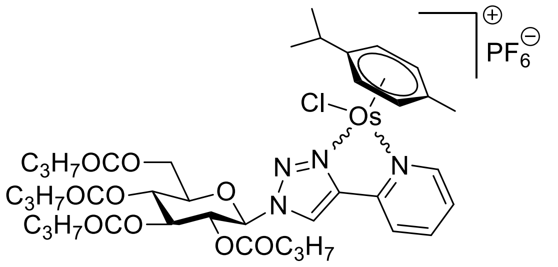	+2.5
**Os-3**	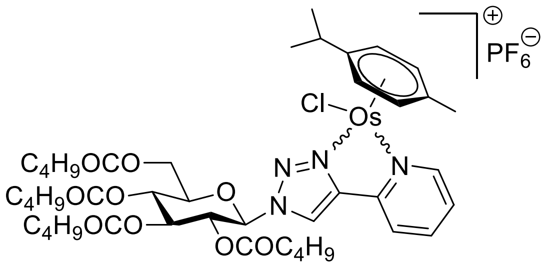	+2.6
**Os-4**	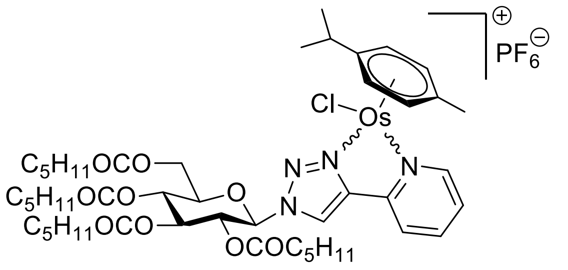	+2.7
**Os-5**	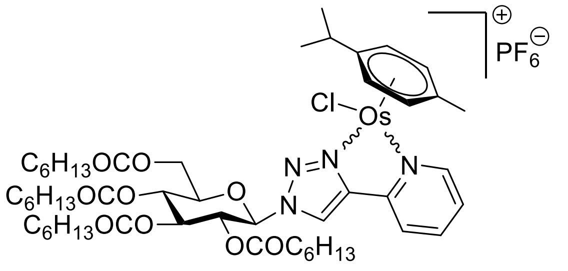	+2.7
**Os-6**	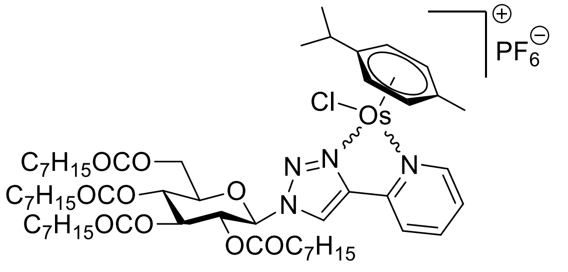	+2.8

**Ru-7**	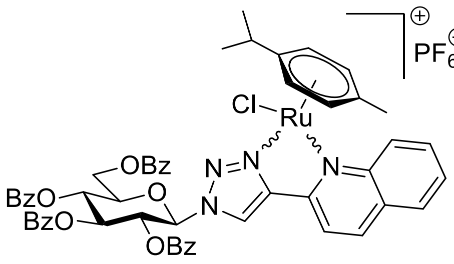	+2.2
**Os-7**	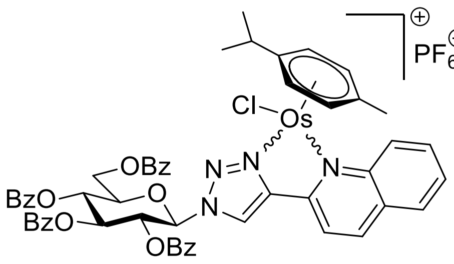	+2.3
**Ir-7**	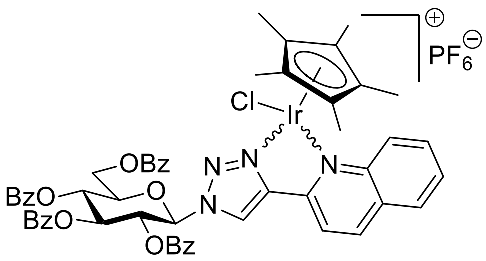	+2.2
**Rh-7**	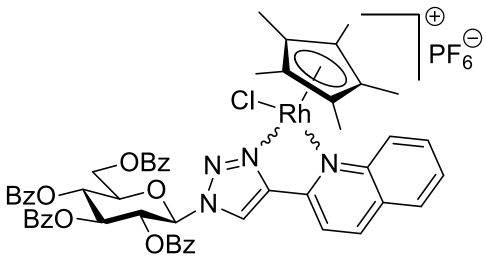	+1.6

5. References

Godó, A.J., Bényei, A.C., Duff, B., Egan, D.A., and Buglyó, P. (2012). Synthesis and X-ray diffraction structures of novel half-sandwich Os(ii)-and Ru(ii)-hydroxamate complexes. *RSC Advances* 2**,** 1486-1495.

Kacsir, I., Sipos, A., Ujlaki, G., Buglyo, P., Somsak, L., Bai, P., and Bokor, E. (2021). Ruthenium Half-Sandwich Type Complexes with Bidentate Monosaccharide Ligands Show Antineoplastic Activity in Ovarian Cancer Cell Models through Reactive Oxygen Species Production. *International Journal of Molecular Sciences* 22**,** 10454.
